# Global trends and inequalities in eye cancer burden: a comprehensive analysis based on the global burden of disease study

**DOI:** 10.3389/fmed.2025.1638733

**Published:** 2025-08-14

**Authors:** Jianhao Bai, Zhongqi Wan, Yan Gao, Qing Peng

**Affiliations:** ^1^Department of Ophthalmology, Shanghai East Hospital Affiliated to Tongji University, Tongji University School of Medicine, Shanghai, China; ^2^Department of Ophthalmology, Shanghai Tenth People's Hospital Affiliated to Tongji University, Tongji University School of Medicine, Shanghai, China; ^3^Department of Ophthalmology, Shanxi Eye Hospital Affiliated to Shanxi Medical University, Taiyuan, China

**Keywords:** eye cancer, epidemiology, inequality, global burden of disease, disability-adjusted life years

## Abstract

**Background:**

Eye cancer is a significant threat to vision and survival because of its location, diagnostic challenges, and aggressive nature. However, its global epidemiology, especially regarding differences across countries, age groups, and sex, is not well-studied.

**Methods:**

This study analyzed data from the Global Burden of Disease Study 2021 to evaluate trends in eye cancer, focusing on incidence, prevalence, mortality, and disability-adjusted life years (DALYs) across 204 countries from 1990 to 2021. Age-standardized rates and estimated annual percentage changes were used to assess trends over time. Disparities were examined by sociodemographic index (SDI), sex, and age, with concentration and slope index analyses assessing development-and sex-related inequalities.

**Results:**

From 1990 to 2021, the global burden of eye cancer showed an overall increase in incidence and prevalence, with notable geographic and sociodemographic variations. Sociodemographic analysis revealed persistent inequalities, with higher detection-related prevalence and incidence in developed regions and greater mortality and disability in less developed areas. Age-specific prevalence demonstrated a rightward shift, with older populations, particularly those aged ≥65 years, carrying the largest burden. Sex disparities were also evident, as men generally exhibited higher incidence and prevalence rates, while women in low-SDI regions faced a disproportionate share of mortality and DALY burden.

**Conclusion:**

This study highlights significant global disparities in eye cancer, influenced by sociodemographic factors, sex, and age. Urgent investment in diagnostic infrastructure, equitable care, and sex-sensitive measures is essential to reduce preventable vision loss and cancer deaths.

## Introduction

According to GLOBOCAN 2020, an estimated 19.3 million new cancer cases and 10 million cancer-related deaths occurred worldwide in 2020 alone, highlighting the growing global burden of malignancies (1). Despite accounting for less than 0.1% of all new cancer diagnoses globally, eye cancer represents a particularly challenging disease category due to its anatomical location and clinical consequences. Eye cancer comprises a heterogeneous group of malignancies originating from intraocular structures, ocular adnexa, and adjacent tissues, including the uvea, retina, eyelids, conjunctiva, and lacrimal glands (2–5). Although these malignancies are relatively rare compared to other solid tumors, their clinical implications are disproportionately severe. Even when confined to the eye, these cancers can result in irreversible vision loss, facial disfigurement, or necessitate enucleation, thereby significantly impacting visual function, psychosocial wellbeing, and overall quality of life (6, 7). Certain subtypes, such as uveal melanoma in adults and retinoblastoma in children, exhibit notable aggressiveness, characterized by high metastatic potential and significant mortality if not promptly diagnosed and treated (8, 9). The management of ocular cancer typically demands highly specialized, multidisciplinary care, which may not be readily available in many regions globally (10, 11). In low-resource settings, delays in diagnosis and treatment often lead to advanced-stage disease and poorer prognoses. From a public health perspective, eye cancer constitutes a significant yet frequently neglected factor contributing to both cancer-related mortality and vision-related disability, especially among vulnerable populations. Understanding its epidemiological patterns is crucial for informing global strategies aimed at simultaneously reducing preventable blindness and the cancer burden.

Despite the clinical significance of eye cancer, its epidemiological profile remains inadequately characterized on a global scale. The majority of existing research has concentrated on individual subtypes, such as uveal melanoma or retinoblastoma, or has been based on data derived from localized hospital registries and small cohort studies (12–14). While these studies are valuable for elucidating clinical outcomes and treatment efficacy, they provide limited insights into the population-level burden of the disease across different countries and regions (15). Notably, there has been insufficient effort to integrate the various forms of eye cancer into a comprehensive global framework that encompasses their collective incidence, mortality, and disability impact.

Furthermore, eye cancer is frequently underrepresented or minimally included in large-scale cancer surveillance initiatives, such as those conducted by international cancer registries or global oncology databases. Consequently, there is a paucity of comprehensive and comparable data regarding the temporal evolution and population-specific variations in the burden of eye cancer. This informational deficit is particularly acute in low-and middle-income countries, where factors such as underdiagnosis, incomplete cancer reporting, and limited access to specialized care exacerbate the uncertainty surrounding disease estimates. Notably, there is a dearth of studies investigating the influence of social determinants, such as sociodemographic development, sex, and age, on the distribution and outcomes of eye cancer. This lack of equity-focused research constrains policymakers’ capacity to design targeted interventions and allocate resources effectively.

To address these gaps, the present study aims to provide a comprehensive assessment of the global, regional, and national burden of eye cancer from 1990 to 2021, utilizing data from the Global Burden of Disease (GBD) Study 2021. By analyzing age-standardized incidence, prevalence, mortality, and disability-adjusted life years (DALYs) across 204 countries and territories, this research aims to quantify temporal trends and identify spatial heterogeneity in disease patterns. Beyond estimating the overall burden, we further investigate disparities stratified by sociodemographic index (SDI), sex, and age to uncover structural inequities in detection, survival, and health outcomes. Additionally, we use inequality metrics, including the concentration index (CI) and slope analysis, to evaluate the distribution of eye cancer burden along global development gradients. Through this approach, the study seeks to generate actionable evidence to guide equitable resource allocation, enhance cancer surveillance, and support the integration of eye cancer into broader global oncology and vision health strategies.

## Materials and methods

### Data source and study design

This study utilized data from the Global Burden of Disease Study 2021 (GBD 2021), coordinated by the Institute for Health Metrics and Evaluation (IHME). The GBD provides standardized, comparable estimates of disease burden across 204 countries and territories from 1990 to 2021. Data sources include national cancer registries, hospital records, vital statistics, surveys, and the published literature. All inputs were processed using consistent methods to ensure comparability across outcomes.

Eye cancers were defined according to the GBD cause hierarchy, specifically encompassing malignant neoplasms of the eye and its adnexa, such as uveal melanoma and retinoblastoma. Malignant neoplasms of the eyelid were excluded from the analysis, as these are classified as skin cancers in the GBD cause hierarchy based on the International Classification of Diseases, Tenth Revision (ICD-10) definitions. Disease burden estimates for eye cancers were reported in terms of four key metrics: incidence, prevalence, mortality, and DALYs. Each metric was calculated for both sexes and across all age groups.

All estimates were stratified by year, age, sex, country or territory, and SDI level. To facilitate cross-country comparisons and control for differences in population age structures, rates were age-standardized using the GBD world standard population. The present study adhered to the Guidelines for Accurate and Transparent Health Estimates Reporting (GATHER), ensuring methodological transparency and reproducibility (16).

### Burden estimation metrics and modeling framework

Incidence and prevalence were estimated using DisMod-MR 2.1 (17), a Bayesian meta-regression tool used in the GBD framework to synthesize data across diverse sources. Mortality estimates were generated using the Cause of Death Ensemble model (CODEm), which selects optimal predictive models based on out-of-sample performance. DALYs were calculated as the sum of years of life lost (YLLs) due to premature mortality and years lived with disability (YLDs). Disability weights (DWs) were derived from GBD population-based surveys and reflect the relative severity of health states, including moderate to severe vision impairment and cancer-related disability.

### Stratification dimensions

All estimates were stratified by sex, age, geographical location, and SDI level to enable detailed and equitable evaluation of ocular tumor burden. This stratification allowed identification of disproportionately affected subgroups and the assessment of patterns across demographic and developmental contexts.

All metrics were computed separately for males and females to explore sex-based differences in incidence, survival, and outcomes. Age-specific estimates covered 20 standard GBD age groups (ranging from 0 to 6 days to ≥95 years), enabling assessment of burden patterns across the life course.

Geographical stratification included three levels: global, 21 GBD regions, and 204 countries or territories, supporting regional and national analyses of spatial heterogeneity. Countries were also categorized into five SDI groups: low, low-middle, middle, high-middle, and high (18). The SDI is a composite index based on national income per capita, education levels (age ≥15), and fertility rates (age <25), reflecting development status and access to health resources.

### Trend and inequality analysis

To quantify long-term dynamics of the burden of eye cancer, we assessed temporal trends in age-standardized rates (ASRs) from 1990 to 2021 for incidence, prevalence, mortality, and DALYs. The direction and magnitude of these trends were evaluated using the estimated annual percentage change (EAPC). This metric was derived by fitting a linear regression model to the natural logarithm of the ASR values over time (19):


$$\ln({\mathrm{ASR}}_{t})=\alpha +\beta \times t+\varepsilon$$

where *t* represents the calendar year. The EAPC was calculated as follows:


$$\text{EAPC}=100\times (e^{\beta }-1)$$

The 95% confidence interval (CI) for the EAPC was used to determine the statistical significance of each trend: an entirely positive CI indicates a significant increase, an entirely negative CI indicates a significant decline, and a CI crossing zero suggests a stable trend.

To assess disparities in ocular tumor burden across socioeconomic development levels, we conducted inequality analyses using the SDI as a ranking variable. Two complementary metrics were applied: the CI, which measures the degree to which disease burden is distributed along the SDI continuum (positive values indicate concentration in high-SDI countries, negative values in low-SDI countries), and the Slope Index of Inequality (SII), which quantifies the absolute difference in ASRs between the most and least developed settings using linear regression. Both indices were calculated for the years 1990 and 2021 to evaluate temporal changes in equity. This methodological approach is consistent with prior GBD applications in cancer inequality research (20, 21). All analyses were stratified by sex to account for sex-specific differences in burden distribution. For visual interpretation, concentration curves and slope plots were generated for each metric. All statistical procedures were performed using R software (version 4.2.2), with dedicated packages for regression modeling and inequality analysis.

## Results

### National-level trends in ocular tumor burden (1990–2021)

From 1990 to 2021, the burden of eye cancer exhibited considerable spatial heterogeneity across 204 countries, with marked variations in trends of incidence, prevalence, mortality, and DALYs. These differences likely reflect disparities in demographic growth, healthcare infrastructure, diagnostic capacity, and treatment availability.

**Figure 1:**
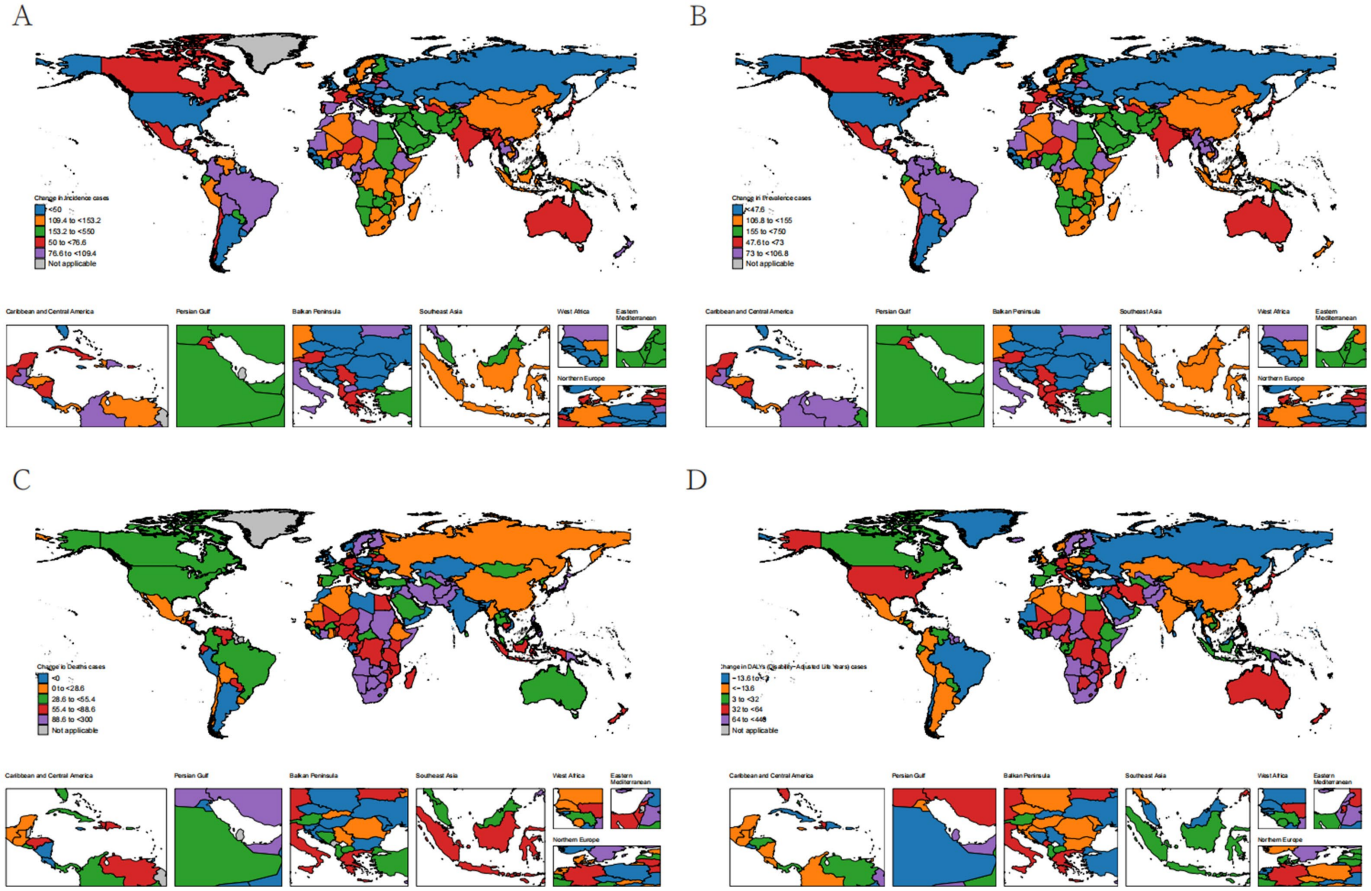
Spatial and temporal changes in incidence **(A)**, prevalence **(B)**, death **(C)**, and DALYs **(D)** of eye cancer across 204 countries from 1990 to 2021.

Incidence increased in the majority of countries, with the most pronounced relative growth observed in parts of the Middle East and East Africa. For example, the United Arab Emirates experienced a 550% increase in incident cases, with an EAPC of 2.69 (95% CI: 2.06–3.32), and Qatar showed a comparable upward trend (EAPC = 1.29; 95% CI: 0.75–1.84). In contrast, high-SDI countries with long-established cancer screening and early intervention programs—such as Austria, Japan, and France—exhibited more moderate increases (all <76.6%), suggesting relatively stable detection dynamics (Figure 1).

The prevalence increased even more sharply, particularly in low-SDI countries. Djibouti reported a 297.8% increase in prevalent cases, with an EAPC of 0.39 (95% CI: 0.31 to 0.47). Similarly, Sudan and Afghanistan experienced increases of 226.7 and 230.0%, with corresponding EAPCs of 1.76 (95% CI: 1.57 to 1.95) and 1.49 (95% CI: 1.28 to 1.69), respectively. These changes may reflect both improved survivorship and rectification of historical underreporting. In contrast, countries such as Hungary (5.9% increase) and Germany (118.3%) demonstrated relatively limited prevalence growth, indicative of plateauing disease burden in aging but well-managed populations.

In terms of mortality, high-SDI countries achieved significant reductions in age-standardized death rates (ASDRs) related to ocular tumors. For example, Australia reported a substantial decline (EAPC = −1.53; 95% CI: −1.66 to −1.39), while Japan showed a nearly stable trend (EAPC = −0.05; 95% CI: −0.34 to 0.24). Interestingly, Switzerland experienced a modest increase in ASDRs (EAPC = 0.78; 95% CI: 0.57 to 1.00) despite its advanced healthcare infrastructure. These trends underscore the benefits of sustained investment in early detection and effective treatment services. In contrast, Southern sub-Saharan Africa witnessed a 142.9% increase in the absolute number of deaths, reflecting continued challenges such as limited access to oncology care and delayed diagnosis.

With regard to DALYs, several regions demonstrated notable progress in reducing the overall burden of eye cancer. Southern Latin America achieved the greatest decline in DALY ASR, decreasing from 3.753 (95% UI: 3.099–4.616) in 1990 to 1.813 (95% UI: 1.551–2.177) in 2021, with an EAPC of −2.06 (95% CI: −2.28 to −1.84). East Asia and Central Europe followed similar downward trajectories, with DALY ASR reductions from 3.625 to 1.701 (EAPC = −1.79) and from 7.021 to 4.240 (EAPC = −1.75), respectively. In contrast, Nigeria and Yemen showed marked increases in total DALYs—93.8 and 100%, respectively—highlighting the ongoing burden in settings with persistent diagnostic delays and treatment gaps.

### Sociodemographic inequality in ocular tumor burden (1990 vs. 2021)

Over the past three decades, sociodemographic disparities in ocular tumor burden have persisted across all four major indicators—prevalence, incidence, mortality, and DALYs. Using CI and slope index analyses, we assessed the magnitude and direction of inequality by national SDI rank between 1990 and 2021.

**Figure 2:**
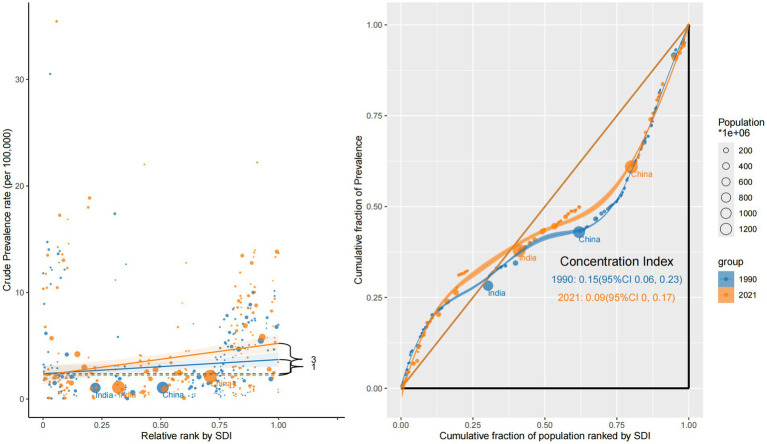
Sociodemographic inequality in prevalence (1990 vs. 2021).

The prevalence remained disproportionately concentrated in high-SDI countries. Although the inequality gap narrowed modestly—from a CI of +0.15 (95% CI: 0.06 to 0.23) in 1990 to +0.09 (95% CI: 0.00 to 0.17) in 2021—the burden of ocular tumor prevalence continued to favor more developed settings. This trend is likely driven by improved survivorship in high-SDI regions, where early detection and prolonged care result in cumulative prevalence. In contrast, low-SDI countries displayed flatter slope gradients, consistent with underdiagnosis, limited survival, and weak surveillance systems (Figure 2).

Incidence also exhibited inequality, favoring high-SDI nations. The CI declined from +0.16 (95% CI: 0.07 to 0.24) in 1990 to +0.10 (95% CI: 0.02 to 0.19) in 2021, indicating a narrowing but persistent concentration of new cases in wealthier countries. Slope curves revealed steeper increases in incidence rates among upper-middle and high-SDI countries, suggesting the influence of stronger diagnostic capacity and national screening initiatives (Supplementary Figure S1).

**Figure 3:**
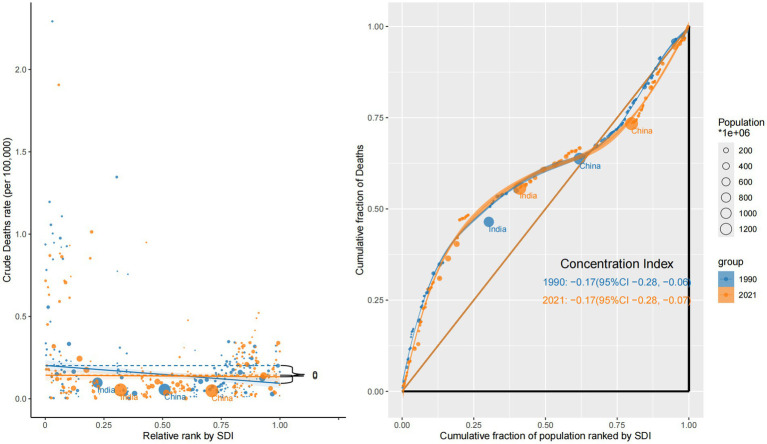
Sociodemographic inequality in mortality (1990 vs. 2021).

In contrast, mortality showed a persistent inverse inequality pattern. The CI was negative in both years—–0.17 (95% CI: −0.28 to −0.06) in 1990 and −0.17 (95% CI: −0.28 to −0.07) in 2021—highlighting a disproportionate mortality burden in low-SDI countries. This reflects delayed diagnosis, poor access to treatment, and inadequate oncology infrastructure. Steep negative slopes were particularly evident in sub-Saharan Africa and South Asia, reinforcing regional disparities in eye cancer lethality (Figure 3).

DALY burden was similarly skewed toward low-SDI regions and exhibited the most pronounced inequality. The CI declined further from −0.34 (95% CI: −0.45 to −0.22) in 1990 to −0.38 (95% CI: −0.50 to −0.26) in 2021, with steep slope gradients indicating the continued concentration of YLLs and YLDs in the world’s poorest populations (Supplementary Figure S2).

Collectively, these findings demonstrate a dual inequality structure: high-SDI countries experience higher detection and longer survivorship (reflected in elevated prevalence and incidence), whereas low-SDI countries carry a disproportionate burden of mortality and disability. The lack of meaningful convergence—particularly for DALYs and deaths—underscores the urgent need for more equitable access to early diagnosis, timely treatment, and survivorship care across socioeconomically disadvantaged regions.

### Age and SDI-specific changes in prevalence patterns (1990 vs. 2021)

**Figure 4:**
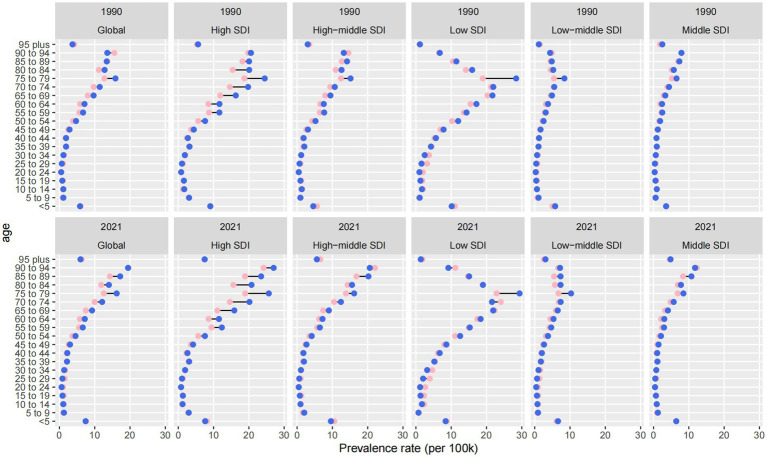
Age- and SDI-specific changes in age-standardized prevalence rates of eye cancer from 1990 to 2021.

Between 1990 and 2021, global patterns in age-specific prevalence rates (ASPRs) of ocular tumors revealed a marked rightward shift, with prevalence increasingly concentrated in older populations and strongly influenced by sociodemographic development (Figure 4). This shift reflects both global aging and disparities in healthcare access and survivorship.

Across all regions, ASPRs increased in nearly every age group, but the steepest growth occurred among individuals aged ≥65, particularly those ≥75. This trend highlights a growing concentration of disease burden in the oldest cohorts, especially in high-SDI countries. While overall ASPRs in these settings remained relatively stable (4.976 in 1990 vs. 4.845 in 2021), the prevalence among those ≥75 years increased disproportionately, especially in male patients. This reflects improved longevity after diagnosis and accumulation of age-related risk, despite no corresponding rise in incidence.

In middle-and low-middle SDI regions, the most rapid ASPR increases were observed in the 45–64 age group. Middle-SDI countries experienced the fastest ASPR growth globally, with an EAPC of +1.39 (95% CI: 1.28 to 1.51) and an increase from 1.505 to 2.048 per 100,000. Similarly, low-and middle-SDI countries saw a 117.1% increase in prevalence numbers. These patterns may indicate earlier disease onset or increased midlife survivorship due to modest improvements in detection and primary treatment, though without sustained long-term care for older adults.

In contrast, low-SDI countries consistently bore the highest absolute ASPRs across all age groups. The total ASPRs increased from 6.540 to 6.969 per 100,000, with a 118.9% increase in case numbers from 1990 to 2021. Growth was most pronounced among adults over 60, but younger age groups also showed moderate increases—likely driven by delayed diagnosis, minimal access to curative therapies, and limited palliative infrastructure. The persistently high prevalence in these settings reflects both the accumulation of untreated disease and stagnation in care delivery systems.

Sex-based disparities were evident across all SDI levels and age groups. Males consistently exhibited higher ASPRs than females, and this gap widened with age. In high-SDI regions, this divergence was particularly pronounced among individuals ≥75, possibly reflecting longer male survival or greater cumulative exposure to occupational and environmental risk factors. Globally, male ASPRs increased from 3.274 to 3.584 per 100,000, compared to an increase from 3.018 to 3.354 per 100,000 in female ASPRs.

### Sex-based inequality in 2021 across all metrics

In 2021, ocular tumors demonstrated marked sex disparities across all four major burden metrics—prevalence, incidence, mortality, and DALYs—shaped by the intersecting influences of sex, socioeconomic development, and healthcare system performance. The extent and direction of inequality varied across indicators and SDI levels, underscoring the complexity of sex-specific vulnerabilities in global ocular oncology.

**Figure 5:**
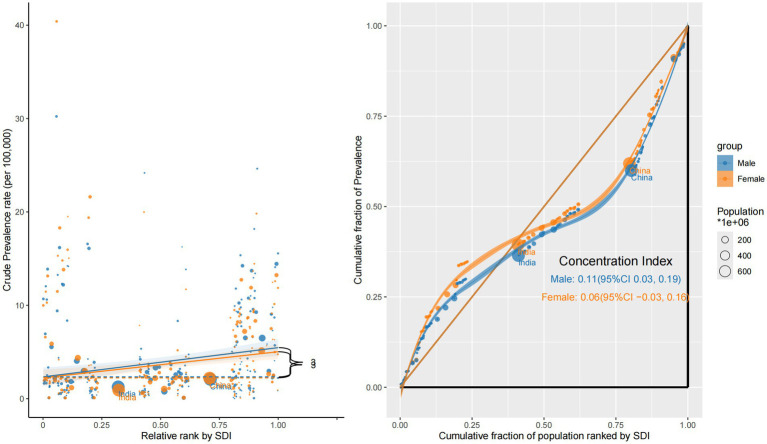
Sex-specific concentration curves and slope index plots for the prevalence of eye cancer in 2021.

Prevalence disparities were the most pronounced. The CI for males was +0.11 (95% CI: 0.03 to 0.19), suggesting a statistically significant concentration of ocular tumor prevalence in high-SDI countries. In contrast, the CI for females was +0.06 (95% CI: −0.03 to 0.16), a non-significant result that indicates no clear inequality. Male prevalence curves exhibited a distinct concave shape below the equality line, while the female curve approximated the diagonal, signaling a flatter distribution. Slope analysis confirmed this pattern: male ASPRs increased steadily with SDI, whereas female ASPRs remained relatively stable. These trends likely reflect enhanced diagnostic capacity and longer post-diagnosis survival among male participants in affluent regions, and potentially underdiagnosis or reduced healthcare access for women in low-SDI countries (Figure 5).

Incidence inequality followed a similar but less pronounced pattern. The CI was +0.12 (95% CI: 0.04 to 0.21) for male and +0.08 (95% CI: −0.01 to 0.18) for female participants, indicating a modest concentration of new cases in high-SDI settings. The male incidence distribution deviated more prominently from the equality line, and slope gradients were steeper, implying greater detection or risk exposure. In contrast, female incidence rates were more evenly spread across SDI levels, potentially due to diagnostic gaps or delayed care in resource-limited regions (Supplementary Figure S3).

**Figure 6:**
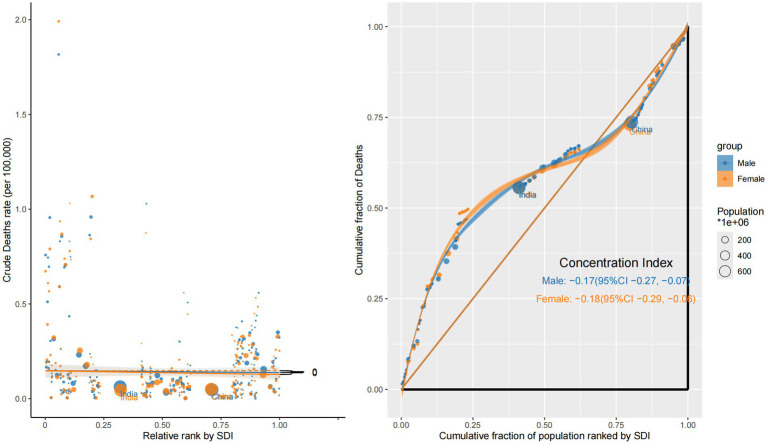
Sex-specific concentration curves and slope index plots for the mortality of eye cancer in 2021.

Mortality burden revealed a reversed and more severe pattern of inequality. Both sexes showed negative concentration indices—–0.17 (95% CI: −0.27 to −0.07) for males and −0.18 (95% CI: −0.29 to −0.06) for females—indicating that deaths due to ocular tumors were disproportionately concentrated in low-SDI countries. The corresponding concentration curves were convex and situated above the equality line, with slope plots showing sharp negative gradients, particularly for females. These findings likely stem from late-stage diagnoses, inadequate treatment infrastructure, and insufficient follow-up care in low-income settings, especially for women (Figure 6).

DALY inequality was the most extreme across all metrics and both sexes. The CI reached −0.36 (95% CI: −0.48 to −0.25) for males and −0.39 (95% CI: −0.51 to −0.27) for females, highlighting that ocular tumor–related health loss—encompassing both premature mortality and YLDs—was overwhelmingly concentrated in underdeveloped regions. The concentration curves deviated substantially above the equality line, and slope plots showed steep negative gradients, particularly in sub-Saharan Africa and South Asia. These patterns indicate a compounded disadvantage for populations in low-SDI settings, where both men and women endure greater health loss due to systemic barriers to early detection, effective treatment, and long-term survivorship support (Supplementary Figure S4).

## Discussion

This study provides novel insights into the global landscape of eye cancer by unveiling the structural patterns of inequality embedded within its epidemiology. By leveraging standardized and comparable data from the GBD 2021 framework, we identified not only quantitative shifts in incidence, prevalence, mortality, and DALYs over time, but also deeply entrenched disparities across countries, age groups, and sexes. These findings underscore the reality that improvements in survival and detection in high-SDI settings are not paralleled by corresponding progress in low-resource environments. Instead, disease burden in many low-and middle-income countries remains exacerbated by systemic gaps in early diagnosis, treatment infrastructure, and survivorship care.

Despite the clinical significance of eye cancers, they have been largely neglected in global burden assessments (22, 23). Prior studies have predominantly relied on data from hospital-based cohorts or regional cancer registries, often emphasizing histopathological characteristics, treatment outcomes, or survival rates within specific populations (24–28). In contrast, our study comprehensively examines ocular malignancies at a global population level, integrating multiple tumor subtypes and stratifying burden by age, sex, and SDI quintile. Importantly, we reveal previously undocumented epidemiological patterns—including a disproportionate concentration of prevalence in high-SDI countries due to aging populations and enhanced detection, and persistent excess mortality and DALY burdens in low-SDI regions where access to timely care remains limited. Sex-specific disparities are also evident, with elderly males in high-income settings exhibiting rising incidence, while women in low-SDI settings disproportionately shoulder the untreated disease burden due to structural and social barriers. These findings elevate eye cancers from a niche clinical concern to a broader public health priority and demonstrate how their burden is shaped not only by biology, but by development, equity, and systems capacity. Our analysis thus fills a critical knowledge gap by providing a longitudinal and stratified view of eye cancer burden that can guide future research and inform context-sensitive interventions. It also highlights the urgent need to embed ophthalmic oncology more explicitly within global cancer control and vision health agendas.

In comparison to other solid tumor GBD analyses, such as those concerning central nervous system neoplasms (29), oral cavity cancers (30), or cutaneous melanoma (31), eye cancers present several distinct epidemiological characteristics. For instance, unlike brain tumors (32), where mortality trends have stabilized across most high-income regions, eye cancers exhibit continuous declines in DALYs in these countries. This trend is likely attributable to advances in early detection and organ-preserving treatment modalities. In contrast, while skin cancers are predominantly influenced by ultraviolet exposure and are more prevalent in high-latitude countries (33), eye cancers display greater geographic heterogeneity, with rapidly increasing incidence rates observed in parts of the Middle East and East Africa. These distinctions indicate that eye cancers occupy a unique position within the global cancer burden landscape, shaped not only by anatomical susceptibility and environmental risk factors but also by significant deficiencies in surveillance, diagnostic, and treatment infrastructures.

Sex-specific patterns have revealed significant disparities in the burden of eye cancers. Males consistently demonstrate higher age-standardized mortality and DALY rates compared to females across most regions and age groups. These disparities may be attributed to a combination of factors, including delayed healthcare engagement, greater occupational or environmental exposures, and potential biological differences in tumor aggressiveness or immune response. The disadvantage in survival outcomes among males necessitates targeted intervention strategies to enhance early detection and treatment adherence, particularly in regions with a high disease burden (34, 35). However, when stratified by the development level, the inequality in mortality and DALY burden is more severe among women in low-SDI countries, as reflected by more negative CI values. Beyond biological and healthcare system factors, these disparities are likely exacerbated by entrenched social determinants. In many low-resource settings, women face compounded barriers to accessing timely and effective care, including limited autonomy in health decision-making, lower health literacy, and economic dependency on males in the family (36). Additionally, sociocultural constraints, such as stigma surrounding cancer diagnosis or restrictions on women’s mobility, can result in delayed care-seeking and advanced-stage presentation. These systemic inequalities may contribute to underdiagnosis, treatment discontinuation, and suboptimal follow-up, ultimately amplifying the burden of disease among women in disadvantaged populations. Therefore, addressing both the higher absolute burden in men and the disproportionate relative burden in disadvantaged women is essential for developing equitable, sex-responsive strategies in global eye cancer control.

The observed disparities in eye cancer burden across SDI levels are attributed to structural differences in healthcare systems, access to diagnostics, and demographic transitions. In high-SDI countries, declining mortality and DALY rates reflect the benefits of early detection through routine screening, advanced treatment options such as brachytherapy and proton beam therapy, and comprehensive survivorship care. These countries also face a growing prevalence burden due to improved survival and rapid population aging, with diseases increasingly concentrated among elderly males. In contrast, low-SDI regions continue to bear a disproportionate mortality and disability burden, largely driven by delayed diagnoses, limited treatment availability, and under-resourced healthcare infrastructure. Additionally, rising prevalence among middle-aged populations in these settings may indicate earlier onset or prolonged survival without adequate follow-up. These inequities highlight the need for targeted global strategies to promote early diagnosis, strengthen care delivery systems, and address the growing impact of demographic shifts.

The heterogeneous distribution of ocular tumor burden across various regions, age groups, and sociodemographic strata necessitates the implementation of stratified, context-sensitive interventions rather than uniform solutions. First, the increasing incidence and persistently high burden of eye cancers in pediatric populations, particularly in regions with low SDI, underscore the urgent need to enhance early screening programs for childhood eye cancers, such as retinoblastoma. Integrating ocular screening into routine pediatric health visits, alongside improved community-level education to raise awareness of early warning signs, may help reduce diagnostic delays. Establishing and expanding population-based ocular tumor registries is also crucial for improving epidemiologic surveillance and guiding resource allocation in both low-and middle-income countries (27, 37). Second, in high-burden settings, such as Eastern sub-Saharan Africa, a paradigm shift is necessary to develop sustainable, low-cost ocular oncology care platforms. Task-shifting strategies, such as training non-specialist providers to recognize ocular malignancies, increasing telemedicine capacity, and decentralizing care delivery, may address the shortage of specialists. Furthermore, governmental investment in fundamental treatment infrastructure, such as radiotherapy units, chemotherapy supply chains, and surgical training programs, has the potential to significantly decrease mortality and disability associated with eye cancers (38). As survivorship rates improve in many high-and middle-SDI regions, particularly among the elderly, it is imperative to shift focus toward the functional and psychosocial dimensions of care. This includes integrating vision preservation, rehabilitation services, assistive technologies (e.g., low vision aids and mobility training), and long-term psychosocial support into national cancer control plans. Neglecting to address post-treatment quality of life could lead to an underestimation of the broader societal and economic impacts of eye cancers, especially given the rising prevalence. Specific strategies may include routine ocular oncology screenings for high-risk elderly populations, geriatric-specific treatment protocols that consider comorbidities and functional reserve, and coordinated care models that involve ophthalmologists, oncologists, and primary care providers to facilitate shared decision-making and ongoing monitoring. Finally, the marked inequalities in burden and trends across SDI levels underscore the need for global cooperation. Our findings align with the priorities outlined in the WHO’s Universal Eye Health: A Global Action Plan 2014–2019, which emphasizes the need to strengthen eye health systems, improve access to care, and reduce avoidable vision impairment through early detection and treatment. Multilateral funding agencies, non-governmental organizations, and international cancer alliances should prioritize ophthalmic oncology capacity-building in under-resourced countries. Sharing of best practices, treatment protocols, and surveillance infrastructure through international partnerships can accelerate progress toward equity in ocular cancer prevention and care.

Several limitations inherent to the GBD methodology and data inputs should be considered when interpreting our findings. First, despite the GBD framework’s integration of diverse data sources, such as cancer registries and hospital records, the availability and quality of ocular tumor data exhibit considerable variability across different countries. In numerous low-income regions, issues such as underreporting, misclassification, and inadequate cancer surveillance infrastructure may introduce uncertainty or systematic bias into the estimates (28, 39). Second, the disease category examined in this study includes a heterogeneous array of malignancies affecting various ocular structures, each with distinct biological behaviors, prognoses, and treatment pathways. The inability to differentiate among these subtypes restricts the granularity of epidemiological interpretation and may obscure significant subtype-specific patterns. Notably, malignant tumors of the eyelid were excluded from our analysis, as they are classified under malignant neoplasms of the skin in the ICD-10 framework used by GBD 2021. This exclusion may lead to an underestimation of the total ocular cancer burden. Third, although ASRs serve as a valuable tool for comparison, they fail to capture the full extent of the functional burden, such as the severity of vision loss, impairments in quality of life, or psychosocial impacts, which are particularly pertinent in the context of ocular oncology. Similarly, the use of modeled DALY estimates may not fully reflect context-specific access to rehabilitation or survivorship support, especially in resource-limited settings.

Nevertheless, despite these limitations, by systematically stratifying burden estimates according to age, sex, and the SDI level, this study presents the most comprehensive analysis to date of global disparities in ocular oncology. The findings highlight that eye cancers, although relatively rare, mirror broader patterns of structural health inequities and resource imbalances within global cancer control efforts. Consequently, the study underscores the pressing need for targeted strategies that extend beyond clinical interventions. These strategies should focus on enhancing early detection, improving treatment capacity in underserved regions, and ensuring equitable survivorship care throughout the lifespan.

## Conclusion

This research advances the epidemiological understanding of eye cancers and lays the groundwork for integrating these malignancies into national and international health planning. Furthermore, it offers a replicable framework for equity-oriented analysis of other rare but impactful diseases, reinforcing the significance of inclusive, data-driven approaches in global health policy and oncology research.

## Data Availability

The original contributions presented in the study are included in the article/Supplementary material, further inquiries can be directed to the corresponding authors.

## Supplementary material

SUPPLEMENTARY FIGURE S1 Socio-demographic inequality in incidence (1990 vs. 2021).

SUPPLEMENTARY FIGURE S2 Socio-demographic inequality in DALYs (1990 vs. 2021).

SUPPLEMENTARY FIGURE S3 Gender-specific concentration curves and slope index plots for the incidence of eye cancer in 2021.

SUPPLEMENTARY FIGURE S4 Gender-specific concentration curves and slope index plots for the DALY burden of eye cancer in 2021.
