# Supplementary figures and images for: Global trends and inequalities in eye cancer burden: a comprehensive analysis based on the global burden of disease study
Source: Front Med (Lausanne). 2025 Aug 14;12:1638733. doi: 10.3389/fmed.2025.1638733 (PMC12391050; doi:10.3389/fmed.2025.1638733)

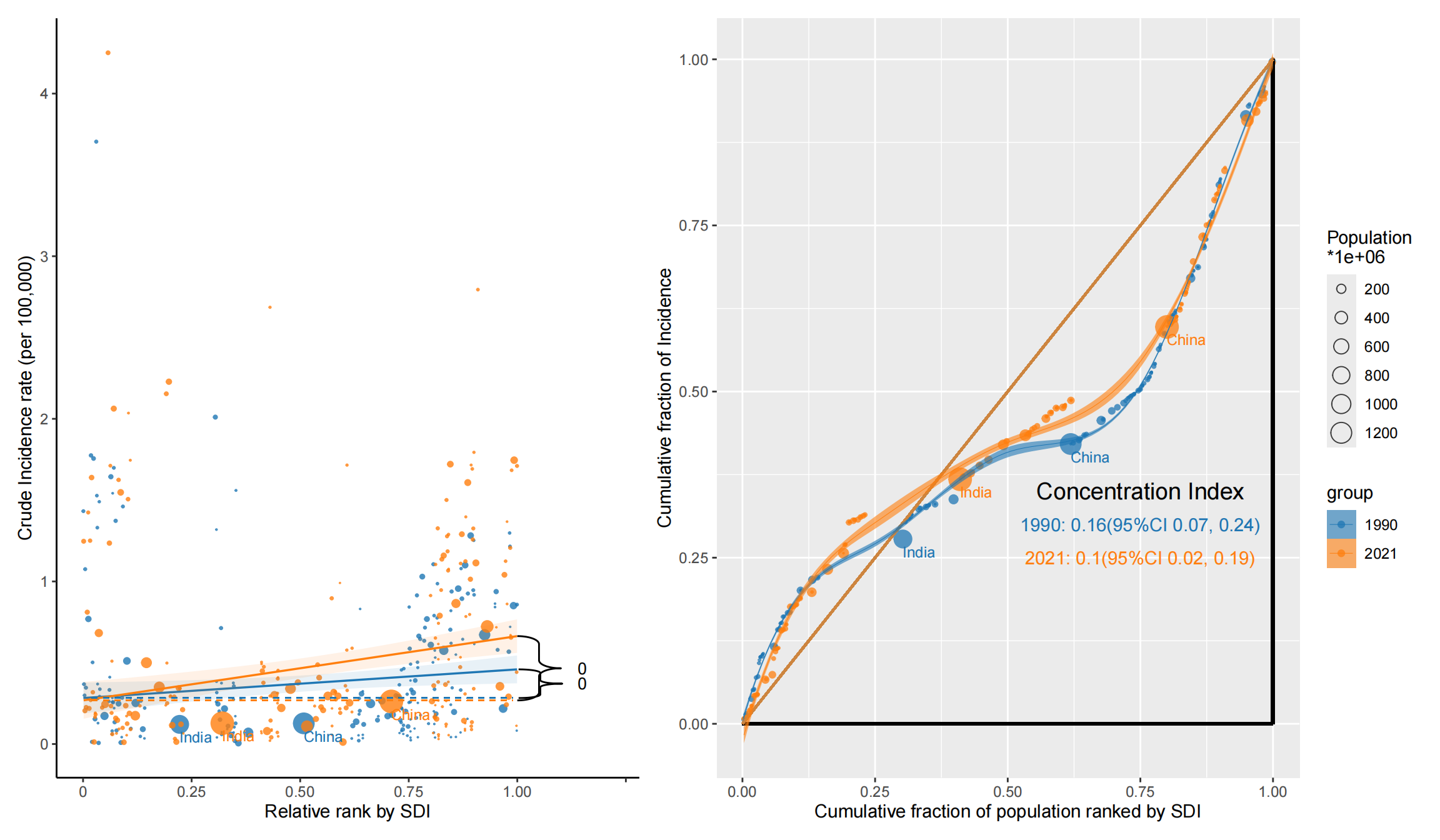

Supplement: SUPPLEMENTARY FIGURE S1 — Socio-demographic inequality in incidence (1990 vs. 2021). [file Image_1.tif]

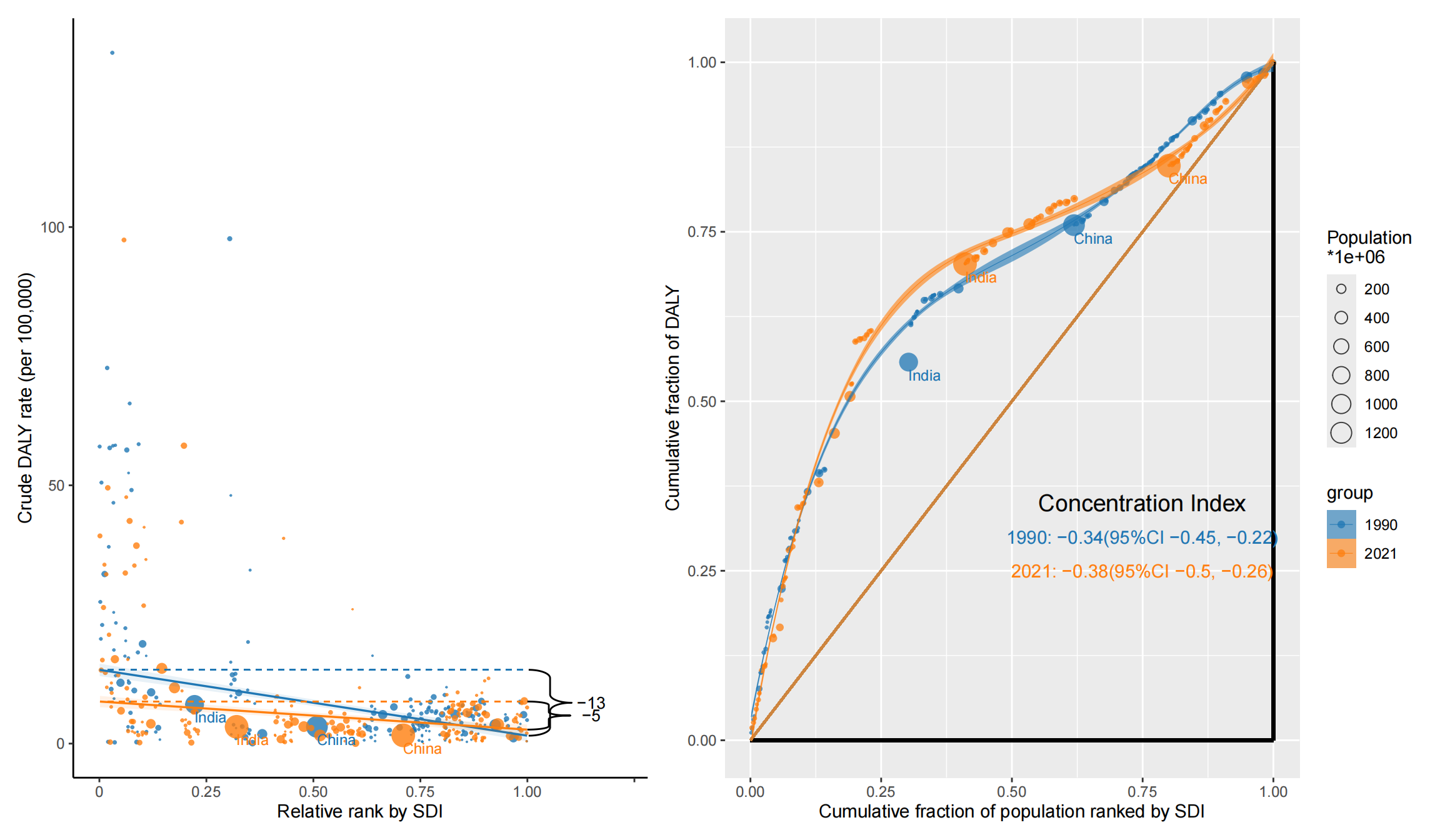

Supplement: SUPPLEMENTARY FIGURE S2 — Socio-demographic inequality in DALYs (1990 vs. 2021). [file Image_2.tif]

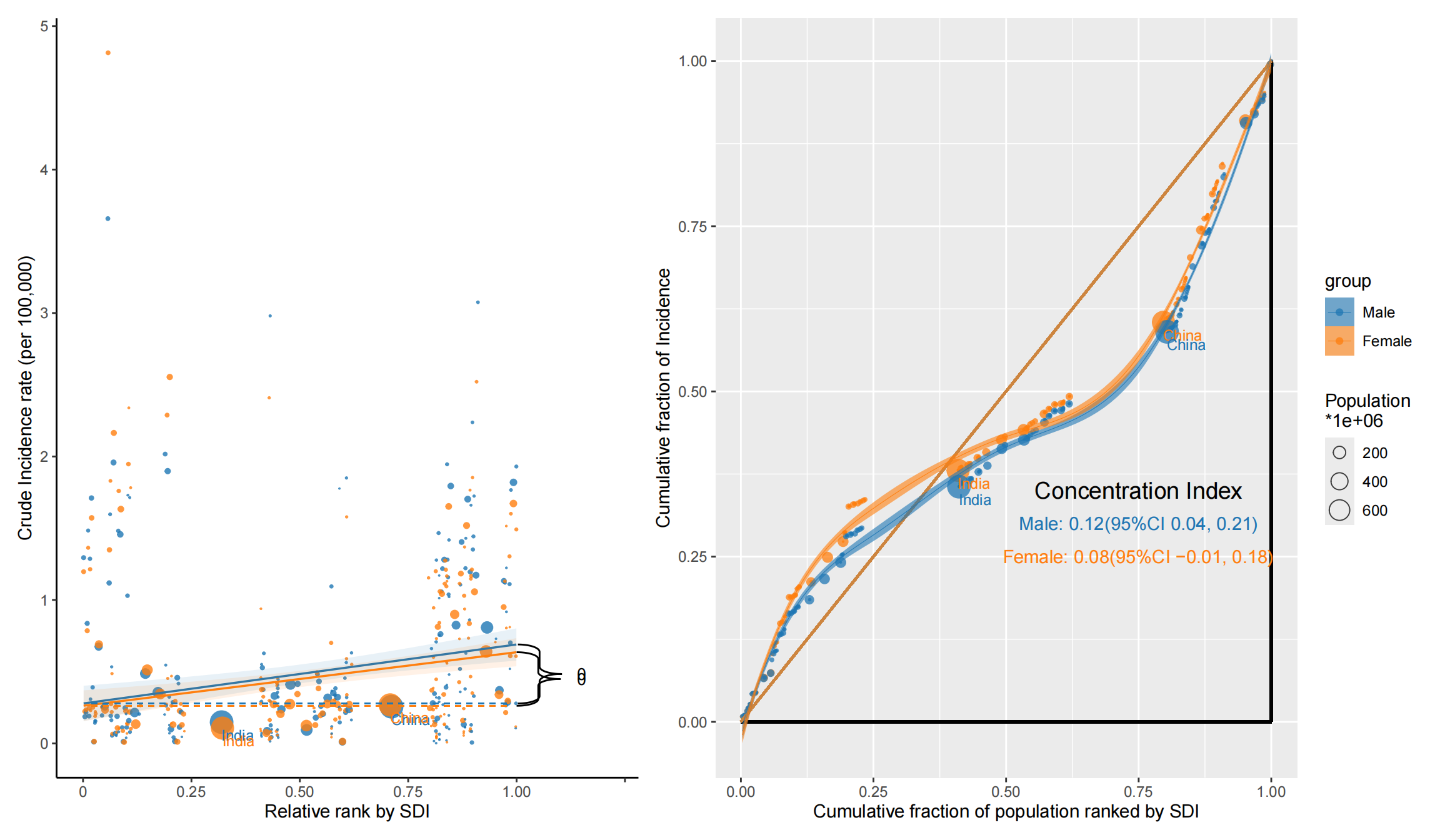

Supplement: SUPPLEMENTARY FIGURE S3 — Gender-specific concentration curves and slope index plots for the incidence of eye cancer in 2021. [file Image_3.tif]

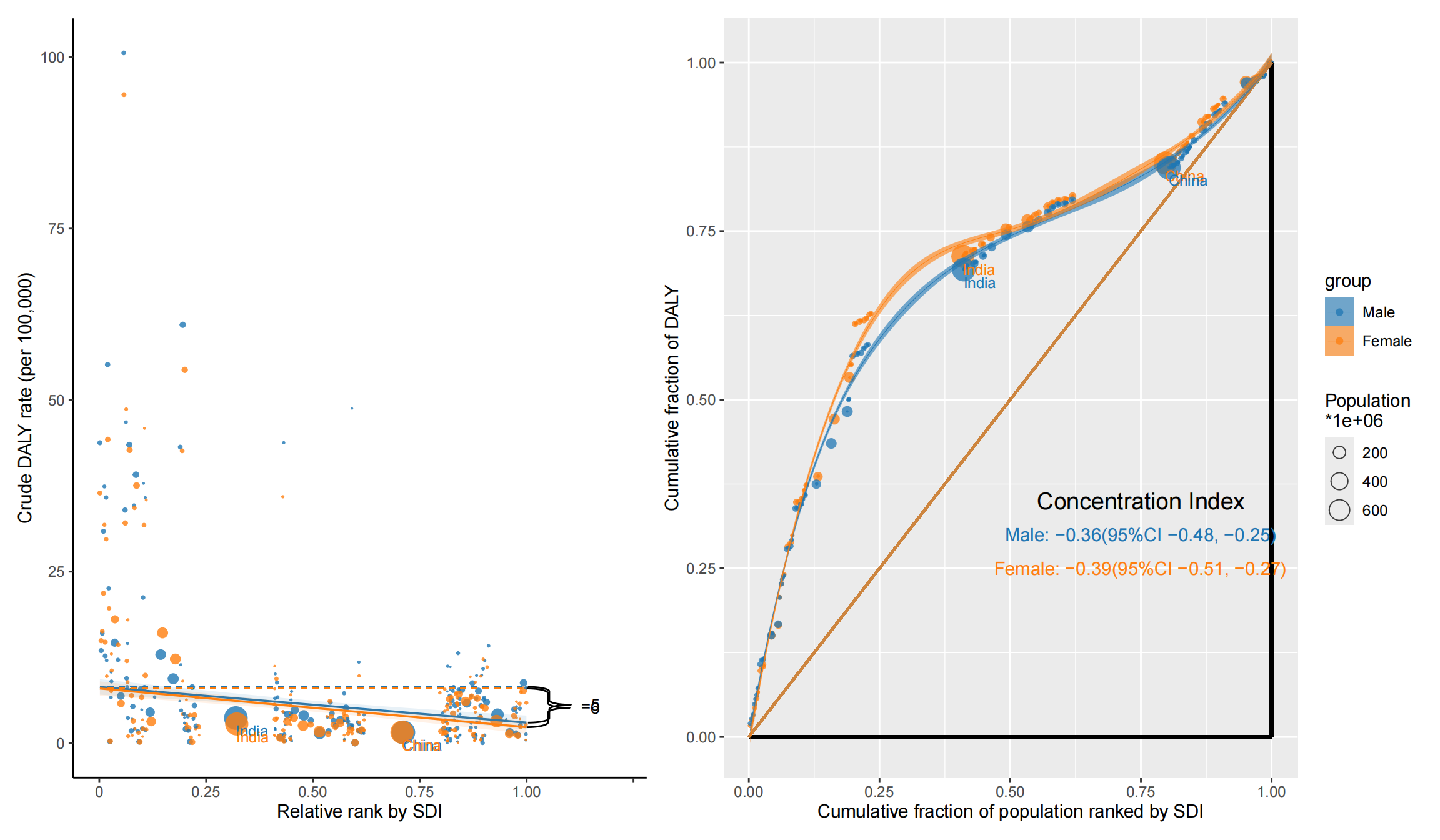

Supplement: SUPPLEMENTARY FIGURE S4 — Gender-specific concentration curves and slope index plots for the DALY burden of eye cancer in 2021. [file Image_4.tif]
