# Supplementary material for: Global trends and inequalities in eye cancer burden: a comprehensive analysis based on the global burden of disease study
Source: Front Med (Lausanne). 2025 Aug 14;12:1638733. doi: 10.3389/fmed.2025.1638733 (PMC12391050; doi:10.3389/fmed.2025.1638733)
Supplement: Supplementary file 5 [file Table_1.docx]

**Supplementary Table S1: Prevalence of Eye Cancer in 204 Countries and Territories in 1990 and 2021, with EAPC**

| **Location** | **1990** | | |  | **2021** | | | **EAPC(95%CI)** |
| --- | --- | --- | --- | --- | --- | --- | --- | --- |
|  | **Number(95%UI)** | **ASR(95%UI)** | **Rate(95%UI)** |  | **Number(95%UI)** | **ASR(95%UI)** | **Rate(95%UI)** |  |
| Afghanistan | 10 ( 1 to 44 ) | 0.124 ( 0.014 to 0.528 ) | 0.102 ( 0.013 to 0.441 ) |  | 33 ( 5 to 129 ) | 0.183 ( 0.029 to 0.721 ) | 0.104 ( 0.017 to 0.412 ) | 1.49 (1.28 to 1.69) |
| Albania | 223 ( 147 to 321 ) | 9.035 ( 5.954 to 13.064 ) | 6.757 ( 4.452 to 9.7 ) |  | 341 ( 228 to 543 ) | 8.733 ( 5.801 to 13.782 ) | 12.786 ( 8.555 to 20.335 ) | 0.24 (0.04 to 0.44) |
| Algeria | 246 ( 156 to 373 ) | 1.12 ( 0.711 to 1.596 ) | 0.972 ( 0.615 to 1.477 ) |  | 581 ( 384 to 852 ) | 1.404 ( 0.922 to 2.037 ) | 1.315 ( 0.868 to 1.927 ) | 0.71 (0.58 to 0.84) |
| American Samoa | 0 ( 0 to 0 ) | 0.492 ( 0.282 to 0.757 ) | 0.359 ( 0.192 to 0.596 ) |  | 0 ( 0 to 0 ) | 0.607 ( 0.337 to 0.945 ) | 0.57 ( 0.316 to 0.864 ) | 0.95 (0.44 to 1.46) |
| Andorra | 3 ( 2 to 4 ) | 4.847 ( 2.823 to 7.353 ) | 5.103 ( 2.929 to 7.902 ) |  | 5 ( 3 to 8 ) | 3.718 ( 2.119 to 5.585 ) | 5.771 ( 3.166 to 8.886 ) | -0.61 (-0.83 to -0.38) |
| Angola | 221 ( 133 to 366 ) | 2.623 ( 1.58 to 4.628 ) | 2.155 ( 1.292 to 3.564 ) |  | 655 ( 385 to 1108 ) | 2.92 ( 1.682 to 5.469 ) | 2.002 ( 1.176 to 3.388 ) | 0.35 (0.2 to 0.51) |
| Antigua and Barbuda | 1 ( 1 to 1 ) | 2.097 ( 1.649 to 2.614 ) | 1.961 ( 1.537 to 2.469 ) |  | 4 ( 3 to 4 ) | 3.656 ( 3.064 to 4.282 ) | 4.075 ( 3.457 to 4.752 ) | 2.36 (1.52 to 3.21) |
| Argentina | 861 ( 634 to 1154 ) | 2.6 ( 1.923 to 3.465 ) | 2.6 ( 1.915 to 3.486 ) |  | 942 ( 763 to 1174 ) | 1.949 ( 1.56 to 2.463 ) | 2.071 ( 1.677 to 2.582 ) | -0.5 (-0.83 to -0.16) |
| Armenia | 85 ( 52 to 128 ) | 2.809 ( 1.737 to 4.187 ) | 2.481 ( 1.525 to 3.734 ) |  | 135 ( 85 to 204 ) | 3.687 ( 2.26 to 5.869 ) | 4.499 ( 2.845 to 6.825 ) | 1.15 (0.96 to 1.35) |
| Australia | 1214 ( 1077 to 1372 ) | 6.52 ( 5.802 to 7.364 ) | 7.199 ( 6.39 to 8.14 ) |  | 2091 ( 1630 to 2625 ) | 5.47 ( 4.325 to 6.93 ) | 8.107 ( 6.319 to 10.179 ) | -0.54 (-0.73 to -0.35) |
| Austria | 587 ( 510 to 666 ) | 6.41 ( 5.547 to 7.35 ) | 7.553 ( 6.562 to 8.577 ) |  | 945 ( 762 to 1160 ) | 7.053 ( 5.683 to 8.687 ) | 10.516 ( 8.488 to 12.915 ) | 1 (0.73 to 1.27) |
| Azerbaijan | 100 ( 53 to 188 ) | 1.472 ( 0.841 to 2.626 ) | 1.36 ( 0.719 to 2.573 ) |  | 169 ( 83 to 342 ) | 1.782 ( 0.839 to 3.719 ) | 1.614 ( 0.786 to 3.253 ) | 0.85 (0.72 to 0.99) |
| Bahamas | 12 ( 9 to 17 ) | 5.141 ( 3.934 to 6.744 ) | 4.722 ( 3.495 to 6.516 ) |  | 17 ( 12 to 22 ) | 4.168 ( 3.063 to 5.463 ) | 4.274 ( 3.139 to 5.609 ) | -0.54 (-0.97 to -0.12) |
| Bahrain | 1 ( 1 to 2 ) | 0.329 ( 0.216 to 0.637 ) | 0.197 ( 0.127 to 0.364 ) |  | 8 ( 3 to 13 ) | 0.694 ( 0.268 to 1.129 ) | 0.494 ( 0.188 to 0.836 ) | 3.46 (3.04 to 3.88) |
| Bangladesh | 1647 ( 906 to 2903 ) | 1.34 ( 0.777 to 2.267 ) | 1.51 ( 0.83 to 2.661 ) |  | 2482 ( 1431 to 4565 ) | 1.672 ( 0.961 to 3.06 ) | 1.507 ( 0.869 to 2.773 ) | 0.84 (0.63 to 1.06) |
| Barbados | 4 ( 3 to 6 ) | 1.744 ( 1.205 to 2.819 ) | 1.656 ( 1.203 to 2.48 ) |  | 6 ( 4 to 10 ) | 2.733 ( 1.471 to 4.822 ) | 2.154 ( 1.448 to 3.226 ) | 2.32 (1.73 to 2.91) |
| Belarus | 542 ( 431 to 688 ) | 4.53 ( 3.664 to 5.736 ) | 5.188 ( 4.124 to 6.59 ) |  | 1106 ( 829 to 1495 ) | 8.856 ( 6.458 to 12.389 ) | 11.866 ( 8.89 to 16.036 ) | 2 (1.75 to 2.25) |
| Belgium | 736 ( 604 to 875 ) | 6.154 ( 5.075 to 7.294 ) | 7.378 ( 6.05 to 8.768 ) |  | 1148 ( 903 to 1429 ) | 7.007 ( 5.472 to 8.604 ) | 10.008 ( 7.877 to 12.459 ) | 0.7 (0.4 to 1) |
| Belize | 1 ( 1 to 2 ) | 0.477 ( 0.289 to 0.857 ) | 0.639 ( 0.343 to 1.248 ) |  | 1 ( 1 to 1 ) | 0.2 ( 0.152 to 0.274 ) | 0.178 ( 0.135 to 0.243 ) | -2.38 (-3.46 to -1.28) |
| Benin | 142 ( 71 to 284 ) | 2.482 ( 1.478 to 4.145 ) | 2.932 ( 1.471 to 5.847 ) |  | 265 ( 126 to 554 ) | 1.909 ( 1.086 to 3.207 ) | 1.965 ( 0.934 to 4.102 ) | -0.95 (-1.16 to -0.74) |
| Bermuda | 0 ( 0 to 0 ) | 0.332 ( 0.259 to 0.429 ) | 0.337 ( 0.269 to 0.427 ) |  | 0 ( 0 to 1 ) | 0.398 ( 0.303 to 0.533 ) | 0.679 ( 0.52 to 0.902 ) | 0.49 (0.11 to 0.88) |
| Bhutan | 7 ( 3 to 15 ) | 1.124 ( 0.62 to 1.984 ) | 1.134 ( 0.542 to 2.366 ) |  | 11 ( 6 to 21 ) | 1.76 ( 0.896 to 3.338 ) | 1.484 ( 0.759 to 2.807 ) | 1.55 (1.38 to 1.73) |
| Bolivia (Plurinational State of) | 194 ( 121 to 335 ) | 3.064 ( 1.977 to 4.755 ) | 3.038 ( 1.89 to 5.246 ) |  | 369 ( 234 to 587 ) | 3.471 ( 2.234 to 5.404 ) | 3.124 ( 1.982 to 4.976 ) | 0.42 (0.28 to 0.57) |
| Bosnia and Herzegovina | 167 ( 116 to 240 ) | 3.911 ( 2.737 to 5.56 ) | 3.702 ( 2.576 to 5.326 ) |  | 228 ( 155 to 313 ) | 4.139 ( 2.789 to 5.666 ) | 6.895 ( 4.708 to 9.467 ) | 0.5 (0.36 to 0.64) |
| Botswana | 41 ( 22 to 86 ) | 4.426 ( 2.315 to 9.746 ) | 3.116 ( 1.693 to 6.494 ) |  | 88 ( 45 to 178 ) | 4.445 ( 2.307 to 9.091 ) | 3.691 ( 1.863 to 7.447 ) | 0.2 (-0.03 to 0.44) |
| Brazil | 3096 ( 2455 to 3884 ) | 2.502 ( 1.968 to 3.141 ) | 2.085 ( 1.653 to 2.615 ) |  | 6064 ( 5153 to 7073 ) | 2.626 ( 2.223 to 3.096 ) | 2.752 ( 2.338 to 3.21 ) | 0.22 (-0.01 to 0.44) |
| Brunei Darussalam | 12 ( 8 to 17 ) | 4.934 ( 3.52 to 7.08 ) | 4.674 ( 3.135 to 6.638 ) |  | 21 ( 16 to 28 ) | 5.384 ( 4.28 to 6.951 ) | 4.655 ( 3.65 to 6.198 ) | 0.49 (0.41 to 0.56) |
| Bulgaria | 478 ( 349 to 636 ) | 4.478 ( 3.244 to 6.027 ) | 5.502 ( 4.016 to 7.331 ) |  | 469 ( 339 to 664 ) | 4.084 ( 2.951 to 5.863 ) | 6.91 ( 4.996 to 9.782 ) | -0.27 (-0.56 to 0.01) |
| Burkina Faso | 284 ( 145 to 572 ) | 2.653 ( 1.451 to 4.437 ) | 2.98 ( 1.524 to 6.004 ) |  | 432 ( 225 to 828 ) | 1.845 ( 1.14 to 2.986 ) | 1.899 ( 0.987 to 3.638 ) | -1.09 (-1.4 to -0.78) |
| Burundi | 686 ( 436 to 1012 ) | 16.934 ( 10.479 to 27.812 ) | 12.345 ( 7.844 to 18.218 ) |  | 1382 ( 857 to 2283 ) | 16.798 ( 10.03 to 29.023 ) | 10.453 ( 6.481 to 17.272 ) | 0.07 (-0.01 to 0.16) |
| Cabo Verde | 6 ( 3 to 12 ) | 1.751 ( 0.843 to 2.834 ) | 1.8 ( 0.92 to 3.411 ) |  | 13 ( 7 to 23 ) | 3.082 ( 1.527 to 5.198 ) | 2.381 ( 1.237 to 4.068 ) | 2.25 (1.93 to 2.57) |
| Cambodia | 73 ( 33 to 121 ) | 0.829 ( 0.411 to 1.319 ) | 0.707 ( 0.32 to 1.18 ) |  | 179 ( 90 to 284 ) | 1.166 ( 0.606 to 1.822 ) | 1.048 ( 0.531 to 1.667 ) | 1.24 (1.15 to 1.33) |
| Cameroon | 256 ( 129 to 503 ) | 2.414 ( 1.313 to 3.892 ) | 2.457 ( 1.235 to 4.82 ) |  | 518 ( 254 to 1014 ) | 1.846 ( 1.08 to 2.956 ) | 1.631 ( 0.801 to 3.192 ) | -0.82 (-1.07 to -0.58) |
| Canada | 2083 ( 1811 to 2380 ) | 7.315 ( 6.327 to 8.418 ) | 7.644 ( 6.647 to 8.732 ) |  | 3098 ( 2571 to 3767 ) | 5.734 ( 4.741 to 7.056 ) | 8.267 ( 6.862 to 10.054 ) | -0.54 (-0.66 to -0.42) |
| Central African Republic | 66 ( 40 to 110 ) | 2.915 ( 1.769 to 5.403 ) | 2.403 ( 1.449 to 4.013 ) |  | 122 ( 70 to 206 ) | 2.863 ( 1.592 to 5.414 ) | 2.233 ( 1.277 to 3.752 ) | -0.02 (-0.16 to 0.12) |
| Chad | 155 ( 81 to 304 ) | 2.173 ( 1.302 to 3.669 ) | 2.579 ( 1.346 to 5.037 ) |  | 352 ( 172 to 680 ) | 1.763 ( 1.04 to 2.842 ) | 1.985 ( 0.971 to 3.83 ) | -0.7 (-0.82 to -0.57) |
| Chile | 364 ( 279 to 456 ) | 3.069 ( 2.376 to 3.852 ) | 2.738 ( 2.101 to 3.433 ) |  | 549 ( 443 to 674 ) | 2.745 ( 2.166 to 3.549 ) | 2.921 ( 2.356 to 3.585 ) | -0.14 (-0.56 to 0.29) |
| China | 12584 ( 7845 to 17173 ) | 1.239 ( 0.771 to 1.667 ) | 1.07 ( 0.667 to 1.46 ) |  | 30538 ( 17060 to 39562 ) | 2.378 ( 1.239 to 3.297 ) | 2.146 ( 1.199 to 2.781 ) | 3.44 (3.01 to 3.87) |
| Colombia | 849 ( 652 to 1096 ) | 3.091 ( 2.413 to 4.052 ) | 2.612 ( 2.008 to 3.372 ) |  | 1509 ( 1176 to 1951 ) | 3.178 ( 2.448 to 4.129 ) | 3.077 ( 2.397 to 3.977 ) | 1.26 (0.9 to 1.62) |
| Comoros | 59 ( 37 to 91 ) | 18.042 ( 10.937 to 29.028 ) | 12.814 ( 8.05 to 19.694 ) |  | 126 ( 77 to 223 ) | 20.385 ( 12.721 to 36.791 ) | 16.867 ( 10.41 to 29.946 ) | 0.17 (0.02 to 0.33) |
| Congo | 54 ( 35 to 89 ) | 2.952 ( 1.78 to 5.646 ) | 2.236 ( 1.453 to 3.725 ) |  | 127 ( 78 to 223 ) | 3.17 ( 1.921 to 5.865 ) | 2.354 ( 1.448 to 4.137 ) | 0.26 (0.09 to 0.43) |
| Cook Islands | 0 ( 0 to 0 ) | 0.74 ( 0.419 to 1.334 ) | 0.666 ( 0.359 to 1.326 ) |  | 0 ( 0 to 1 ) | 3.206 ( 1.104 to 9.204 ) | 2.347 ( 0.959 to 6.194 ) | 2.22 (1.29 to 3.17) |
| Costa Rica | 119 ( 94 to 152 ) | 4.694 ( 3.806 to 5.797 ) | 3.907 ( 3.098 to 5.005 ) |  | 135 ( 107 to 167 ) | 2.859 ( 2.214 to 3.667 ) | 2.838 ( 2.263 to 3.52 ) | -1.06 (-1.55 to -0.56) |
| Coted'Ivoire | 139 ( 78 to 231 ) | 1.649 ( 1.004 to 2.527 ) | 1.138 ( 0.636 to 1.897 ) |  | 374 ( 188 to 616 ) | 1.942 ( 1.119 to 2.95 ) | 1.341 ( 0.676 to 2.21 ) | 0.75 (0.58 to 0.93) |
| Croatia | 365 ( 290 to 446 ) | 6.299 ( 5.003 to 7.743 ) | 7.509 ( 5.974 to 9.175 ) |  | 398 ( 295 to 532 ) | 5.058 ( 3.769 to 6.68 ) | 9.446 ( 7.014 to 12.646 ) | -0.41 (-0.64 to -0.19) |
| Cuba | 519 ( 419 to 636 ) | 5.053 ( 4.05 to 6.162 ) | 4.788 ( 3.859 to 5.867 ) |  | 760 ( 601 to 934 ) | 4.693 ( 3.669 to 5.845 ) | 6.745 ( 5.329 to 8.288 ) | 0.03 (-0.23 to 0.29) |
| Cyprus | 30 ( 23 to 47 ) | 3.694 ( 2.835 to 5.719 ) | 3.916 ( 2.992 to 6.007 ) |  | 85 ( 55 to 118 ) | 4.394 ( 2.857 to 6.031 ) | 6.273 ( 4.031 to 8.724 ) | 1.16 (0.94 to 1.39) |
| Czechia | 727 ( 549 to 942 ) | 5.728 ( 4.387 to 7.37 ) | 7.066 ( 5.331 to 9.147 ) |  | 906 ( 626 to 1276 ) | 5.068 ( 3.653 to 6.893 ) | 8.517 ( 5.889 to 12 ) | -0.34 (-0.43 to -0.25) |
| Democratic People's Republic of Korea | 210 ( 134 to 315 ) | 1.072 ( 0.691 to 1.589 ) | 1.019 ( 0.652 to 1.528 ) |  | 458 ( 268 to 672 ) | 1.788 ( 1.041 to 2.809 ) | 1.736 ( 1.016 to 2.547 ) | 1.95 (1.78 to 2.12) |
| Democratic Republic of the Congo | 792 ( 494 to 1215 ) | 2.681 ( 1.618 to 4.698 ) | 2.076 ( 1.294 to 3.186 ) |  | 1832 ( 1085 to 3262 ) | 3.107 ( 1.743 to 6.065 ) | 2.036 ( 1.206 to 3.624 ) | 0.59 (0.41 to 0.77) |
| Denmark | 498 ( 411 to 593 ) | 8.254 ( 6.88 to 9.865 ) | 9.675 ( 7.986 to 11.529 ) |  | 780 ( 611 to 984 ) | 9.226 ( 7.392 to 11.5 ) | 13.334 ( 10.448 to 16.819 ) | 0.48 (0.31 to 0.65) |
| Djibouti | 46 ( 28 to 72 ) | 17.172 ( 10.344 to 28.719 ) | 11.172 ( 6.717 to 17.487 ) |  | 183 ( 101 to 340 ) | 19.506 ( 11.248 to 36.556 ) | 14.578 ( 8.034 to 27.034 ) | 0.39 (0.31 to 0.47) |
| Dominica | 2 ( 1 to 4 ) | 3.379 ( 1.75 to 6.293 ) | 3.052 ( 1.565 to 5.811 ) |  | 3 ( 2 to 5 ) | 4.313 ( 2.137 to 7.225 ) | 4.913 ( 2.469 to 8.153 ) | 0.94 (0.85 to 1.02) |
| Dominican Republic | 79 ( 42 to 124 ) | 1.211 ( 0.691 to 1.779 ) | 1.104 ( 0.587 to 1.739 ) |  | 131 ( 77 to 192 ) | 1.243 ( 0.729 to 1.816 ) | 1.192 ( 0.697 to 1.742 ) | 0.65 (0.43 to 0.87) |
| Ecuador | 243 ( 175 to 335 ) | 2.951 ( 2.192 to 4.048 ) | 2.431 ( 1.752 to 3.36 ) |  | 710 ( 513 to 965 ) | 4.203 ( 3.043 to 5.694 ) | 3.93 ( 2.84 to 5.34 ) | 1.45 (1.17 to 1.73) |
| Egypt | 253 ( 147 to 492 ) | 0.657 ( 0.395 to 1.329 ) | 0.458 ( 0.266 to 0.889 ) |  | 697 ( 423 to 1307 ) | 0.867 ( 0.535 to 1.634 ) | 0.66 ( 0.401 to 1.237 ) | 1.01 (0.68 to 1.34) |
| El Salvador | 89 ( 54 to 131 ) | 1.973 ( 1.217 to 2.868 ) | 1.668 ( 1.011 to 2.464 ) |  | 174 ( 112 to 248 ) | 2.733 ( 1.755 to 3.907 ) | 2.694 ( 1.733 to 3.842 ) | 1.12 (0.99 to 1.25) |
| Equatorial Guinea | 10 ( 6 to 17 ) | 2.793 ( 1.647 to 5.036 ) | 2.334 ( 1.391 to 3.967 ) |  | 30 ( 17 to 55 ) | 3.122 ( 1.762 to 5.942 ) | 1.973 ( 1.093 to 3.635 ) | 0.33 (0.1 to 0.56) |
| Eritrea | 413 ( 251 to 671 ) | 17.57 ( 10.95 to 28.927 ) | 12.127 ( 7.381 to 19.709 ) |  | 941 ( 538 to 1682 ) | 20.433 ( 11.905 to 36.932 ) | 14.264 ( 8.156 to 25.487 ) | 0.37 (0.31 to 0.43) |
| Estonia | 101 ( 74 to 138 ) | 5.331 ( 3.877 to 7.31 ) | 6.451 ( 4.732 to 8.782 ) |  | 175 ( 126 to 236 ) | 7.973 ( 5.813 to 10.941 ) | 13.33 ( 9.582 to 18.04 ) | 0.4 (0.06 to 0.74) |
| Eswatini | 25 ( 13 to 51 ) | 4.688 ( 2.528 to 10.417 ) | 3.119 ( 1.666 to 6.371 ) |  | 47 ( 23 to 105 ) | 5.418 ( 2.587 to 12.323 ) | 4.107 ( 1.983 to 9.069 ) | 0.59 (0.5 to 0.67) |
| Ethiopia | 3118 ( 1966 to 4723 ) | 8.557 ( 5.055 to 13.452 ) | 6.166 ( 3.888 to 9.341 ) |  | 6224 ( 4078 to 8920 ) | 9.198 ( 5.648 to 13.894 ) | 5.713 ( 3.744 to 8.188 ) | 0.05 (-0.08 to 0.19) |
| Fiji | 5 ( 2 to 13 ) | 0.91 ( 0.391 to 2.322 ) | 0.68 ( 0.277 to 1.685 ) |  | 9 ( 3 to 21 ) | 0.973 ( 0.383 to 2.42 ) | 0.92 ( 0.353 to 2.293 ) | 0.21 (-0.01 to 0.43) |
| Finland | 369 ( 310 to 426 ) | 6.274 ( 5.289 to 7.271 ) | 7.369 ( 6.194 to 8.501 ) |  | 1229 ( 992 to 1540 ) | 14.834 ( 12.193 to 18.414 ) | 22.199 ( 17.923 to 27.813 ) | 2.82 (2.59 to 3.04) |
| France | 5124 ( 4521 to 5717 ) | 7.829 ( 6.943 to 8.813 ) | 8.87 ( 7.826 to 9.896 ) |  | 8481 ( 6681 to 10689 ) | 9.223 ( 7.414 to 11.388 ) | 12.775 ( 10.064 to 16.1 ) | 1.05 (0.85 to 1.24) |
| Gabon | 22 ( 14 to 36 ) | 2.83 ( 1.713 to 5.286 ) | 2.19 ( 1.373 to 3.708 ) |  | 46 ( 28 to 84 ) | 3.331 ( 2.023 to 6.453 ) | 2.557 ( 1.56 to 4.639 ) | 0.55 (0.43 to 0.66) |
| Gambia | 34 ( 20 to 57 ) | 4.593 ( 2.832 to 7.08 ) | 3.481 ( 2.044 to 5.815 ) |  | 102 ( 60 to 175 ) | 5.902 ( 3.562 to 9.464 ) | 4.279 ( 2.486 to 7.305 ) | 0.66 (0.44 to 0.88) |
| Georgia | 149 ( 116 to 190 ) | 2.545 ( 1.954 to 3.269 ) | 2.704 ( 2.098 to 3.44 ) |  | 310 ( 244 to 393 ) | 6.794 ( 5.221 to 8.984 ) | 8.582 ( 6.764 to 10.892 ) | 3.67 (3.24 to 4.1) |
| Germany | 5410 ( 4473 to 6421 ) | 5.506 ( 4.56 to 6.602 ) | 6.768 ( 5.596 to 8.032 ) |  | 11811 ( 9635 to 14216 ) | 8.25 ( 6.757 to 9.855 ) | 13.835 ( 11.286 to 16.652 ) | 1.55 (1.45 to 1.65) |
| Ghana | 202 ( 82 to 462 ) | 0.891 ( 0.4 to 1.985 ) | 1.349 ( 0.547 to 3.088 ) |  | 370 ( 133 to 900 ) | 0.969 ( 0.35 to 2.166 ) | 1.079 ( 0.388 to 2.629 ) | 0.71 (0.39 to 1.04) |
| Greece | 577 ( 504 to 667 ) | 4.291 ( 3.748 to 4.911 ) | 5.551 ( 4.847 to 6.416 ) |  | 922 ( 785 to 1074 ) | 5.225 ( 4.431 to 6.08 ) | 9.063 ( 7.713 to 10.554 ) | 0.99 (0.83 to 1.16) |
| Greenland | 1 ( 0 to 1 ) | 1.56 ( 0.647 to 2.38 ) | 1.294 ( 0.52 to 2.048 ) |  | 1 ( 0 to 1 ) | 0.906 ( 0.614 to 1.357 ) | 1.055 ( 0.698 to 1.517 ) | -0.95 (-1.38 to -0.52) |
| Grenada | 1 ( 1 to 2 ) | 1.475 ( 1.051 to 2.048 ) | 1.32 ( 0.918 to 1.926 ) |  | 2 ( 1 to 2 ) | 1.639 ( 1.218 to 2.18 ) | 1.759 ( 1.324 to 2.322 ) | 0.71 (0.46 to 0.97) |
| Guam | 1 ( 0 to 1 ) | 0.423 ( 0.212 to 0.871 ) | 0.37 ( 0.172 to 0.862 ) |  | 1 ( 0 to 2 ) | 0.47 ( 0.202 to 1.221 ) | 0.464 ( 0.212 to 1.053 ) | 1.91 (1.33 to 2.49) |
| Guatemala | 189 ( 122 to 272 ) | 2.586 ( 1.762 to 3.586 ) | 2.249 ( 1.451 to 3.24 ) |  | 378 ( 275 to 500 ) | 2.719 ( 2.021 to 3.552 ) | 2.396 ( 1.742 to 3.169 ) | 0.44 (0.28 to 0.59) |
| Guinea | 265 ( 139 to 524 ) | 3.089 ( 1.809 to 5.526 ) | 4.416 ( 2.323 to 8.739 ) |  | 374 ( 175 to 786 ) | 2.564 ( 1.319 to 4.496 ) | 2.784 ( 1.305 to 5.85 ) | -0.38 (-0.51 to -0.24) |
| Guinea-Bissau | 32 ( 16 to 67 ) | 2.83 ( 1.567 to 4.96 ) | 3.183 ( 1.601 to 6.673 ) |  | 34 ( 18 to 64 ) | 1.87 ( 1.112 to 3.045 ) | 1.645 ( 0.891 to 3.083 ) | -1.08 (-1.23 to -0.92) |
| Guyana | 2 ( 1 to 3 ) | 0.334 ( 0.22 to 0.507 ) | 0.286 ( 0.184 to 0.448 ) |  | 17 ( 11 to 25 ) | 2.375 ( 1.554 to 3.428 ) | 2.225 ( 1.446 to 3.246 ) | 4.95 (3.09 to 6.85) |
| Haiti | 51 ( 26 to 97 ) | 0.908 ( 0.499 to 1.637 ) | 0.794 ( 0.409 to 1.52 ) |  | 107 ( 52 to 183 ) | 0.98 ( 0.5 to 1.587 ) | 0.833 ( 0.407 to 1.426 ) | 0.4 (0.27 to 0.53) |
| Honduras | 89 ( 57 to 136 ) | 2.231 ( 1.415 to 3.313 ) | 1.888 ( 1.216 to 2.89 ) |  | 206 ( 135 to 310 ) | 2.586 ( 1.699 to 3.813 ) | 2.034 ( 1.338 to 3.064 ) | 0.49 (0.43 to 0.55) |
| Hungary | 877 ( 698 to 1108 ) | 6.697 ( 5.365 to 8.478 ) | 8.437 ( 6.714 to 10.662 ) |  | 929 ( 670 to 1254 ) | 5.841 ( 4.246 to 7.912 ) | 9.682 ( 6.977 to 13.07 ) | -0.29 (-0.41 to -0.17) |
| Iceland | 18 ( 16 to 21 ) | 7.012 ( 6.057 to 8.039 ) | 7.109 ( 6.14 to 8.143 ) |  | 39 ( 31 to 49 ) | 8.954 ( 7.33 to 10.958 ) | 11.109 ( 8.808 to 14.022 ) | 0.96 (0.75 to 1.17) |
| India | 8998 ( 5030 to 12565 ) | 0.998 ( 0.577 to 1.353 ) | 1.055 ( 0.59 to 1.473 ) |  | 15453 ( 9610 to 20953 ) | 1.272 ( 0.792 to 1.737 ) | 1.092 ( 0.679 to 1.481 ) | 0.82 (0.57 to 1.06) |
| Indonesia | 1110 ( 572 to 1622 ) | 0.71 ( 0.39 to 1.016 ) | 0.6 ( 0.309 to 0.877 ) |  | 2539 ( 1359 to 3662 ) | 0.972 ( 0.52 to 1.391 ) | 0.91 ( 0.487 to 1.313 ) | 1.05 (0.97 to 1.13) |
| Iran (Islamic Republic of) | 27 ( 5 to 56 ) | 0.055 ( 0.011 to 0.097 ) | 0.047 ( 0.008 to 0.098 ) |  | 88 ( 22 to 132 ) | 0.116 ( 0.029 to 0.176 ) | 0.103 ( 0.026 to 0.154 ) | 3.52 (3.06 to 3.97) |
| Iraq | 175 ( 118 to 274 ) | 1.146 ( 0.808 to 1.627 ) | 0.952 ( 0.639 to 1.489 ) |  | 748 ( 468 to 1142 ) | 2.069 ( 1.327 to 3.021 ) | 1.815 ( 1.135 to 2.771 ) | 2.09 (1.94 to 2.24) |
| Ireland | 307 ( 266 to 357 ) | 7.951 ( 6.914 to 9.189 ) | 8.523 ( 7.372 to 9.91 ) |  | 450 ( 347 to 572 ) | 6.728 ( 5.217 to 8.567 ) | 9.102 ( 7.018 to 11.581 ) | 0.23 (0.01 to 0.45) |
| Israel | 135 ( 109 to 166 ) | 2.764 ( 2.222 to 3.386 ) | 2.731 ( 2.189 to 3.347 ) |  | 374 ( 306 to 455 ) | 3.485 ( 2.831 to 4.253 ) | 3.901 ( 3.191 to 4.745 ) | 1.2 (1.05 to 1.35) |
| Italy | 4358 ( 3943 to 4849 ) | 6.345 ( 5.722 to 7.072 ) | 7.672 ( 6.942 to 8.537 ) |  | 8054 ( 6719 to 9762 ) | 8.586 ( 7.364 to 10.003 ) | 13.465 ( 11.234 to 16.321 ) | 0.79 (0.54 to 1.04) |
| Jamaica | 44 ( 29 to 68 ) | 1.908 ( 1.314 to 2.78 ) | 1.872 ( 1.243 to 2.884 ) |  | 48 ( 32 to 68 ) | 1.682 ( 1.127 to 2.413 ) | 1.703 ( 1.154 to 2.416 ) | -0.26 (-0.76 to 0.24) |
| Japan | 2365 ( 2157 to 2598 ) | 2.25 ( 1.998 to 2.525 ) | 1.88 ( 1.714 to 2.064 ) |  | 3573 ( 3103 to 4083 ) | 2.414 ( 2.027 to 2.875 ) | 2.798 ( 2.43 to 3.197 ) | 0.76 (0.07 to 1.46) |
| Jordan | 37 ( 25 to 55 ) | 1.363 ( 0.957 to 1.984 ) | 1.001 ( 0.675 to 1.463 ) |  | 177 ( 113 to 254 ) | 1.799 ( 1.151 to 2.525 ) | 1.435 ( 0.92 to 2.061 ) | 1.12 (0.91 to 1.34) |
| Kazakhstan | 540 ( 351 to 811 ) | 3.484 ( 2.297 to 5.128 ) | 3.292 ( 2.141 to 4.947 ) |  | 569 ( 336 to 869 ) | 3.022 ( 1.77 to 4.594 ) | 3.002 ( 1.773 to 4.586 ) | -0.4 (-0.63 to -0.17) |
| Kenya | 4027 ( 2766 to 5432 ) | 20.956 ( 14.223 to 29.043 ) | 17.396 ( 11.948 to 23.465 ) |  | 9449 ( 6544 to 13184 ) | 26.155 ( 16.953 to 37.289 ) | 18.874 ( 13.072 to 26.336 ) | 0.92 (0.78 to 1.06) |
| Kiribati | 1 ( 0 to 1 ) | 0.97 ( 0.476 to 2.1 ) | 0.76 ( 0.362 to 1.557 ) |  | 1 ( 1 to 2 ) | 1.093 ( 0.513 to 2.604 ) | 0.88 ( 0.428 to 1.977 ) | 0.14 (0.01 to 0.28) |
| Kuwait | 26 ( 20 to 36 ) | 1.915 ( 1.527 to 2.379 ) | 1.531 ( 1.153 to 2.074 ) |  | 43 ( 34 to 56 ) | 1.186 ( 0.935 to 1.512 ) | 0.923 ( 0.732 to 1.198 ) | -0.13 (-2.13 to 1.91) |
| Kyrgyzstan | 136 ( 93 to 200 ) | 3.6 ( 2.591 to 5.095 ) | 3.038 ( 2.093 to 4.477 ) |  | 279 ( 192 to 408 ) | 4.397 ( 3.063 to 6.294 ) | 4.066 ( 2.804 to 5.951 ) | 0.56 (-0.71 to 1.86) |
| Lao People's Democratic Republic | 28 ( 12 to 49 ) | 0.741 ( 0.339 to 1.226 ) | 0.673 ( 0.283 to 1.18 ) |  | 56 ( 27 to 90 ) | 0.874 ( 0.431 to 1.361 ) | 0.766 ( 0.366 to 1.217 ) | 0.6 (0.56 to 0.64) |
| Latvia | 130 ( 96 to 173 ) | 4.045 ( 2.982 to 5.266 ) | 4.901 ( 3.629 to 6.503 ) |  | 202 ( 150 to 271 ) | 6.231 ( 4.684 to 8.239 ) | 10.799 ( 8.015 to 14.482 ) | 0.86 (0.57 to 1.15) |
| Lebanon | 52 ( 37 to 86 ) | 2.104 ( 1.484 to 3.527 ) | 1.75 ( 1.23 to 2.873 ) |  | 170 ( 119 to 259 ) | 2.976 ( 2.064 to 4.427 ) | 3.073 ( 2.146 to 4.681 ) | 1.4 (1.29 to 1.52) |
| Lesotho | 48 ( 25 to 103 ) | 4.031 ( 2.039 to 9.111 ) | 3.116 ( 1.629 to 6.707 ) |  | 73 ( 35 to 164 ) | 4.906 ( 2.373 to 11.355 ) | 3.89 ( 1.889 to 8.749 ) | 0.86 (0.72 to 1) |
| Liberia | 55 ( 27 to 116 ) | 2.032 ( 1.018 to 3.598 ) | 2.237 ( 1.104 to 4.696 ) |  | 78 ( 41 to 168 ) | 1.76 ( 1.031 to 3.046 ) | 1.428 ( 0.747 to 3.069 ) | -0.51 (-0.92 to -0.09) |
| Libya | 49 ( 29 to 92 ) | 1.197 ( 0.754 to 1.924 ) | 1.173 ( 0.698 to 2.189 ) |  | 95 ( 57 to 145 ) | 1.74 ( 1.035 to 2.76 ) | 1.376 ( 0.825 to 2.115 ) | 1.2 (0.98 to 1.42) |
| Lithuania | 228 ( 174 to 302 ) | 5.405 ( 4.107 to 7.178 ) | 6.203 ( 4.729 to 8.216 ) |  | 381 ( 290 to 498 ) | 8.098 ( 6.08 to 10.739 ) | 13.973 ( 10.641 to 18.253 ) | 0.8 (0.55 to 1.05) |
| Luxembourg | 25 ( 22 to 29 ) | 5.164 ( 4.543 to 5.827 ) | 6.653 ( 5.863 to 7.488 ) |  | 44 ( 35 to 55 ) | 4.58 ( 3.666 to 5.677 ) | 6.828 ( 5.46 to 8.468 ) | 0.12 (-0.04 to 0.29) |
| Madagascar | 1354 ( 906 to 2065 ) | 15.905 ( 10.587 to 26.154 ) | 11.376 ( 7.616 to 17.351 ) |  | 2987 ( 1879 to 5052 ) | 15.225 ( 9.518 to 26.643 ) | 10.46 ( 6.581 to 17.689 ) | -0.19 (-0.27 to -0.1) |
| Malawi | 2992 ( 1924 to 4563 ) | 43.459 ( 26.966 to 65.182 ) | 30.512 ( 19.616 to 46.535 ) |  | 6894 ( 3788 to 10720 ) | 56.4 ( 31.393 to 87.209 ) | 35.45 ( 19.476 to 55.121 ) | 0.94 (0.9 to 0.98) |
| Malaysia | 164 ( 86 to 281 ) | 1.055 ( 0.57 to 1.664 ) | 0.93 ( 0.485 to 1.591 ) |  | 414 ( 224 to 608 ) | 1.461 ( 0.777 to 2.197 ) | 1.302 ( 0.703 to 1.912 ) | 1.3 (1.12 to 1.48) |
| Maldives | 1 ( 0 to 1 ) | 0.402 ( 0.237 to 0.658 ) | 0.295 ( 0.167 to 0.486 ) |  | 2 ( 1 to 3 ) | 0.48 ( 0.298 to 0.663 ) | 0.399 ( 0.241 to 0.585 ) | 0.73 (0.53 to 0.93) |
| Mali | 774 ( 473 to 1225 ) | 10.779 ( 6.869 to 15.861 ) | 8.933 ( 5.456 to 14.144 ) |  | 1632 ( 876 to 2886 ) | 10.852 ( 6.233 to 18.106 ) | 6.77 ( 3.634 to 11.973 ) | 0.09 (-0.01 to 0.19) |
| Malta | 17 ( 15 to 20 ) | 4.096 ( 3.48 to 4.704 ) | 4.604 ( 3.933 to 5.297 ) |  | 38 ( 29 to 49 ) | 5.015 ( 3.846 to 6.409 ) | 8.612 ( 6.637 to 11.069 ) | 1.24 (1.08 to 1.41) |
| Marshall Islands | 0 ( 0 to 0 ) | 0.502 ( 0.304 to 0.836 ) | 0.362 ( 0.19 to 0.697 ) |  | 0 ( 0 to 1 ) | 0.619 ( 0.336 to 1.035 ) | 0.555 ( 0.288 to 0.964 ) | 0.64 (0.53 to 0.74) |
| Mauritania | 45 ( 24 to 93 ) | 2.146 ( 1.243 to 3.647 ) | 2.207 ( 1.189 to 4.534 ) |  | 89 ( 48 to 163 ) | 2.149 ( 1.381 to 3.428 ) | 2.025 ( 1.093 to 3.717 ) | -0.24 (-0.51 to 0.04) |
| Mauritius | 5 ( 4 to 6 ) | 0.492 ( 0.395 to 0.603 ) | 0.431 ( 0.342 to 0.536 ) |  | 20 ( 17 to 24 ) | 1.368 ( 1.135 to 1.632 ) | 1.601 ( 1.349 to 1.86 ) | 4.27 (3.19 to 5.37) |
| Mexico | 1930 ( 1522 to 2427 ) | 2.505 ( 2.007 to 3.081 ) | 2.26 ( 1.782 to 2.842 ) |  | 3096 ( 2541 to 3746 ) | 2.578 ( 2.108 to 3.125 ) | 2.395 ( 1.966 to 2.898 ) | 0.11 (-0.1 to 0.33) |
| Micronesia (Federated States of) | 0 ( 0 to 1 ) | 0.554 ( 0.329 to 0.985 ) | 0.45 ( 0.24 to 0.91 ) |  | 1 ( 0 to 1 ) | 0.669 ( 0.405 to 1.097 ) | 0.56 ( 0.334 to 0.942 ) | 0.87 (0.77 to 0.98) |
| Monaco | 0 ( 0 to 0 ) | 0.456 ( 0.272 to 0.639 ) | 0.649 ( 0.366 to 0.91 ) |  | 0 ( 0 to 0 ) | 0.777 ( 0.491 to 1.259 ) | 0.893 ( 0.593 to 1.226 ) | 0.84 (0.51 to 1.17) |
| Mongolia | 33 ( 19 to 53 ) | 1.848 ( 1.107 to 2.94 ) | 1.524 ( 0.871 to 2.473 ) |  | 84 ( 50 to 131 ) | 2.682 ( 1.626 to 4.053 ) | 2.52 ( 1.506 to 3.924 ) | 1.51 (1.32 to 1.7) |
| Montenegro | 12 ( 8 to 16 ) | 1.929 ( 1.287 to 2.521 ) | 1.939 ( 1.295 to 2.543 ) |  | 18 ( 12 to 24 ) | 2.096 ( 1.349 to 2.813 ) | 2.917 ( 1.903 to 3.937 ) | 0.4 (0.27 to 0.52) |
| Morocco | 545 ( 321 to 920 ) | 2.079 ( 1.291 to 3.21 ) | 2.15 ( 1.268 to 3.629 ) |  | 1094 ( 708 to 1708 ) | 3.202 ( 2.044 to 5.104 ) | 2.943 ( 1.904 to 4.595 ) | 1.71 (1.59 to 1.82) |
| Mozambique | 1970 ( 1312 to 2926 ) | 18.706 ( 12.224 to 28.304 ) | 14.744 ( 9.823 to 21.905 ) |  | 4194 ( 2664 to 6897 ) | 20.983 ( 12.711 to 36.736 ) | 13.498 ( 8.572 to 22.197 ) | 0.5 (0.44 to 0.56) |
| Myanmar | 311 ( 138 to 548 ) | 0.854 ( 0.409 to 1.467 ) | 0.769 ( 0.341 to 1.354 ) |  | 551 ( 287 to 880 ) | 1.034 ( 0.54 to 1.638 ) | 0.976 ( 0.509 to 1.56 ) | 0.51 (0.43 to 0.6) |
| Namibia | 178 ( 112 to 259 ) | 19.616 ( 12.049 to 29.004 ) | 12.652 ( 7.96 to 18.436 ) |  | 535 ( 300 to 826 ) | 29.046 ( 16.338 to 44.03 ) | 22.018 ( 12.32 to 33.967 ) | 1.3 (1.26 to 1.34) |
| Nauru | 0 ( 0 to 0 ) | 0.6 ( 0.348 to 1.064 ) | 0.533 ( 0.27 to 1.072 ) |  | 0 ( 0 to 0 ) | 0.792 ( 0.44 to 1.391 ) | 0.7 ( 0.356 to 1.369 ) | 0.74 (0.67 to 0.8) |
| Nepal | 263 ( 149 to 506 ) | 1.159 ( 0.68 to 2.022 ) | 1.35 ( 0.764 to 2.601 ) |  | 391 ( 209 to 712 ) | 1.374 ( 0.74 to 2.542 ) | 1.255 ( 0.67 to 2.286 ) | 0.83 (0.6 to 1.06) |
| Netherlands | 695 ( 606 to 798 ) | 4.765 ( 4.089 to 5.532 ) | 4.656 ( 4.064 to 5.348 ) |  | 921 ( 721 to 1151 ) | 4.78 ( 3.635 to 6.007 ) | 5.353 ( 4.191 to 6.689 ) | 0.47 (0.2 to 0.74) |
| New Zealand | 343 ( 286 to 406 ) | 9.07 ( 7.559 to 10.812 ) | 10.038 ( 8.374 to 11.884 ) |  | 711 ( 566 to 889 ) | 9.361 ( 7.478 to 11.728 ) | 13.757 ( 10.941 to 17.189 ) | 0.43 (0.31 to 0.55) |
| Nicaragua | 108 ( 63 to 223 ) | 2.795 ( 1.797 to 4.62 ) | 2.782 ( 1.616 to 5.732 ) |  | 165 ( 119 to 235 ) | 2.807 ( 2.023 to 3.897 ) | 2.475 ( 1.783 to 3.523 ) | 0.53 (0.28 to 0.79) |
| Niger | 260 ( 127 to 592 ) | 2.588 ( 1.45 to 4.828 ) | 3.239 ( 1.584 to 7.372 ) |  | 451 ( 222 to 973 ) | 1.695 ( 0.993 to 2.888 ) | 1.799 ( 0.885 to 3.884 ) | -1.37 (-1.59 to -1.15) |
| Nigeria | 3765 ( 1908 to 5365 ) | 5.507 ( 2.796 to 8.311 ) | 4.181 ( 2.119 to 5.958 ) |  | 9739 ( 4138 to 15419 ) | 6.351 ( 3.148 to 9.555 ) | 4.213 ( 1.79 to 6.67 ) | 0.45 (0.32 to 0.57) |
| Niue | 0 ( 0 to 0 ) | 0.79 ( 0.429 to 1.514 ) | 0.784 ( 0.418 to 1.573 ) |  | 0 ( 0 to 0 ) | 2.581 ( 0.98 to 7.616 ) | 2.088 ( 0.909 to 5.794 ) | 1.8 (1.04 to 2.56) |
| North Macedonia | 73 ( 47 to 102 ) | 3.768 ( 2.405 to 5.254 ) | 3.651 ( 2.35 to 5.129 ) |  | 122 ( 80 to 177 ) | 4.033 ( 2.631 to 5.778 ) | 5.628 ( 3.679 to 8.126 ) | 0.3 (0.09 to 0.52) |
| Northern Mariana Islands | 0 ( 0 to 0 ) | 0.714 ( 0.367 to 1.095 ) | 0.623 ( 0.289 to 1.07 ) |  | 0 ( 0 to 1 ) | 0.882 ( 0.44 to 1.285 ) | 0.914 ( 0.451 to 1.32 ) | 0.67 (0.04 to 1.31) |
| Norway | 431 ( 388 to 477 ) | 7.412 ( 6.719 to 8.155 ) | 10.147 ( 9.136 to 11.232 ) |  | 188 ( 154 to 234 ) | 2.174 ( 1.79 to 2.685 ) | 3.476 ( 2.844 to 4.315 ) | -4.32 (-4.85 to -3.79) |
| Oman | 17 ( 8 to 30 ) | 1.323 ( 0.555 to 2.129 ) | 0.879 ( 0.4 to 1.5 ) |  | 49 ( 20 to 78 ) | 1.516 ( 0.6 to 2.352 ) | 1.036 ( 0.415 to 1.656 ) | 0.78 (0.55 to 1.01) |
| Pakistan | 2330 ( 1505 to 3438 ) | 2.621 ( 1.662 to 4.095 ) | 2.097 ( 1.354 to 3.093 ) |  | 7001 ( 4186 to 10245 ) | 3.617 ( 2.226 to 5.271 ) | 2.972 ( 1.777 to 4.349 ) | 1.05 (1 to 1.09) |
| Palau | 0 ( 0 to 0 ) | 0.381 ( 0.185 to 0.758 ) | 0.346 ( 0.161 to 0.701 ) |  | 0 ( 0 to 0 ) | 0.494 ( 0.217 to 1.217 ) | 0.401 ( 0.188 to 0.805 ) | 0.77 (0.67 to 0.87) |
| Palestine | 10 ( 5 to 15 ) | 0.677 ( 0.372 to 1.002 ) | 0.469 ( 0.248 to 0.718 ) |  | 31 ( 16 to 43 ) | 0.818 ( 0.432 to 1.103 ) | 0.598 ( 0.305 to 0.833 ) | 0.77 (0.52 to 1.02) |
| Panama | 52 ( 37 to 77 ) | 2.343 ( 1.728 to 3.235 ) | 2.174 ( 1.533 to 3.224 ) |  | 116 ( 86 to 158 ) | 2.788 ( 2.027 to 3.868 ) | 2.709 ( 1.993 to 3.688 ) | 0.71 (0.64 to 0.77) |
| Papua New Guinea | 17 ( 8 to 36 ) | 0.481 ( 0.276 to 0.809 ) | 0.406 ( 0.197 to 0.867 ) |  | 56 ( 28 to 113 ) | 0.587 ( 0.321 to 1.057 ) | 0.531 ( 0.268 to 1.077 ) | 0.58 (0.5 to 0.65) |
| Paraguay | 97 ( 58 to 149 ) | 2.677 ( 1.741 to 4.059 ) | 2.406 ( 1.439 to 3.692 ) |  | 241 ( 154 to 379 ) | 3.758 ( 2.412 to 5.878 ) | 3.357 ( 2.155 to 5.289 ) | 1.36 (1.22 to 1.5) |
| Peru | 552 ( 355 to 866 ) | 2.653 ( 1.778 to 3.98 ) | 2.554 ( 1.64 to 4.004 ) |  | 1237 ( 789 to 1890 ) | 3.67 ( 2.325 to 5.619 ) | 3.41 ( 2.174 to 5.21 ) | 1.7 (1.49 to 1.92) |
| Philippines | 988 ( 504 to 1435 ) | 1.752 ( 0.933 to 2.418 ) | 1.569 ( 0.801 to 2.278 ) |  | 2149 ( 1099 to 2904 ) | 2.07 ( 1.097 to 2.798 ) | 1.897 ( 0.97 to 2.564 ) | 0.63 (0.54 to 0.71) |
| Poland | 2992 ( 2487 to 3527 ) | 7.16 ( 5.954 to 8.452 ) | 7.838 ( 6.515 to 9.241 ) |  | 3708 ( 3207 to 4222 ) | 5.976 ( 5.2 to 6.848 ) | 9.698 ( 8.386 to 11.041 ) | -0.75 (-1.02 to -0.49) |
| Portugal | 759 ( 644 to 886 ) | 7.614 ( 6.366 to 9.031 ) | 7.484 ( 6.349 to 8.742 ) |  | 1123 ( 946 to 1360 ) | 8.908 ( 7.325 to 10.469 ) | 10.583 ( 8.918 to 12.818 ) | 0.92 (0.45 to 1.39) |
| Puerto Rico | 49 ( 39 to 63 ) | 1.388 ( 1.092 to 1.753 ) | 1.368 ( 1.075 to 1.73 ) |  | 77 ( 61 to 96 ) | 1.49 ( 1.17 to 1.868 ) | 2.326 ( 1.837 to 2.91 ) | 0.54 (-0.25 to 1.33) |
| Qatar | 3 ( 2 to 4 ) | 1.278 ( 0.788 to 2.123 ) | 0.589 ( 0.358 to 0.981 ) |  | 23 ( 11 to 42 ) | 1.578 ( 0.769 to 2.608 ) | 0.762 ( 0.386 to 1.42 ) | 1.43 (0.87 to 1.99) |
| Republic of Korea | 759 ( 445 to 1081 ) | 2.019 ( 1.188 to 2.877 ) | 1.715 ( 1.007 to 2.444 ) |  | 1280 ( 901 to 1661 ) | 3.694 ( 2.641 to 5.051 ) | 2.481 ( 1.747 to 3.222 ) | 2.35 (2.08 to 2.62) |
| Republic of Moldova | 185 ( 136 to 266 ) | 4.131 ( 3.029 to 5.972 ) | 4.165 ( 3.065 to 5.977 ) |  | 187 ( 153 to 228 ) | 3.818 ( 3.012 to 4.715 ) | 5.21 ( 4.245 to 6.343 ) | -0.29 (-0.52 to -0.06) |
| Romania | 1239 ( 854 to 1704 ) | 4.988 ( 3.383 to 6.896 ) | 5.3 ( 3.652 to 7.286 ) |  | 1629 ( 1264 to 2085 ) | 5.764 ( 4.454 to 7.39 ) | 8.6 ( 6.672 to 11.011 ) | 0.24 (0.05 to 0.43) |
| Russian Federation | 7078 ( 5195 to 9218 ) | 4.305 ( 3.174 to 5.567 ) | 4.688 ( 3.441 to 6.105 ) |  | 9975 ( 8595 to 11314 ) | 4.964 ( 4.279 to 5.619 ) | 6.886 ( 5.933 to 7.811 ) | 0.26 (0.07 to 0.45) |
| Rwanda | 999 ( 649 to 1530 ) | 18.88 ( 12.152 to 30.369 ) | 13.898 ( 9.028 to 21.279 ) |  | 1802 ( 1100 to 3198 ) | 19.29 ( 11.635 to 35.197 ) | 13.581 ( 8.291 to 24.097 ) | 0.04 (-0.22 to 0.3) |
| Saint Kitts and Nevis | 0 ( 0 to 0 ) | 0.315 ( 0.208 to 0.446 ) | 0.307 ( 0.199 to 0.452 ) |  | 0 ( 0 to 0 ) | 0.244 ( 0.176 to 0.324 ) | 0.243 ( 0.18 to 0.309 ) | -0.79 (-0.98 to -0.6) |
| Saint Lucia | 0 ( 0 to 1 ) | 0.343 ( 0.259 to 0.447 ) | 0.293 ( 0.215 to 0.39 ) |  | 1 ( 1 to 1 ) | 0.388 ( 0.292 to 0.495 ) | 0.44 ( 0.332 to 0.555 ) | 0.43 (0.08 to 0.77) |
| Saint Vincent and the Grenadines | 8 ( 6 to 11 ) | 7.161 ( 5.27 to 10.163 ) | 7.062 ( 5.03 to 10.326 ) |  | 4 ( 3 to 5 ) | 3.108 ( 2.33 to 3.946 ) | 3.297 ( 2.507 to 4.133 ) | -2.27 (-2.74 to -1.8) |
| Samoa | 5 ( 2 to 11 ) | 4.259 ( 2.154 to 11.694 ) | 2.706 ( 1.431 to 6.654 ) |  | 9 ( 5 to 20 ) | 5.256 ( 2.718 to 12.329 ) | 4.141 ( 2.182 to 9.225 ) | 0.69 (0.65 to 0.73) |
| San Marino | 1 ( 1 to 2 ) | 4.514 ( 3.041 to 6.212 ) | 5.747 ( 3.943 to 7.994 ) |  | 2 ( 1 to 3 ) | 3.173 ( 1.832 to 4.876 ) | 5.23 ( 3.017 to 8.277 ) | -0.3 (-0.61 to 0.02) |
| Sao Tome and Principe | 3 ( 2 to 6 ) | 2.326 ( 1.271 to 4.023 ) | 2.436 ( 1.253 to 4.942 ) |  | 4 ( 2 to 9 ) | 2.402 ( 1.419 to 4.07 ) | 2.057 ( 1.033 to 3.973 ) | 0.19 (-0.33 to 0.72) |
| Saudi Arabia | 122 ( 39 to 213 ) | 1.017 ( 0.382 to 1.633 ) | 0.772 ( 0.247 to 1.341 ) |  | 467 ( 174 to 737 ) | 1.578 ( 0.589 to 2.432 ) | 1.239 ( 0.46 to 1.956 ) | 1.9 (1.39 to 2.42) |
| Senegal | 221 ( 110 to 462 ) | 2.582 ( 1.4 to 4.517 ) | 2.897 ( 1.442 to 6.053 ) |  | 319 ( 169 to 610 ) | 2.163 ( 1.301 to 3.501 ) | 2.013 ( 1.066 to 3.847 ) | -0.41 (-0.71 to -0.12) |
| Serbia | 474 ( 302 to 659 ) | 4.565 ( 2.928 to 6.317 ) | 4.919 ( 3.133 to 6.84 ) |  | 707 ( 428 to 941 ) | 4.864 ( 3.026 to 6.545 ) | 7.931 ( 4.794 to 10.545 ) | 0.24 (0.13 to 0.35) |
| Seychelles | 1 ( 1 to 1 ) | 1.468 ( 0.877 to 2.065 ) | 1.219 ( 0.767 to 1.714 ) |  | 2 ( 1 to 2 ) | 1.34 ( 0.773 to 1.831 ) | 1.5 ( 0.834 to 2.032 ) | 0.02 (-0.09 to 0.13) |
| Sierra Leone | 99 ( 49 to 186 ) | 2.144 ( 1.121 to 3.642 ) | 2.396 ( 1.19 to 4.476 ) |  | 138 ( 71 to 286 ) | 1.699 ( 0.974 to 2.85 ) | 1.56 ( 0.801 to 3.226 ) | -0.72 (-0.95 to -0.48) |
| Singapore | 94 ( 74 to 115 ) | 4.315 ( 3.324 to 5.269 ) | 3.094 ( 2.439 to 3.763 ) |  | 171 ( 131 to 215 ) | 4.351 ( 3.069 to 5.654 ) | 2.983 ( 2.295 to 3.754 ) | 0.28 (-0.14 to 0.71) |
| Slovakia | 322 ( 215 to 440 ) | 5.554 ( 3.782 to 7.611 ) | 6.095 ( 4.075 to 8.325 ) |  | 467 ( 307 to 642 ) | 5.773 ( 3.762 to 7.784 ) | 8.606 ( 5.652 to 11.822 ) | 0.23 (0.16 to 0.31) |
| Slovenia | 100 ( 78 to 126 ) | 4.326 ( 3.391 to 5.414 ) | 5.071 ( 3.948 to 6.397 ) |  | 124 ( 92 to 168 ) | 3.674 ( 2.693 to 4.903 ) | 5.996 ( 4.423 to 8.098 ) | -0.17 (-0.38 to 0.04) |
| Solomon Islands | 1 ( 1 to 3 ) | 0.437 ( 0.217 to 0.786 ) | 0.364 ( 0.158 to 0.851 ) |  | 4 ( 2 to 8 ) | 0.639 ( 0.376 to 1.15 ) | 0.56 ( 0.295 to 1.121 ) | 1.24 (1.03 to 1.46) |
| Somalia | 937 ( 569 to 1521 ) | 18.275 ( 10.855 to 31.786 ) | 11.808 ( 7.172 to 19.164 ) |  | 2236 ( 1195 to 3904 ) | 18.434 ( 10.073 to 32.962 ) | 10.348 ( 5.53 to 18.068 ) | 0.03 (-0.04 to 0.1) |
| South Africa | 625 ( 410 to 868 ) | 2.015 ( 1.317 to 2.924 ) | 1.688 ( 1.108 to 2.344 ) |  | 1475 ( 847 to 2096 ) | 2.805 ( 1.599 to 3.982 ) | 2.594 ( 1.49 to 3.687 ) | 1.29 (1.04 to 1.54) |
| South Sudan | 634 ( 400 to 1048 ) | 15.705 ( 9.818 to 27.093 ) | 10.793 ( 6.809 to 17.824 ) |  | 1137 ( 680 to 1990 ) | 18.317 ( 10.63 to 32.895 ) | 11.752 ( 7.028 to 20.569 ) | 0.39 (0.2 to 0.58) |
| Spain | 2426 ( 2160 to 2737 ) | 5.837 ( 5.135 to 6.713 ) | 6.254 ( 5.57 to 7.058 ) |  | 4197 ( 3388 to 5188 ) | 6.988 ( 5.458 to 8.561 ) | 9.215 ( 7.437 to 11.39 ) | 0.93 (0.67 to 1.2) |
| Sri Lanka | 212 ( 152 to 300 ) | 1.456 ( 1.029 to 2.028 ) | 1.238 ( 0.889 to 1.749 ) |  | 411 ( 240 to 603 ) | 1.739 ( 1.031 to 2.599 ) | 1.844 ( 1.08 to 2.71 ) | 0.98 (0.77 to 1.2) |
| Sudan | 15 ( 3 to 53 ) | 0.102 ( 0.015 to 0.369 ) | 0.075 ( 0.013 to 0.263 ) |  | 49 ( 9 to 161 ) | 0.16 ( 0.031 to 0.546 ) | 0.112 ( 0.022 to 0.37 ) | 1.76 (1.57 to 1.95) |
| Suriname | 5 ( 3 to 9 ) | 1.612 ( 0.967 to 2.478 ) | 1.417 ( 0.779 to 2.28 ) |  | 12 ( 7 to 19 ) | 2.001 ( 1.146 to 3.169 ) | 2.064 ( 1.193 to 3.24 ) | 1.1 (0.87 to 1.33) |
| Sweden | 392 ( 333 to 464 ) | 3.081 ( 2.606 to 3.642 ) | 4.566 ( 3.876 to 5.398 ) |  | 976 ( 753 to 1237 ) | 5.31 ( 4.109 to 6.781 ) | 9.41 ( 7.259 to 11.928 ) | 2.57 (2.06 to 3.08) |
| Switzerland | 479 ( 404 to 563 ) | 6.329 ( 5.321 to 7.426 ) | 6.979 ( 5.885 to 8.203 ) |  | 1223 ( 931 to 1551 ) | 10.213 ( 8.144 to 12.662 ) | 13.711 ( 10.431 to 17.383 ) | 1.42 (1.17 to 1.67) |
| Syrian Arab Republic | 20 ( 12 to 31 ) | 0.241 ( 0.153 to 0.384 ) | 0.157 ( 0.097 to 0.245 ) |  | 47 ( 29 to 73 ) | 0.341 ( 0.21 to 0.511 ) | 0.338 ( 0.205 to 0.521 ) | 1.02 (0.93 to 1.12) |
| Taiwan (Province of China) | 317 ( 273 to 368 ) | 1.788 ( 1.55 to 2.064 ) | 1.555 ( 1.341 to 1.804 ) |  | 471 ( 380 to 583 ) | 2.186 ( 1.613 to 2.984 ) | 1.994 ( 1.607 to 2.466 ) | 1.9 (1.36 to 2.44) |
| Tajikistan | 10 ( 3 to 25 ) | 0.126 ( 0.041 to 0.289 ) | 0.187 ( 0.049 to 0.458 ) |  | 30 ( 7 to 81 ) | 0.236 ( 0.06 to 0.627 ) | 0.293 ( 0.066 to 0.801 ) | 2.47 (2.25 to 2.7) |
| Thailand | 836 ( 524 to 1161 ) | 1.715 ( 1.084 to 2.344 ) | 1.472 ( 0.923 to 2.045 ) |  | 1628 ( 1003 to 2270 ) | 2.174 ( 1.49 to 2.959 ) | 2.442 ( 1.504 to 3.404 ) | 0.66 (0.43 to 0.9) |
| Timor-Leste | 4 ( 2 to 7 ) | 0.625 ( 0.313 to 0.975 ) | 0.524 ( 0.238 to 0.876 ) |  | 11 ( 5 to 18 ) | 0.89 ( 0.453 to 1.396 ) | 0.78 ( 0.374 to 1.285 ) | 1.43 (1.18 to 1.68) |
| Togo | 82 ( 44 to 162 ) | 2.316 ( 1.308 to 3.9 ) | 2.252 ( 1.204 to 4.436 ) |  | 137 ( 74 to 278 ) | 1.947 ( 1.178 to 3.262 ) | 1.639 ( 0.886 to 3.319 ) | -0.65 (-0.91 to -0.4) |
| Tokelau | 0 ( 0 to 0 ) | 0.618 ( 0.38 to 1.044 ) | 0.548 ( 0.331 to 0.924 ) |  | 0 ( 0 to 0 ) | 11.529 ( 2.95 to 35.867 ) | 8.991 ( 2.38 to 27.656 ) | 3.62 (1.28 to 6.01) |
| Tonga | 0 ( 0 to 1 ) | 0.59 ( 0.343 to 1.032 ) | 0.487 ( 0.259 to 1.043 ) |  | 1 ( 0 to 2 ) | 0.784 ( 0.415 to 1.458 ) | 0.776 ( 0.39 to 1.694 ) | 0.92 (0.82 to 1.03) |
| Trinidad and Tobago | 18 ( 13 to 27 ) | 1.587 ( 1.131 to 2.204 ) | 1.511 ( 1.05 to 2.225 ) |  | 26 ( 18 to 38 ) | 2.053 ( 1.292 to 3.157 ) | 1.901 ( 1.289 to 2.711 ) | 1.62 (1.23 to 2.01) |
| Tunisia | 148 ( 98 to 239 ) | 1.903 ( 1.313 to 2.932 ) | 1.771 ( 1.177 to 2.861 ) |  | 273 ( 176 to 418 ) | 2.379 ( 1.534 to 3.709 ) | 2.309 ( 1.49 to 3.527 ) | 0.67 (0.54 to 0.81) |
| Turkey | 659 ( 459 to 1004 ) | 1.364 ( 0.955 to 2.16 ) | 1.146 ( 0.799 to 1.747 ) |  | 1745 ( 1213 to 2378 ) | 2.201 ( 1.512 to 2.976 ) | 2.087 ( 1.45 to 2.844 ) | 1.83 (1.69 to 1.97) |
| Turkmenistan | 81 ( 52 to 122 ) | 2.705 ( 1.764 to 4.022 ) | 2.194 ( 1.411 to 3.292 ) |  | 138 ( 79 to 222 ) | 2.892 ( 1.7 to 4.646 ) | 2.673 ( 1.534 to 4.312 ) | -0.01 (-0.11 to 0.08) |
| Tuvalu | 0 ( 0 to 0 ) | 0.634 ( 0.362 to 1.09 ) | 0.613 ( 0.33 to 1.174 ) |  | 0 ( 0 to 0 ) | 0.71 ( 0.435 to 1.259 ) | 0.653 ( 0.392 to 1.184 ) | 0.44 (0.31 to 0.56) |
| Uganda | 2426 ( 1448 to 3550 ) | 22.949 ( 13.063 to 35.857 ) | 14.033 ( 8.376 to 20.531 ) |  | 7473 ( 4331 to 11346 ) | 30.113 ( 17.856 to 45.723 ) | 17.253 ( 9.999 to 26.193 ) | 0.66 (0.57 to 0.74) |
| Ukraine | 2598 ( 2090 to 3288 ) | 3.933 ( 3.159 to 5.02 ) | 4.929 ( 3.965 to 6.238 ) |  | 2761 ( 1921 to 3825 ) | 4.443 ( 3.143 to 6.033 ) | 6.409 ( 4.458 to 8.879 ) | 0.49 (0.35 to 0.64) |
| United Arab Emirates | 20 ( 12 to 35 ) | 2.312 ( 1.388 to 4.166 ) | 1.079 ( 0.638 to 1.852 ) |  | 112 ( 77 to 214 ) | 3.076 ( 2.155 to 5.153 ) | 1.165 ( 0.798 to 2.218 ) | 2.63 (2.03 to 3.23) |
| United Kingdom | 5732 ( 5279 to 6200 ) | 7.433 ( 6.881 to 8.034 ) | 10.004 ( 9.213 to 10.82 ) |  | 5964 ( 5304 to 6749 ) | 5.865 ( 5.248 to 6.57 ) | 8.791 ( 7.818 to 9.947 ) | -0.25 (-0.63 to 0.13) |
| United Republic of Tanzania | 3522 ( 2355 to 5332 ) | 19.254 ( 12.544 to 29.891 ) | 13.632 ( 9.114 to 20.637 ) |  | 7617 ( 4619 to 12980 ) | 18.528 ( 11.337 to 32.2 ) | 13.032 ( 7.904 to 22.208 ) | -0.18 (-0.22 to -0.13) |
| United States of America | 13943 ( 12806 to 15163 ) | 5.349 ( 4.892 to 5.855 ) | 5.488 ( 5.04 to 5.968 ) |  | 19307 ( 17217 to 21425 ) | 4.486 ( 4.012 to 5.058 ) | 5.804 ( 5.176 to 6.441 ) | -0.36 (-0.58 to -0.14) |
| United States Virgin Islands | 1 ( 1 to 2 ) | 1.1 ( 0.589 to 2.07 ) | 1.094 ( 0.573 to 2.091 ) |  | 1 ( 0 to 1 ) | 0.832 ( 0.477 to 1.507 ) | 0.661 ( 0.45 to 1.017 ) | -0.97 (-1.36 to -0.59) |
| Uruguay | 125 ( 94 to 162 ) | 3.813 ( 2.821 to 5.081 ) | 3.973 ( 2.985 to 5.158 ) |  | 154 ( 125 to 190 ) | 4.046 ( 3.201 to 5.184 ) | 4.53 ( 3.664 to 5.581 ) | 0 (-0.19 to 0.19) |
| Uzbekistan | 262 ( 153 to 426 ) | 1.425 ( 0.882 to 2.183 ) | 1.248 ( 0.731 to 2.031 ) |  | 650 ( 432 to 944 ) | 1.987 ( 1.319 to 2.877 ) | 1.898 ( 1.261 to 2.756 ) | 1.24 (0.86 to 1.61) |
| Vanuatu | 1 ( 0 to 1 ) | 0.439 ( 0.259 to 0.757 ) | 0.348 ( 0.179 to 0.693 ) |  | 1 ( 1 to 2 ) | 0.555 ( 0.33 to 0.952 ) | 0.462 ( 0.257 to 0.782 ) | 0.63 (0.45 to 0.82) |
| Venezuela (Bolivarian Republic of) | 463 ( 337 to 625 ) | 2.994 ( 2.278 to 3.875 ) | 2.462 ( 1.794 to 3.323 ) |  | 955 ( 674 to 1291 ) | 3.543 ( 2.501 to 4.801 ) | 3.587 ( 2.532 to 4.849 ) | 0.61 (0.45 to 0.77) |
| Viet Nam | 1302 ( 750 to 2270 ) | 1.785 ( 1.09 to 2.86 ) | 1.908 ( 1.1 to 3.327 ) |  | 2606 ( 1674 to 4031 ) | 2.839 ( 1.85 to 4.495 ) | 2.599 ( 1.669 to 4.02 ) | 1.54 (1.42 to 1.65) |
| Yemen | 8 ( 1 to 27 ) | 0.095 ( 0.014 to 0.344 ) | 0.057 ( 0.01 to 0.196 ) |  | 31 ( 6 to 97 ) | 0.147 ( 0.03 to 0.476 ) | 0.091 ( 0.019 to 0.288 ) | 1.71 (1.52 to 1.9) |
| Zambia | 964 ( 625 to 1496 ) | 17.132 ( 11.14 to 27.861 ) | 12.149 ( 7.88 to 18.853 ) |  | 3512 ( 1595 to 6427 ) | 28.828 ( 13.433 to 51.938 ) | 17.995 ( 8.171 to 32.931 ) | 2.09 (1.72 to 2.46) |
| Zimbabwe | 603 ( 360 to 888 ) | 10.197 ( 6.061 to 15.025 ) | 5.832 ( 3.484 to 8.581 ) |  | 1918 ( 815 to 3208 ) | 18.186 ( 7.417 to 30.297 ) | 12.301 ( 5.224 to 20.571 ) | 2.79 (2.01 to 3.57) |

**Supplementary Table S2: Incidence of Eye Cancer in 204 Countries and Territories in 1990 and 2021, with EAPC**

| **Location** | **1990** | | |  | **2021** | | | **EAPC(95%CI)** |
| --- | --- | --- | --- | --- | --- | --- | --- | --- |
|  | **Number(95%UI)** | **ASR(95%UI)** | **Rate(95%UI)** |  | **Number(95%UI)** | **ASR(95%UI)** | **Rate(95%UI)** |  |
| Afghanistan | 1 ( 0 to 5 ) | 0.017 ( 0.002 to 0.071 ) | 0.013 ( 0.002 to 0.055 ) |  | 4 ( 1 to 15 ) | 0.024 ( 0.004 to 0.095 ) | 0.013 ( 0.002 to 0.048 ) | 1.38 (1.19 to 1.57) |
| Albania | 28 ( 18 to 41 ) | 1.194 ( 0.799 to 1.76 ) | 0.843 ( 0.557 to 1.232 ) |  | 46 ( 31 to 72 ) | 1.171 ( 0.792 to 1.838 ) | 1.715 ( 1.156 to 2.709 ) | 0.29 (0.12 to 0.46) |
| Algeria | 28 ( 18 to 43 ) | 0.142 ( 0.089 to 0.203 ) | 0.112 ( 0.071 to 0.168 ) |  | 68 ( 45 to 99 ) | 0.171 ( 0.112 to 0.252 ) | 0.154 ( 0.102 to 0.225 ) | 0.63 (0.52 to 0.74) |
| American Samoa | 0 ( 0 to 0 ) | 0.06 ( 0.035 to 0.091 ) | 0.042 ( 0.023 to 0.069 ) |  | 0 ( 0 to 0 ) | 0.073 ( 0.041 to 0.112 ) | 0.068 ( 0.038 to 0.102 ) | 0.92 (0.41 to 1.44) |
| Andorra | 0 ( 0 to 1 ) | 0.591 ( 0.342 to 0.902 ) | 0.615 ( 0.351 to 0.948 ) |  | 1 ( 0 to 1 ) | 0.45 ( 0.256 to 0.672 ) | 0.718 ( 0.392 to 1.102 ) | -0.63 (-0.85 to -0.4) |
| Angola | 26 ( 16 to 44 ) | 0.353 ( 0.211 to 0.642 ) | 0.257 ( 0.155 to 0.432 ) |  | 77 ( 46 to 132 ) | 0.382 ( 0.219 to 0.73 ) | 0.236 ( 0.139 to 0.404 ) | 0.22 (0.07 to 0.38) |
| Antigua and Barbuda | 0 ( 0 to 0 ) | 0.269 ( 0.214 to 0.335 ) | 0.253 ( 0.2 to 0.315 ) |  | 0 ( 0 to 1 ) | 0.476 ( 0.399 to 0.558 ) | 0.522 ( 0.442 to 0.603 ) | 2.42 (1.6 to 3.24) |
| Argentina | 104 ( 78 to 137 ) | 0.32 ( 0.239 to 0.419 ) | 0.316 ( 0.235 to 0.415 ) |  | 120 ( 97 to 149 ) | 0.242 ( 0.196 to 0.304 ) | 0.263 ( 0.214 to 0.327 ) | -0.48 (-0.79 to -0.17) |
| Armenia | 10 ( 6 to 16 ) | 0.36 ( 0.222 to 0.533 ) | 0.306 ( 0.19 to 0.454 ) |  | 17 ( 11 to 26 ) | 0.464 ( 0.286 to 0.725 ) | 0.578 ( 0.368 to 0.869 ) | 1.01 (0.84 to 1.18) |
| Australia | 151 ( 134 to 170 ) | 0.806 ( 0.718 to 0.911 ) | 0.894 ( 0.795 to 1.01 ) |  | 261 ( 203 to 331 ) | 0.663 ( 0.521 to 0.836 ) | 1.014 ( 0.789 to 1.285 ) | -0.63 (-0.8 to -0.46) |
| Austria | 73 ( 64 to 84 ) | 0.778 ( 0.675 to 0.89 ) | 0.946 ( 0.821 to 1.076 ) |  | 119 ( 95 to 147 ) | 0.845 ( 0.682 to 1.048 ) | 1.326 ( 1.06 to 1.64 ) | 0.94 (0.68 to 1.21) |
| Azerbaijan | 12 ( 7 to 22 ) | 0.185 ( 0.106 to 0.326 ) | 0.161 ( 0.089 to 0.302 ) |  | 20 ( 10 to 39 ) | 0.216 ( 0.108 to 0.432 ) | 0.192 ( 0.097 to 0.374 ) | 0.73 (0.59 to 0.87) |
| Bahamas | 1 ( 1 to 2 ) | 0.623 ( 0.478 to 0.813 ) | 0.55 ( 0.412 to 0.748 ) |  | 2 ( 1 to 3 ) | 0.516 ( 0.383 to 0.676 ) | 0.52 ( 0.385 to 0.683 ) | -0.48 (-0.88 to -0.07) |
| Bahrain | 0 ( 0 to 0 ) | 0.046 ( 0.03 to 0.087 ) | 0.024 ( 0.015 to 0.044 ) |  | 1 ( 0 to 1 ) | 0.09 ( 0.035 to 0.146 ) | 0.058 ( 0.022 to 0.097 ) | 3.01 (2.65 to 3.38) |
| Bangladesh | 189 ( 104 to 326 ) | 0.163 ( 0.095 to 0.279 ) | 0.173 ( 0.096 to 0.298 ) |  | 287 ( 168 to 533 ) | 0.195 ( 0.113 to 0.363 ) | 0.174 ( 0.102 to 0.324 ) | 0.69 (0.5 to 0.88) |
| Barbados | 1 ( 0 to 1 ) | 0.208 ( 0.147 to 0.327 ) | 0.201 ( 0.15 to 0.29 ) |  | 1 ( 1 to 1 ) | 0.318 ( 0.178 to 0.545 ) | 0.268 ( 0.185 to 0.39 ) | 2.16 (1.63 to 2.7) |
| Belarus | 67 ( 53 to 85 ) | 0.56 ( 0.451 to 0.707 ) | 0.646 ( 0.511 to 0.815 ) |  | 140 ( 105 to 188 ) | 1.086 ( 0.797 to 1.507 ) | 1.501 ( 1.127 to 2.015 ) | 1.94 (1.7 to 2.18) |
| Belgium | 92 ( 76 to 110 ) | 0.75 ( 0.617 to 0.883 ) | 0.926 ( 0.76 to 1.102 ) |  | 144 ( 114 to 181 ) | 0.834 ( 0.659 to 1.024 ) | 1.258 ( 0.991 to 1.576 ) | 0.63 (0.32 to 0.93) |
| Belize | 0 ( 0 to 0 ) | 0.054 ( 0.033 to 0.096 ) | 0.071 ( 0.039 to 0.138 ) |  | 0 ( 0 to 0 ) | 0.024 ( 0.019 to 0.033 ) | 0.021 ( 0.016 to 0.028 ) | -2.22 (-3.26 to -1.16) |
| Benin | 16 ( 9 to 32 ) | 0.311 ( 0.189 to 0.501 ) | 0.338 ( 0.176 to 0.663 ) |  | 30 ( 15 to 62 ) | 0.24 ( 0.141 to 0.399 ) | 0.225 ( 0.112 to 0.459 ) | -0.94 (-1.15 to -0.74) |
| Bermuda | 0 ( 0 to 0 ) | 0.044 ( 0.035 to 0.057 ) | 0.044 ( 0.035 to 0.055 ) |  | 0 ( 0 to 0 ) | 0.051 ( 0.039 to 0.068 ) | 0.093 ( 0.07 to 0.123 ) | 0.33 (-0.04 to 0.7) |
| Bhutan | 1 ( 0 to 2 ) | 0.136 ( 0.075 to 0.247 ) | 0.129 ( 0.063 to 0.27 ) |  | 1 ( 1 to 2 ) | 0.205 ( 0.105 to 0.394 ) | 0.172 ( 0.089 to 0.329 ) | 1.44 (1.28 to 1.6) |
| Bolivia (Plurinational State of) | 23 ( 14 to 38 ) | 0.394 ( 0.26 to 0.6 ) | 0.356 ( 0.226 to 0.597 ) |  | 44 ( 29 to 69 ) | 0.44 ( 0.287 to 0.677 ) | 0.377 ( 0.243 to 0.588 ) | 0.39 (0.25 to 0.53) |
| Bosnia and Herzegovina | 21 ( 15 to 30 ) | 0.513 ( 0.357 to 0.736 ) | 0.464 ( 0.325 to 0.664 ) |  | 30 ( 20 to 41 ) | 0.537 ( 0.363 to 0.732 ) | 0.913 ( 0.62 to 1.246 ) | 0.4 (0.29 to 0.51) |
| Botswana | 5 ( 3 to 10 ) | 0.581 ( 0.309 to 1.271 ) | 0.376 ( 0.204 to 0.782 ) |  | 11 ( 5 to 22 ) | 0.581 ( 0.304 to 1.183 ) | 0.453 ( 0.229 to 0.919 ) | 0.07 (-0.07 to 0.21) |
| Brazil | 370 ( 293 to 464 ) | 0.317 ( 0.248 to 0.397 ) | 0.249 ( 0.197 to 0.312 ) |  | 751 ( 635 to 881 ) | 0.324 ( 0.274 to 0.381 ) | 0.341 ( 0.288 to 0.4 ) | 0.14 (-0.07 to 0.35) |
| Brunei Darussalam | 1 ( 1 to 2 ) | 0.595 ( 0.433 to 0.854 ) | 0.532 ( 0.359 to 0.756 ) |  | 2 ( 2 to 3 ) | 0.631 ( 0.504 to 0.822 ) | 0.534 ( 0.42 to 0.716 ) | 0.42 (0.34 to 0.5) |
| Bulgaria | 62 ( 46 to 82 ) | 0.607 ( 0.444 to 0.805 ) | 0.716 ( 0.525 to 0.944 ) |  | 64 ( 46 to 89 ) | 0.541 ( 0.39 to 0.768 ) | 0.947 ( 0.679 to 1.317 ) | -0.37 (-0.68 to -0.05) |
| Burkina Faso | 33 ( 17 to 65 ) | 0.338 ( 0.191 to 0.543 ) | 0.346 ( 0.181 to 0.685 ) |  | 50 ( 27 to 93 ) | 0.236 ( 0.148 to 0.368 ) | 0.219 ( 0.117 to 0.41 ) | -1.11 (-1.4 to -0.83) |
| Burundi | 85 ( 54 to 127 ) | 2.279 ( 1.401 to 3.737 ) | 1.528 ( 0.976 to 2.293 ) |  | 165 ( 104 to 278 ) | 2.158 ( 1.306 to 3.803 ) | 1.251 ( 0.783 to 2.104 ) | -0.23 (-0.29 to -0.17) |
| Cabo Verde | 1 ( 0 to 1 ) | 0.227 ( 0.1 to 0.361 ) | 0.221 ( 0.107 to 0.4 ) |  | 2 ( 1 to 3 ) | 0.388 ( 0.176 to 0.639 ) | 0.297 ( 0.143 to 0.49 ) | 2.13 (1.83 to 2.43) |
| Cambodia | 9 ( 4 to 14 ) | 0.106 ( 0.054 to 0.166 ) | 0.083 ( 0.039 to 0.136 ) |  | 21 ( 11 to 34 ) | 0.147 ( 0.077 to 0.226 ) | 0.125 ( 0.064 to 0.197 ) | 1.19 (1.11 to 1.28) |
| Cameroon | 30 ( 15 to 57 ) | 0.307 ( 0.171 to 0.485 ) | 0.285 ( 0.145 to 0.548 ) |  | 60 ( 30 to 115 ) | 0.236 ( 0.139 to 0.375 ) | 0.189 ( 0.094 to 0.361 ) | -0.83 (-1.04 to -0.61) |
| Canada | 255 ( 222 to 291 ) | 0.886 ( 0.769 to 1.021 ) | 0.937 ( 0.813 to 1.068 ) |  | 390 ( 322 to 475 ) | 0.69 ( 0.571 to 0.842 ) | 1.041 ( 0.86 to 1.269 ) | -0.59 (-0.71 to -0.46) |
| Central African Republic | 8 ( 5 to 13 ) | 0.404 ( 0.241 to 0.755 ) | 0.293 ( 0.176 to 0.488 ) |  | 15 ( 8 to 25 ) | 0.389 ( 0.223 to 0.737 ) | 0.269 ( 0.154 to 0.452 ) | -0.11 (-0.21 to -0.01) |
| Chad | 18 ( 10 to 34 ) | 0.274 ( 0.167 to 0.442 ) | 0.299 ( 0.159 to 0.572 ) |  | 40 ( 20 to 77 ) | 0.224 ( 0.137 to 0.36 ) | 0.226 ( 0.112 to 0.432 ) | -0.68 (-0.79 to -0.57) |
| Chile | 44 ( 34 to 56 ) | 0.39 ( 0.302 to 0.488 ) | 0.334 ( 0.258 to 0.424 ) |  | 69 ( 56 to 84 ) | 0.334 ( 0.265 to 0.424 ) | 0.366 ( 0.295 to 0.445 ) | -0.23 (-0.62 to 0.16) |
| China | 1496 ( 937 to 2021 ) | 0.157 ( 0.099 to 0.21 ) | 0.127 ( 0.08 to 0.172 ) |  | 3729 ( 2065 to 4830 ) | 0.283 ( 0.149 to 0.391 ) | 0.262 ( 0.145 to 0.339 ) | 3.14 (2.74 to 3.53) |
| Colombia | 100 ( 78 to 130 ) | 0.388 ( 0.302 to 0.506 ) | 0.309 ( 0.239 to 0.401 ) |  | 186 ( 146 to 237 ) | 0.381 ( 0.296 to 0.493 ) | 0.379 ( 0.298 to 0.484 ) | 1.01 (0.69 to 1.34) |
| Comoros | 7 ( 5 to 11 ) | 2.324 ( 1.456 to 3.751 ) | 1.542 ( 0.973 to 2.392 ) |  | 15 ( 9 to 27 ) | 2.546 ( 1.603 to 4.589 ) | 2.035 ( 1.254 to 3.607 ) | 0.08 (-0.07 to 0.23) |
| Congo | 7 ( 4 to 11 ) | 0.404 ( 0.245 to 0.782 ) | 0.274 ( 0.179 to 0.466 ) |  | 15 ( 10 to 27 ) | 0.419 ( 0.255 to 0.776 ) | 0.284 ( 0.176 to 0.505 ) | 0.09 (-0.08 to 0.25) |
| Cook Islands | 0 ( 0 to 0 ) | 0.089 ( 0.052 to 0.155 ) | 0.078 ( 0.042 to 0.15 ) |  | 0 ( 0 to 0 ) | 0.356 ( 0.126 to 1.009 ) | 0.264 ( 0.11 to 0.684 ) | 2.03 (1.13 to 2.95) |
| Costa Rica | 14 ( 11 to 18 ) | 0.564 ( 0.462 to 0.691 ) | 0.456 ( 0.364 to 0.58 ) |  | 17 ( 13 to 21 ) | 0.35 ( 0.273 to 0.443 ) | 0.358 ( 0.284 to 0.446 ) | -1.06 (-1.52 to -0.59) |
| Coted'Ivoire | 16 ( 9 to 27 ) | 0.216 ( 0.138 to 0.331 ) | 0.134 ( 0.077 to 0.22 ) |  | 44 ( 23 to 72 ) | 0.248 ( 0.15 to 0.373 ) | 0.159 ( 0.082 to 0.259 ) | 0.63 (0.5 to 0.76) |
| Croatia | 48 ( 38 to 59 ) | 0.846 ( 0.67 to 1.049 ) | 0.986 ( 0.781 to 1.209 ) |  | 54 ( 40 to 73 ) | 0.666 ( 0.498 to 0.892 ) | 1.289 ( 0.948 to 1.742 ) | -0.49 (-0.75 to -0.23) |
| Cuba | 64 ( 51 to 78 ) | 0.623 ( 0.501 to 0.762 ) | 0.587 ( 0.474 to 0.72 ) |  | 101 ( 79 to 124 ) | 0.594 ( 0.463 to 0.736 ) | 0.896 ( 0.703 to 1.1 ) | 0.05 (-0.2 to 0.29) |
| Cyprus | 4 ( 3 to 6 ) | 0.512 ( 0.39 to 0.778 ) | 0.502 ( 0.384 to 0.759 ) |  | 11 ( 7 to 15 ) | 0.565 ( 0.36 to 0.748 ) | 0.784 ( 0.505 to 1.076 ) | 0.9 (0.68 to 1.11) |
| Czechia | 97 ( 72 to 125 ) | 0.754 ( 0.571 to 0.976 ) | 0.938 ( 0.703 to 1.213 ) |  | 120 ( 82 to 171 ) | 0.642 ( 0.456 to 0.883 ) | 1.124 ( 0.772 to 1.606 ) | -0.46 (-0.56 to -0.36) |
| Democratic People's Republic of Korea | 25 ( 16 to 37 ) | 0.133 ( 0.086 to 0.196 ) | 0.121 ( 0.078 to 0.179 ) |  | 56 ( 33 to 81 ) | 0.215 ( 0.126 to 0.335 ) | 0.211 ( 0.124 to 0.306 ) | 1.81 (1.67 to 1.95) |
| Democratic Republic of the Congo | 95 ( 59 to 150 ) | 0.36 ( 0.217 to 0.657 ) | 0.248 ( 0.155 to 0.392 ) |  | 220 ( 129 to 396 ) | 0.411 ( 0.229 to 0.797 ) | 0.244 ( 0.143 to 0.439 ) | 0.51 (0.35 to 0.67) |
| Denmark | 63 ( 52 to 74 ) | 0.997 ( 0.838 to 1.194 ) | 1.216 ( 1.003 to 1.443 ) |  | 99 ( 77 to 124 ) | 1.107 ( 0.891 to 1.373 ) | 1.684 ( 1.32 to 2.112 ) | 0.43 (0.27 to 0.59) |
| Djibouti | 5 ( 3 to 9 ) | 2.206 ( 1.379 to 3.657 ) | 1.319 ( 0.818 to 2.092 ) |  | 22 ( 12 to 40 ) | 2.479 ( 1.447 to 4.602 ) | 1.745 ( 0.967 to 3.197 ) | 0.34 (0.27 to 0.42) |
| Dominica | 0 ( 0 to 1 ) | 0.441 ( 0.228 to 0.804 ) | 0.391 ( 0.202 to 0.72 ) |  | 0 ( 0 to 1 ) | 0.54 ( 0.266 to 0.889 ) | 0.615 ( 0.309 to 1.007 ) | 0.77 (0.7 to 0.84) |
| Dominican Republic | 9 ( 5 to 14 ) | 0.149 ( 0.088 to 0.214 ) | 0.127 ( 0.069 to 0.2 ) |  | 16 ( 9 to 23 ) | 0.152 ( 0.09 to 0.219 ) | 0.145 ( 0.085 to 0.209 ) | 0.68 (0.44 to 0.91) |
| Ecuador | 29 ( 21 to 40 ) | 0.392 ( 0.291 to 0.532 ) | 0.295 ( 0.214 to 0.405 ) |  | 88 ( 65 to 117 ) | 0.54 ( 0.399 to 0.714 ) | 0.488 ( 0.36 to 0.646 ) | 1.35 (1.04 to 1.66) |
| Egypt | 31 ( 18 to 60 ) | 0.09 ( 0.056 to 0.181 ) | 0.056 ( 0.033 to 0.109 ) |  | 84 ( 51 to 159 ) | 0.114 ( 0.071 to 0.215 ) | 0.08 ( 0.048 to 0.15 ) | 0.91 (0.62 to 1.2) |
| El Salvador | 11 ( 7 to 16 ) | 0.25 ( 0.155 to 0.371 ) | 0.202 ( 0.123 to 0.3 ) |  | 22 ( 14 to 31 ) | 0.34 ( 0.219 to 0.485 ) | 0.342 ( 0.22 to 0.484 ) | 1.04 (0.91 to 1.17) |
| Equatorial Guinea | 1 ( 1 to 2 ) | 0.38 ( 0.223 to 0.69 ) | 0.284 ( 0.168 to 0.478 ) |  | 4 ( 2 to 6 ) | 0.407 ( 0.232 to 0.781 ) | 0.234 ( 0.13 to 0.427 ) | 0.19 (-0.06 to 0.43) |
| Eritrea | 51 ( 31 to 83 ) | 2.431 ( 1.525 to 4.012 ) | 1.49 ( 0.921 to 2.44 ) |  | 113 ( 64 to 201 ) | 2.639 ( 1.542 to 4.731 ) | 1.712 ( 0.977 to 3.039 ) | 0.18 (0.14 to 0.23) |
| Estonia | 13 ( 9 to 17 ) | 0.672 ( 0.492 to 0.914 ) | 0.82 ( 0.603 to 1.108 ) |  | 22 ( 16 to 31 ) | 0.984 ( 0.715 to 1.34 ) | 1.716 ( 1.235 to 2.327 ) | 0.3 (-0.05 to 0.65) |
| Eswatini | 3 ( 2 to 6 ) | 0.619 ( 0.333 to 1.41 ) | 0.374 ( 0.201 to 0.781 ) |  | 6 ( 3 to 13 ) | 0.715 ( 0.351 to 1.617 ) | 0.504 ( 0.244 to 1.113 ) | 0.67 (0.5 to 0.84) |
| Ethiopia | 389 ( 242 to 579 ) | 1.187 ( 0.7 to 1.857 ) | 0.769 ( 0.478 to 1.144 ) |  | 744 ( 484 to 1071 ) | 1.163 ( 0.703 to 1.752 ) | 0.683 ( 0.444 to 0.983 ) | -0.28 (-0.42 to -0.13) |
| Fiji | 1 ( 0 to 2 ) | 0.116 ( 0.05 to 0.301 ) | 0.081 ( 0.033 to 0.199 ) |  | 1 ( 0 to 3 ) | 0.122 ( 0.049 to 0.302 ) | 0.111 ( 0.043 to 0.277 ) | 0.11 (-0.12 to 0.34) |
| Finland | 46 ( 39 to 53 ) | 0.762 ( 0.644 to 0.879 ) | 0.918 ( 0.775 to 1.055 ) |  | 155 ( 125 to 195 ) | 1.758 ( 1.45 to 2.182 ) | 2.795 ( 2.255 to 3.515 ) | 2.72 (2.5 to 2.95) |
| France | 635 ( 560 to 710 ) | 0.946 ( 0.838 to 1.063 ) | 1.1 ( 0.969 to 1.229 ) |  | 1067 ( 833 to 1360 ) | 1.087 ( 0.879 to 1.347 ) | 1.608 ( 1.254 to 2.049 ) | 0.95 (0.77 to 1.14) |
| Gabon | 3 ( 2 to 5 ) | 0.382 ( 0.23 to 0.729 ) | 0.276 ( 0.171 to 0.476 ) |  | 6 ( 3 to 10 ) | 0.439 ( 0.265 to 0.862 ) | 0.313 ( 0.191 to 0.57 ) | 0.45 (0.36 to 0.55) |
| Gambia | 4 ( 2 to 7 ) | 0.595 ( 0.371 to 0.936 ) | 0.411 ( 0.246 to 0.677 ) |  | 12 ( 7 to 20 ) | 0.756 ( 0.461 to 1.212 ) | 0.51 ( 0.301 to 0.842 ) | 0.64 (0.44 to 0.84) |
| Georgia | 19 ( 15 to 23 ) | 0.319 ( 0.249 to 0.404 ) | 0.337 ( 0.265 to 0.425 ) |  | 40 ( 32 to 49 ) | 0.837 ( 0.653 to 1.085 ) | 1.105 ( 0.878 to 1.372 ) | 3.63 (3.2 to 4.06) |
| Germany | 680 ( 565 to 808 ) | 0.671 ( 0.555 to 0.799 ) | 0.851 ( 0.706 to 1.011 ) |  | 1490 ( 1210 to 1790 ) | 0.993 ( 0.816 to 1.186 ) | 1.746 ( 1.418 to 2.096 ) | 1.5 (1.41 to 1.6) |
| Ghana | 22 ( 9 to 51 ) | 0.103 ( 0.048 to 0.227 ) | 0.15 ( 0.061 to 0.343 ) |  | 42 ( 15 to 100 ) | 0.116 ( 0.041 to 0.252 ) | 0.122 ( 0.043 to 0.293 ) | 0.86 (0.56 to 1.16) |
| Greece | 73 ( 64 to 84 ) | 0.541 ( 0.475 to 0.621 ) | 0.703 ( 0.615 to 0.813 ) |  | 121 ( 102 to 141 ) | 0.637 ( 0.538 to 0.744 ) | 1.185 ( 1.006 to 1.387 ) | 0.85 (0.71 to 1) |
| Greenland | 0 ( 0 to 0 ) | 0.197 ( 0.084 to 0.295 ) | 0.155 ( 0.064 to 0.241 ) |  | 0 ( 0 to 0 ) | 0.111 ( 0.076 to 0.167 ) | 0.128 ( 0.083 to 0.182 ) | -1.13 (-1.52 to -0.73) |
| Grenada | 0 ( 0 to 0 ) | 0.19 ( 0.135 to 0.267 ) | 0.171 ( 0.119 to 0.246 ) |  | 0 ( 0 to 0 ) | 0.206 ( 0.156 to 0.272 ) | 0.219 ( 0.165 to 0.285 ) | 0.59 (0.28 to 0.9) |
| Guam | 0 ( 0 to 0 ) | 0.051 ( 0.026 to 0.101 ) | 0.043 ( 0.02 to 0.096 ) |  | 0 ( 0 to 0 ) | 0.054 ( 0.024 to 0.136 ) | 0.055 ( 0.025 to 0.119 ) | 1.73 (1.17 to 2.3) |
| Guatemala | 22 ( 15 to 32 ) | 0.369 ( 0.263 to 0.509 ) | 0.266 ( 0.173 to 0.383 ) |  | 46 ( 34 to 60 ) | 0.352 ( 0.265 to 0.457 ) | 0.291 ( 0.215 to 0.382 ) | 0.08 (-0.07 to 0.23) |
| Guinea | 30 ( 16 to 59 ) | 0.37 ( 0.221 to 0.636 ) | 0.502 ( 0.272 to 0.98 ) |  | 43 ( 21 to 89 ) | 0.315 ( 0.169 to 0.54 ) | 0.319 ( 0.155 to 0.661 ) | -0.29 (-0.41 to -0.17) |
| Guinea-Bissau | 4 ( 2 to 8 ) | 0.364 ( 0.206 to 0.624 ) | 0.369 ( 0.19 to 0.758 ) |  | 4 ( 2 to 7 ) | 0.246 ( 0.15 to 0.387 ) | 0.191 ( 0.104 to 0.348 ) | -1.04 (-1.17 to -0.91) |
| Guyana | 0 ( 0 to 0 ) | 0.043 ( 0.029 to 0.064 ) | 0.034 ( 0.022 to 0.053 ) |  | 2 ( 1 to 3 ) | 0.297 ( 0.197 to 0.43 ) | 0.27 ( 0.177 to 0.393 ) | 4.8 (2.96 to 6.68) |
| Haiti | 6 ( 3 to 11 ) | 0.12 ( 0.068 to 0.214 ) | 0.095 ( 0.05 to 0.178 ) |  | 13 ( 6 to 22 ) | 0.127 ( 0.066 to 0.202 ) | 0.099 ( 0.049 to 0.17 ) | 0.34 (0.29 to 0.39) |
| Honduras | 10 ( 7 to 16 ) | 0.291 ( 0.187 to 0.426 ) | 0.221 ( 0.144 to 0.336 ) |  | 25 ( 17 to 38 ) | 0.348 ( 0.232 to 0.506 ) | 0.251 ( 0.166 to 0.373 ) | 0.62 (0.55 to 0.69) |
| Hungary | 115 ( 92 to 145 ) | 0.878 ( 0.706 to 1.114 ) | 1.106 ( 0.885 to 1.394 ) |  | 123 ( 88 to 164 ) | 0.738 ( 0.538 to 0.998 ) | 1.278 ( 0.916 to 1.71 ) | -0.42 (-0.54 to -0.31) |
| Iceland | 2 ( 2 to 3 ) | 0.839 ( 0.725 to 0.966 ) | 0.863 ( 0.744 to 0.99 ) |  | 5 ( 4 to 6 ) | 1.059 ( 0.865 to 1.295 ) | 1.368 ( 1.087 to 1.735 ) | 0.91 (0.7 to 1.12) |
| India | 1032 ( 586 to 1439 ) | 0.12 ( 0.069 to 0.163 ) | 0.121 ( 0.069 to 0.169 ) |  | 1811 ( 1136 to 2436 ) | 0.15 ( 0.094 to 0.204 ) | 0.128 ( 0.08 to 0.172 ) | 0.75 (0.52 to 0.98) |
| Indonesia | 130 ( 68 to 190 ) | 0.089 ( 0.049 to 0.127 ) | 0.07 ( 0.037 to 0.103 ) |  | 304 ( 164 to 436 ) | 0.122 ( 0.066 to 0.173 ) | 0.109 ( 0.059 to 0.156 ) | 1.08 (1 to 1.16) |
| Iran (Islamic Republic of) | 3 ( 1 to 6 ) | 0.007 ( 0.001 to 0.012 ) | 0.005 ( 0.001 to 0.011 ) |  | 10 ( 3 to 16 ) | 0.014 ( 0.003 to 0.021 ) | 0.012 ( 0.003 to 0.018 ) | 3.42 (2.97 to 3.88) |
| Iraq | 20 ( 14 to 32 ) | 0.141 ( 0.1 to 0.204 ) | 0.111 ( 0.075 to 0.174 ) |  | 86 ( 53 to 129 ) | 0.247 ( 0.161 to 0.352 ) | 0.208 ( 0.13 to 0.313 ) | 1.98 (1.84 to 2.12) |
| Ireland | 39 ( 34 to 45 ) | 1.001 ( 0.869 to 1.158 ) | 1.079 ( 0.93 to 1.252 ) |  | 56 ( 43 to 71 ) | 0.812 ( 0.625 to 1.033 ) | 1.125 ( 0.863 to 1.441 ) | 0.07 (-0.15 to 0.29) |
| Israel | 16 ( 13 to 20 ) | 0.339 ( 0.273 to 0.412 ) | 0.332 ( 0.267 to 0.406 ) |  | 45 ( 37 to 55 ) | 0.413 ( 0.337 to 0.503 ) | 0.473 ( 0.385 to 0.575 ) | 1.05 (0.9 to 1.19) |
| Italy | 543 ( 488 to 605 ) | 0.772 ( 0.698 to 0.86 ) | 0.956 ( 0.859 to 1.065 ) |  | 1029 ( 851 to 1247 ) | 1.02 ( 0.872 to 1.186 ) | 1.721 ( 1.423 to 2.085 ) | 0.73 (0.5 to 0.97) |
| Jamaica | 5 ( 4 to 8 ) | 0.231 ( 0.162 to 0.328 ) | 0.222 ( 0.151 to 0.337 ) |  | 6 ( 4 to 9 ) | 0.207 ( 0.139 to 0.294 ) | 0.217 ( 0.149 to 0.306 ) | -0.2 (-0.67 to 0.28) |
| Japan | 275 ( 252 to 302 ) | 0.257 ( 0.229 to 0.287 ) | 0.218 ( 0.2 to 0.24 ) |  | 453 ( 390 to 521 ) | 0.276 ( 0.234 to 0.327 ) | 0.355 ( 0.306 to 0.408 ) | 0.75 (0.08 to 1.42) |
| Jordan | 4 ( 3 to 6 ) | 0.17 ( 0.119 to 0.252 ) | 0.116 ( 0.079 to 0.168 ) |  | 20 ( 13 to 29 ) | 0.218 ( 0.138 to 0.306 ) | 0.166 ( 0.106 to 0.237 ) | 1 (0.79 to 1.21) |
| Kazakhstan | 64 ( 42 to 95 ) | 0.426 ( 0.283 to 0.631 ) | 0.391 ( 0.254 to 0.577 ) |  | 68 ( 40 to 102 ) | 0.372 ( 0.218 to 0.558 ) | 0.359 ( 0.211 to 0.537 ) | -0.45 (-0.68 to -0.21) |
| Kenya | 465 ( 321 to 626 ) | 2.605 ( 1.74 to 3.703 ) | 2.01 ( 1.385 to 2.706 ) |  | 1116 ( 768 to 1583 ) | 3.259 ( 2.101 to 4.677 ) | 2.228 ( 1.533 to 3.163 ) | 0.89 (0.77 to 1) |
| Kiribati | 0 ( 0 to 0 ) | 0.126 ( 0.064 to 0.271 ) | 0.092 ( 0.044 to 0.182 ) |  | 0 ( 0 to 0 ) | 0.141 ( 0.068 to 0.337 ) | 0.106 ( 0.052 to 0.24 ) | 0.12 (-0.01 to 0.25) |
| Kuwait | 3 ( 2 to 4 ) | 0.227 ( 0.182 to 0.279 ) | 0.173 ( 0.131 to 0.232 ) |  | 5 ( 4 to 7 ) | 0.148 ( 0.116 to 0.186 ) | 0.11 ( 0.087 to 0.141 ) | 0.07 (-1.88 to 2.06) |
| Kyrgyzstan | 16 ( 11 to 23 ) | 0.444 ( 0.318 to 0.627 ) | 0.364 ( 0.255 to 0.526 ) |  | 33 ( 23 to 47 ) | 0.531 ( 0.371 to 0.747 ) | 0.475 ( 0.33 to 0.691 ) | 0.46 (-0.8 to 1.74) |
| Lao People's Democratic Republic | 3 ( 1 to 6 ) | 0.096 ( 0.045 to 0.159 ) | 0.08 ( 0.035 to 0.139 ) |  | 7 ( 3 to 11 ) | 0.11 ( 0.054 to 0.169 ) | 0.091 ( 0.044 to 0.144 ) | 0.49 (0.46 to 0.52) |
| Latvia | 16 ( 12 to 22 ) | 0.505 ( 0.37 to 0.663 ) | 0.62 ( 0.46 to 0.822 ) |  | 26 ( 19 to 35 ) | 0.777 ( 0.583 to 1.032 ) | 1.397 ( 1.037 to 1.869 ) | 0.83 (0.53 to 1.13) |
| Lebanon | 6 ( 5 to 11 ) | 0.27 ( 0.189 to 0.462 ) | 0.216 ( 0.153 to 0.359 ) |  | 21 ( 15 to 33 ) | 0.364 ( 0.254 to 0.553 ) | 0.382 ( 0.268 to 0.592 ) | 1.29 (1.17 to 1.41) |
| Lesotho | 6 ( 3 to 13 ) | 0.529 ( 0.266 to 1.211 ) | 0.385 ( 0.201 to 0.827 ) |  | 9 ( 5 to 21 ) | 0.673 ( 0.328 to 1.55 ) | 0.498 ( 0.244 to 1.122 ) | 1.08 (0.91 to 1.26) |
| Liberia | 6 ( 3 to 13 ) | 0.261 ( 0.136 to 0.446 ) | 0.262 ( 0.132 to 0.534 ) |  | 9 ( 5 to 19 ) | 0.225 ( 0.133 to 0.374 ) | 0.166 ( 0.089 to 0.345 ) | -0.54 (-0.91 to -0.17) |
| Libya | 6 ( 3 to 10 ) | 0.141 ( 0.09 to 0.222 ) | 0.132 ( 0.081 to 0.244 ) |  | 11 ( 7 to 17 ) | 0.203 ( 0.124 to 0.317 ) | 0.159 ( 0.097 to 0.242 ) | 1.19 (0.98 to 1.4) |
| Lithuania | 28 ( 22 to 37 ) | 0.67 ( 0.509 to 0.884 ) | 0.775 ( 0.59 to 1.017 ) |  | 49 ( 37 to 64 ) | 1.004 ( 0.755 to 1.334 ) | 1.794 ( 1.372 to 2.351 ) | 0.78 (0.52 to 1.04) |
| Luxembourg | 3 ( 3 to 4 ) | 0.649 ( 0.569 to 0.73 ) | 0.842 ( 0.741 to 0.951 ) |  | 6 ( 4 to 7 ) | 0.563 ( 0.45 to 0.699 ) | 0.861 ( 0.686 to 1.066 ) | 0.05 (-0.11 to 0.22) |
| Madagascar | 163 ( 111 to 254 ) | 2.049 ( 1.371 to 3.36 ) | 1.373 ( 0.929 to 2.13 ) |  | 353 ( 223 to 594 ) | 1.924 ( 1.237 to 3.387 ) | 1.236 ( 0.782 to 2.08 ) | -0.26 (-0.34 to -0.18) |
| Malawi | 363 ( 234 to 553 ) | 5.72 ( 3.607 to 8.549 ) | 3.705 ( 2.383 to 5.635 ) |  | 827 ( 457 to 1286 ) | 7.188 ( 4.039 to 11.241 ) | 4.251 ( 2.348 to 6.614 ) | 0.73 (0.65 to 0.81) |
| Malaysia | 19 ( 10 to 32 ) | 0.128 ( 0.07 to 0.201 ) | 0.108 ( 0.057 to 0.182 ) |  | 49 ( 27 to 71 ) | 0.174 ( 0.094 to 0.256 ) | 0.154 ( 0.084 to 0.223 ) | 1.21 (1.05 to 1.37) |
| Maldives | 0 ( 0 to 0 ) | 0.052 ( 0.031 to 0.084 ) | 0.035 ( 0.02 to 0.056 ) |  | 0 ( 0 to 0 ) | 0.059 ( 0.036 to 0.081 ) | 0.047 ( 0.028 to 0.069 ) | 0.56 (0.38 to 0.74) |
| Mali | 93 ( 59 to 144 ) | 1.42 ( 0.92 to 2.152 ) | 1.075 ( 0.68 to 1.661 ) |  | 195 ( 108 to 335 ) | 1.407 ( 0.835 to 2.292 ) | 0.811 ( 0.449 to 1.39 ) | 0.05 (-0.05 to 0.14) |
| Malta | 2 ( 2 to 2 ) | 0.514 ( 0.437 to 0.592 ) | 0.575 ( 0.491 to 0.663 ) |  | 5 ( 4 to 6 ) | 0.603 ( 0.465 to 0.774 ) | 1.076 ( 0.831 to 1.387 ) | 1.09 (0.93 to 1.26) |
| Marshall Islands | 0 ( 0 to 0 ) | 0.064 ( 0.039 to 0.103 ) | 0.043 ( 0.023 to 0.08 ) |  | 0 ( 0 to 0 ) | 0.076 ( 0.043 to 0.124 ) | 0.065 ( 0.034 to 0.109 ) | 0.53 (0.43 to 0.63) |
| Mauritania | 5 ( 3 to 11 ) | 0.274 ( 0.161 to 0.437 ) | 0.259 ( 0.144 to 0.516 ) |  | 10 ( 6 to 19 ) | 0.268 ( 0.174 to 0.426 ) | 0.235 ( 0.131 to 0.421 ) | -0.31 (-0.58 to -0.03) |
| Mauritius | 1 ( 0 to 1 ) | 0.062 ( 0.05 to 0.075 ) | 0.052 ( 0.041 to 0.063 ) |  | 3 ( 2 to 3 ) | 0.166 ( 0.138 to 0.197 ) | 0.197 ( 0.167 to 0.229 ) | 4.11 (3.07 to 5.17) |
| Mexico | 227 ( 179 to 287 ) | 0.331 ( 0.262 to 0.41 ) | 0.266 ( 0.209 to 0.337 ) |  | 382 ( 318 to 457 ) | 0.323 ( 0.266 to 0.387 ) | 0.296 ( 0.246 to 0.354 ) | -0.02 (-0.2 to 0.16) |
| Micronesia (Federated States of) | 0 ( 0 to 0 ) | 0.071 ( 0.044 to 0.125 ) | 0.054 ( 0.029 to 0.106 ) |  | 0 ( 0 to 0 ) | 0.083 ( 0.05 to 0.134 ) | 0.067 ( 0.04 to 0.11 ) | 0.78 (0.68 to 0.88) |
| Monaco | 0 ( 0 to 0 ) | 0.054 ( 0.032 to 0.076 ) | 0.082 ( 0.047 to 0.114 ) |  | 0 ( 0 to 0 ) | 0.09 ( 0.057 to 0.142 ) | 0.112 ( 0.074 to 0.152 ) | 0.8 (0.49 to 1.1) |
| Mongolia | 4 ( 2 to 6 ) | 0.24 ( 0.144 to 0.38 ) | 0.183 ( 0.106 to 0.301 ) |  | 10 ( 6 to 15 ) | 0.33 ( 0.203 to 0.486 ) | 0.296 ( 0.178 to 0.456 ) | 1.24 (1.08 to 1.39) |
| Montenegro | 2 ( 1 to 2 ) | 0.245 ( 0.163 to 0.319 ) | 0.242 ( 0.161 to 0.316 ) |  | 2 ( 2 to 3 ) | 0.279 ( 0.18 to 0.373 ) | 0.382 ( 0.25 to 0.514 ) | 0.57 (0.41 to 0.73) |
| Morocco | 62 ( 38 to 104 ) | 0.249 ( 0.16 to 0.373 ) | 0.246 ( 0.148 to 0.409 ) |  | 128 ( 83 to 196 ) | 0.377 ( 0.243 to 0.585 ) | 0.344 ( 0.223 to 0.527 ) | 1.63 (1.52 to 1.75) |
| Mozambique | 237 ( 160 to 351 ) | 2.438 ( 1.649 to 3.725 ) | 1.775 ( 1.195 to 2.624 ) |  | 509 ( 325 to 851 ) | 2.767 ( 1.677 to 4.931 ) | 1.639 ( 1.046 to 2.739 ) | 0.57 (0.5 to 0.64) |
| Myanmar | 37 ( 17 to 64 ) | 0.108 ( 0.053 to 0.183 ) | 0.091 ( 0.041 to 0.159 ) |  | 65 ( 34 to 103 ) | 0.126 ( 0.066 to 0.196 ) | 0.116 ( 0.061 to 0.182 ) | 0.39 (0.31 to 0.46) |
| Namibia | 22 ( 14 to 32 ) | 2.569 ( 1.585 to 3.802 ) | 1.56 ( 0.985 to 2.283 ) |  | 65 ( 36 to 100 ) | 3.709 ( 2.089 to 5.625 ) | 2.686 ( 1.494 to 4.118 ) | 1.18 (1.08 to 1.27) |
| Nauru | 0 ( 0 to 0 ) | 0.075 ( 0.045 to 0.129 ) | 0.062 ( 0.032 to 0.121 ) |  | 0 ( 0 to 0 ) | 0.098 ( 0.056 to 0.164 ) | 0.082 ( 0.042 to 0.157 ) | 0.68 (0.61 to 0.74) |
| Nepal | 30 ( 17 to 57 ) | 0.14 ( 0.083 to 0.248 ) | 0.154 ( 0.087 to 0.291 ) |  | 46 ( 25 to 83 ) | 0.164 ( 0.089 to 0.313 ) | 0.146 ( 0.079 to 0.268 ) | 0.78 (0.55 to 1.01) |
| Netherlands | 85 ( 74 to 97 ) | 0.563 ( 0.486 to 0.654 ) | 0.568 ( 0.497 to 0.653 ) |  | 113 ( 89 to 142 ) | 0.553 ( 0.422 to 0.692 ) | 0.656 ( 0.515 to 0.824 ) | 0.4 (0.14 to 0.65) |
| New Zealand | 43 ( 36 to 51 ) | 1.124 ( 0.938 to 1.337 ) | 1.252 ( 1.046 to 1.482 ) |  | 87 ( 69 to 110 ) | 1.132 ( 0.905 to 1.419 ) | 1.692 ( 1.338 to 2.125 ) | 0.33 (0.22 to 0.44) |
| Nicaragua | 12 ( 7 to 25 ) | 0.342 ( 0.223 to 0.543 ) | 0.317 ( 0.186 to 0.641 ) |  | 20 ( 14 to 28 ) | 0.347 ( 0.247 to 0.476 ) | 0.293 ( 0.212 to 0.413 ) | 0.56 (0.31 to 0.81) |
| Niger | 30 ( 15 to 67 ) | 0.324 ( 0.184 to 0.574 ) | 0.368 ( 0.184 to 0.832 ) |  | 51 ( 26 to 109 ) | 0.217 ( 0.131 to 0.357 ) | 0.205 ( 0.105 to 0.434 ) | -1.28 (-1.49 to -1.07) |
| Nigeria | 460 ( 236 to 660 ) | 0.723 ( 0.378 to 1.079 ) | 0.511 ( 0.262 to 0.733 ) |  | 1156 ( 511 to 1811 ) | 0.814 ( 0.418 to 1.202 ) | 0.5 ( 0.221 to 0.783 ) | 0.36 (0.26 to 0.45) |
| Niue | 0 ( 0 to 0 ) | 0.096 ( 0.054 to 0.175 ) | 0.095 ( 0.053 to 0.187 ) |  | 0 ( 0 to 0 ) | 0.291 ( 0.115 to 0.843 ) | 0.239 ( 0.108 to 0.644 ) | 1.66 (0.94 to 2.39) |
| North Macedonia | 9 ( 6 to 13 ) | 0.5 ( 0.321 to 0.69 ) | 0.47 ( 0.305 to 0.656 ) |  | 16 ( 11 to 23 ) | 0.563 ( 0.366 to 0.779 ) | 0.742 ( 0.487 to 1.049 ) | 0.43 (0.15 to 0.7) |
| Northern Mariana Islands | 0 ( 0 to 0 ) | 0.087 ( 0.046 to 0.132 ) | 0.071 ( 0.034 to 0.121 ) |  | 0 ( 0 to 0 ) | 0.106 ( 0.053 to 0.152 ) | 0.107 ( 0.053 to 0.153 ) | 0.65 (0.02 to 1.29) |
| Norway | 55 ( 49 to 61 ) | 0.919 ( 0.83 to 1.014 ) | 1.298 ( 1.164 to 1.437 ) |  | 24 ( 20 to 30 ) | 0.266 ( 0.218 to 0.33 ) | 0.442 ( 0.361 to 0.552 ) | -4.38 (-4.9 to -3.87) |
| Oman | 2 ( 1 to 3 ) | 0.171 ( 0.071 to 0.272 ) | 0.104 ( 0.047 to 0.175 ) |  | 6 ( 2 to 9 ) | 0.193 ( 0.076 to 0.294 ) | 0.121 ( 0.049 to 0.193 ) | 0.72 (0.48 to 0.96) |
| Pakistan | 281 ( 183 to 412 ) | 0.335 ( 0.214 to 0.534 ) | 0.253 ( 0.165 to 0.371 ) |  | 825 ( 502 to 1198 ) | 0.449 ( 0.278 to 0.668 ) | 0.35 ( 0.213 to 0.509 ) | 0.92 (0.87 to 0.98) |
| Palau | 0 ( 0 to 0 ) | 0.046 ( 0.023 to 0.088 ) | 0.04 ( 0.019 to 0.08 ) |  | 0 ( 0 to 0 ) | 0.057 ( 0.026 to 0.136 ) | 0.047 ( 0.022 to 0.092 ) | 0.69 (0.59 to 0.78) |
| Palestine | 1 ( 1 to 2 ) | 0.092 ( 0.051 to 0.136 ) | 0.057 ( 0.03 to 0.087 ) |  | 4 ( 2 to 5 ) | 0.108 ( 0.056 to 0.146 ) | 0.071 ( 0.036 to 0.099 ) | 0.69 (0.49 to 0.88) |
| Panama | 6 ( 4 to 9 ) | 0.287 ( 0.215 to 0.389 ) | 0.255 ( 0.183 to 0.369 ) |  | 14 ( 10 to 19 ) | 0.333 ( 0.245 to 0.456 ) | 0.33 ( 0.244 to 0.442 ) | 0.62 (0.55 to 0.68) |
| Papua New Guinea | 2 ( 1 to 4 ) | 0.06 ( 0.035 to 0.097 ) | 0.047 ( 0.024 to 0.097 ) |  | 6 ( 3 to 13 ) | 0.071 ( 0.039 to 0.126 ) | 0.061 ( 0.031 to 0.122 ) | 0.48 (0.4 to 0.55) |
| Paraguay | 11 ( 7 to 17 ) | 0.328 ( 0.215 to 0.49 ) | 0.281 ( 0.169 to 0.425 ) |  | 29 ( 18 to 44 ) | 0.456 ( 0.295 to 0.693 ) | 0.402 ( 0.257 to 0.616 ) | 1.35 (1.21 to 1.48) |
| Peru | 64 ( 42 to 100 ) | 0.326 ( 0.219 to 0.49 ) | 0.298 ( 0.195 to 0.461 ) |  | 148 ( 97 to 223 ) | 0.438 ( 0.285 to 0.663 ) | 0.408 ( 0.267 to 0.615 ) | 1.56 (1.35 to 1.76) |
| Philippines | 114 ( 58 to 165 ) | 0.218 ( 0.117 to 0.303 ) | 0.181 ( 0.092 to 0.261 ) |  | 251 ( 129 to 340 ) | 0.25 ( 0.133 to 0.339 ) | 0.222 ( 0.114 to 0.3 ) | 0.59 (0.5 to 0.67) |
| Poland | 393 ( 327 to 464 ) | 0.957 ( 0.794 to 1.132 ) | 1.03 ( 0.857 to 1.216 ) |  | 493 ( 427 to 562 ) | 0.768 ( 0.667 to 0.88 ) | 1.29 ( 1.116 to 1.469 ) | -0.91 (-1.19 to -0.63) |
| Portugal | 93 ( 79 to 108 ) | 0.911 ( 0.766 to 1.073 ) | 0.914 ( 0.777 to 1.068 ) |  | 141 ( 118 to 172 ) | 1.035 ( 0.852 to 1.215 ) | 1.326 ( 1.114 to 1.619 ) | 0.8 (0.36 to 1.25) |
| Puerto Rico | 6 ( 5 to 8 ) | 0.172 ( 0.137 to 0.216 ) | 0.168 ( 0.132 to 0.211 ) |  | 10 ( 8 to 13 ) | 0.181 ( 0.142 to 0.226 ) | 0.308 ( 0.24 to 0.382 ) | 0.47 (-0.28 to 1.24) |
| Qatar | 0 ( 0 to 1 ) | 0.182 ( 0.113 to 0.295 ) | 0.07 ( 0.043 to 0.116 ) |  | 3 ( 1 to 5 ) | 0.22 ( 0.105 to 0.347 ) | 0.089 ( 0.045 to 0.164 ) | 1.29 (0.75 to 1.84) |
| Republic of Korea | 87 ( 51 to 124 ) | 0.239 ( 0.142 to 0.334 ) | 0.197 ( 0.116 to 0.279 ) |  | 150 ( 104 to 194 ) | 0.415 ( 0.297 to 0.563 ) | 0.291 ( 0.202 to 0.377 ) | 2.19 (1.94 to 2.44) |
| Republic of Moldova | 22 ( 17 to 31 ) | 0.505 ( 0.374 to 0.711 ) | 0.503 ( 0.374 to 0.705 ) |  | 23 ( 19 to 28 ) | 0.466 ( 0.37 to 0.574 ) | 0.652 ( 0.533 to 0.792 ) | -0.31 (-0.54 to -0.08) |
| Romania | 155 ( 108 to 211 ) | 0.634 ( 0.434 to 0.874 ) | 0.664 ( 0.463 to 0.903 ) |  | 215 ( 167 to 275 ) | 0.724 ( 0.561 to 0.924 ) | 1.137 ( 0.88 to 1.452 ) | 0.2 (0.02 to 0.38) |
| Russian Federation | 870 ( 642 to 1124 ) | 0.529 ( 0.393 to 0.685 ) | 0.576 ( 0.425 to 0.745 ) |  | 1252 ( 1079 to 1423 ) | 0.611 ( 0.526 to 0.693 ) | 0.864 ( 0.745 to 0.982 ) | 0.24 (0.03 to 0.45) |
| Rwanda | 122 ( 81 to 189 ) | 2.528 ( 1.677 to 4.13 ) | 1.699 ( 1.126 to 2.629 ) |  | 216 ( 131 to 385 ) | 2.437 ( 1.479 to 4.467 ) | 1.624 ( 0.99 to 2.898 ) | -0.45 (-0.6 to -0.3) |
| Saint Kitts and Nevis | 0 ( 0 to 0 ) | 0.042 ( 0.029 to 0.059 ) | 0.04 ( 0.027 to 0.057 ) |  | 0 ( 0 to 0 ) | 0.032 ( 0.024 to 0.041 ) | 0.031 ( 0.023 to 0.039 ) | -0.81 (-1.01 to -0.61) |
| Saint Lucia | 0 ( 0 to 0 ) | 0.047 ( 0.036 to 0.06 ) | 0.036 ( 0.027 to 0.047 ) |  | 0 ( 0 to 0 ) | 0.049 ( 0.037 to 0.062 ) | 0.056 ( 0.043 to 0.07 ) | 0.1 (-0.2 to 0.4) |
| Saint Vincent and the Grenadines | 1 ( 1 to 1 ) | 0.892 ( 0.665 to 1.221 ) | 0.831 ( 0.598 to 1.195 ) |  | 0 ( 0 to 1 ) | 0.39 ( 0.295 to 0.487 ) | 0.41 ( 0.315 to 0.51 ) | -2.22 (-2.67 to -1.77) |
| Samoa | 1 ( 0 to 1 ) | 0.531 ( 0.269 to 1.449 ) | 0.325 ( 0.172 to 0.802 ) |  | 1 ( 1 to 2 ) | 0.639 ( 0.331 to 1.507 ) | 0.493 ( 0.26 to 1.101 ) | 0.6 (0.56 to 0.64) |
| San Marino | 0 ( 0 to 0 ) | 0.552 ( 0.372 to 0.761 ) | 0.721 ( 0.494 to 1.002 ) |  | 0 ( 0 to 0 ) | 0.378 ( 0.219 to 0.58 ) | 0.67 ( 0.386 to 1.053 ) | -0.38 (-0.69 to -0.07) |
| Sao Tome and Principe | 0 ( 0 to 1 ) | 0.287 ( 0.161 to 0.49 ) | 0.283 ( 0.148 to 0.566 ) |  | 1 ( 0 to 1 ) | 0.297 ( 0.182 to 0.49 ) | 0.238 ( 0.126 to 0.449 ) | 0.18 (-0.28 to 0.66) |
| Saudi Arabia | 14 ( 5 to 24 ) | 0.127 ( 0.05 to 0.203 ) | 0.09 ( 0.03 to 0.154 ) |  | 54 ( 20 to 85 ) | 0.193 ( 0.072 to 0.295 ) | 0.144 ( 0.053 to 0.226 ) | 1.79 (1.31 to 2.26) |
| Senegal | 25 ( 13 to 52 ) | 0.322 ( 0.177 to 0.537 ) | 0.333 ( 0.168 to 0.679 ) |  | 37 ( 20 to 68 ) | 0.271 ( 0.166 to 0.433 ) | 0.234 ( 0.126 to 0.43 ) | -0.45 (-0.72 to -0.17) |
| Serbia | 61 ( 39 to 85 ) | 0.614 ( 0.4 to 0.85 ) | 0.635 ( 0.407 to 0.881 ) |  | 95 ( 58 to 126 ) | 0.635 ( 0.395 to 0.846 ) | 1.067 ( 0.645 to 1.414 ) | 0.04 (-0.07 to 0.16) |
| Seychelles | 0 ( 0 to 0 ) | 0.184 ( 0.111 to 0.261 ) | 0.151 ( 0.095 to 0.213 ) |  | 0 ( 0 to 0 ) | 0.168 ( 0.097 to 0.227 ) | 0.184 ( 0.103 to 0.249 ) | 0.03 (-0.09 to 0.14) |
| Sierra Leone | 12 ( 6 to 21 ) | 0.272 ( 0.148 to 0.449 ) | 0.281 ( 0.141 to 0.515 ) |  | 16 ( 8 to 32 ) | 0.217 ( 0.127 to 0.357 ) | 0.182 ( 0.096 to 0.363 ) | -0.71 (-0.91 to -0.5) |
| Singapore | 11 ( 8 to 13 ) | 0.483 ( 0.375 to 0.587 ) | 0.346 ( 0.274 to 0.419 ) |  | 19 ( 15 to 24 ) | 0.485 ( 0.343 to 0.628 ) | 0.34 ( 0.263 to 0.428 ) | 0.26 (-0.16 to 0.68) |
| Slovakia | 42 ( 28 to 57 ) | 0.721 ( 0.493 to 0.991 ) | 0.79 ( 0.53 to 1.081 ) |  | 60 ( 39 to 83 ) | 0.729 ( 0.473 to 0.983 ) | 1.11 ( 0.725 to 1.533 ) | 0.13 (0.06 to 0.2) |
| Slovenia | 13 ( 10 to 16 ) | 0.545 ( 0.425 to 0.684 ) | 0.644 ( 0.501 to 0.812 ) |  | 16 ( 12 to 22 ) | 0.455 ( 0.336 to 0.604 ) | 0.797 ( 0.595 to 1.073 ) | -0.27 (-0.45 to -0.09) |
| Solomon Islands | 0 ( 0 to 0 ) | 0.056 ( 0.029 to 0.096 ) | 0.042 ( 0.019 to 0.097 ) |  | 0 ( 0 to 1 ) | 0.079 ( 0.047 to 0.135 ) | 0.065 ( 0.035 to 0.127 ) | 1.12 (0.93 to 1.31) |
| Somalia | 114 ( 70 to 186 ) | 2.491 ( 1.548 to 4.288 ) | 1.432 ( 0.877 to 2.348 ) |  | 269 ( 145 to 467 ) | 2.448 ( 1.368 to 4.331 ) | 1.247 ( 0.67 to 2.163 ) | -0.07 (-0.14 to 0) |
| South Africa | 74 ( 49 to 103 ) | 0.249 ( 0.161 to 0.361 ) | 0.2 ( 0.132 to 0.277 ) |  | 181 ( 105 to 256 ) | 0.356 ( 0.203 to 0.494 ) | 0.319 ( 0.184 to 0.45 ) | 1.38 (1.21 to 1.55) |
| South Sudan | 78 ( 50 to 130 ) | 2.073 ( 1.297 to 3.688 ) | 1.331 ( 0.852 to 2.209 ) |  | 138 ( 83 to 245 ) | 2.386 ( 1.406 to 4.32 ) | 1.424 ( 0.857 to 2.529 ) | 0.33 (0.15 to 0.51) |
| Spain | 298 ( 265 to 336 ) | 0.7 ( 0.618 to 0.801 ) | 0.769 ( 0.684 to 0.868 ) |  | 528 ( 423 to 655 ) | 0.82 ( 0.643 to 1 ) | 1.159 ( 0.928 to 1.437 ) | 0.87 (0.61 to 1.13) |
| Sri Lanka | 25 ( 18 to 36 ) | 0.183 ( 0.13 to 0.255 ) | 0.148 ( 0.105 to 0.21 ) |  | 50 ( 29 to 73 ) | 0.21 ( 0.125 to 0.307 ) | 0.222 ( 0.131 to 0.328 ) | 0.86 (0.65 to 1.07) |
| Sudan | 2 ( 0 to 6 ) | 0.013 ( 0.002 to 0.049 ) | 0.009 ( 0.002 to 0.032 ) |  | 6 ( 1 to 19 ) | 0.02 ( 0.004 to 0.069 ) | 0.013 ( 0.003 to 0.045 ) | 1.63 (1.44 to 1.82) |
| Suriname | 1 ( 0 to 1 ) | 0.202 ( 0.124 to 0.304 ) | 0.171 ( 0.097 to 0.273 ) |  | 1 ( 1 to 2 ) | 0.245 ( 0.141 to 0.377 ) | 0.251 ( 0.146 to 0.385 ) | 1.02 (0.79 to 1.26) |
| Sweden | 50 ( 42 to 59 ) | 0.38 ( 0.323 to 0.452 ) | 0.579 ( 0.493 to 0.685 ) |  | 125 ( 96 to 160 ) | 0.65 ( 0.502 to 0.828 ) | 1.206 ( 0.927 to 1.543 ) | 2.51 (2.02 to 2.99) |
| Switzerland | 59 ( 50 to 69 ) | 0.75 ( 0.635 to 0.874 ) | 0.858 ( 0.723 to 1.003 ) |  | 153 ( 116 to 194 ) | 1.199 ( 0.958 to 1.486 ) | 1.71 ( 1.303 to 2.175 ) | 1.4 (1.15 to 1.64) |
| Syrian Arab Republic | 2 ( 1 to 4 ) | 0.032 ( 0.02 to 0.05 ) | 0.019 ( 0.012 to 0.029 ) |  | 6 ( 4 to 9 ) | 0.044 ( 0.027 to 0.065 ) | 0.041 ( 0.025 to 0.063 ) | 1.02 (0.94 to 1.1) |
| Taiwan (Province of China) | 38 ( 33 to 44 ) | 0.221 ( 0.192 to 0.255 ) | 0.186 ( 0.16 to 0.215 ) |  | 57 ( 46 to 71 ) | 0.252 ( 0.189 to 0.341 ) | 0.242 ( 0.196 to 0.299 ) | 1.63 (1.11 to 2.15) |
| Tajikistan | 1 ( 0 to 3 ) | 0.014 ( 0.005 to 0.032 ) | 0.021 ( 0.006 to 0.051 ) |  | 3 ( 1 to 9 ) | 0.026 ( 0.007 to 0.069 ) | 0.032 ( 0.007 to 0.088 ) | 2.38 (2.16 to 2.6) |
| Thailand | 98 ( 61 to 134 ) | 0.207 ( 0.132 to 0.282 ) | 0.172 ( 0.108 to 0.236 ) |  | 197 ( 120 to 275 ) | 0.256 ( 0.173 to 0.347 ) | 0.296 ( 0.18 to 0.413 ) | 0.55 (0.33 to 0.77) |
| Timor-Leste | 0 ( 0 to 1 ) | 0.079 ( 0.041 to 0.12 ) | 0.061 ( 0.028 to 0.102 ) |  | 1 ( 1 to 2 ) | 0.111 ( 0.056 to 0.174 ) | 0.092 ( 0.045 to 0.15 ) | 1.39 (1.15 to 1.62) |
| Togo | 9 ( 5 to 18 ) | 0.294 ( 0.169 to 0.477 ) | 0.259 ( 0.14 to 0.5 ) |  | 16 ( 9 to 31 ) | 0.25 ( 0.153 to 0.409 ) | 0.191 ( 0.106 to 0.375 ) | -0.61 (-0.84 to -0.38) |
| Tokelau | 0 ( 0 to 0 ) | 0.077 ( 0.048 to 0.128 ) | 0.068 ( 0.042 to 0.112 ) |  | 0 ( 0 to 0 ) | 1.266 ( 0.329 to 3.921 ) | 0.991 ( 0.269 to 3.026 ) | 3.41 (1.15 to 5.71) |
| Tonga | 0 ( 0 to 0 ) | 0.071 ( 0.042 to 0.123 ) | 0.056 ( 0.03 to 0.117 ) |  | 0 ( 0 to 0 ) | 0.093 ( 0.05 to 0.167 ) | 0.09 ( 0.046 to 0.189 ) | 0.86 (0.76 to 0.96) |
| Trinidad and Tobago | 2 ( 2 to 3 ) | 0.197 ( 0.144 to 0.269 ) | 0.178 ( 0.126 to 0.255 ) |  | 3 ( 2 to 4 ) | 0.241 ( 0.154 to 0.364 ) | 0.23 ( 0.158 to 0.321 ) | 1.39 (1.03 to 1.75) |
| Tunisia | 17 ( 11 to 27 ) | 0.23 ( 0.159 to 0.351 ) | 0.204 ( 0.138 to 0.328 ) |  | 33 ( 21 to 49 ) | 0.282 ( 0.182 to 0.434 ) | 0.275 ( 0.178 to 0.412 ) | 0.6 (0.47 to 0.73) |
| Turkey | 79 ( 55 to 120 ) | 0.174 ( 0.122 to 0.276 ) | 0.137 ( 0.096 to 0.21 ) |  | 212 ( 149 to 286 ) | 0.267 ( 0.183 to 0.36 ) | 0.254 ( 0.178 to 0.342 ) | 1.69 (1.57 to 1.82) |
| Turkmenistan | 10 ( 6 to 14 ) | 0.339 ( 0.223 to 0.506 ) | 0.259 ( 0.167 to 0.388 ) |  | 17 ( 10 to 27 ) | 0.358 ( 0.213 to 0.574 ) | 0.321 ( 0.187 to 0.52 ) | -0.09 (-0.2 to 0.03) |
| Tuvalu | 0 ( 0 to 0 ) | 0.08 ( 0.046 to 0.136 ) | 0.073 ( 0.04 to 0.137 ) |  | 0 ( 0 to 0 ) | 0.087 ( 0.055 to 0.149 ) | 0.078 ( 0.048 to 0.137 ) | 0.34 (0.22 to 0.46) |
| Uganda | 304 ( 182 to 446 ) | 3.124 ( 1.835 to 4.874 ) | 1.756 ( 1.051 to 2.582 ) |  | 893 ( 518 to 1348 ) | 3.846 ( 2.266 to 5.83 ) | 2.063 ( 1.197 to 3.112 ) | 0.38 (0.27 to 0.5) |
| Ukraine | 321 ( 258 to 409 ) | 0.483 ( 0.389 to 0.616 ) | 0.61 ( 0.489 to 0.776 ) |  | 340 ( 237 to 470 ) | 0.535 ( 0.38 to 0.726 ) | 0.789 ( 0.55 to 1.092 ) | 0.38 (0.23 to 0.54) |
| United Arab Emirates | 2 ( 1 to 4 ) | 0.306 ( 0.181 to 0.539 ) | 0.126 ( 0.075 to 0.214 ) |  | 13 ( 9 to 25 ) | 0.393 ( 0.277 to 0.647 ) | 0.135 ( 0.092 to 0.256 ) | 2.69 (2.06 to 3.32) |
| United Kingdom | 735 ( 676 to 794 ) | 0.93 ( 0.861 to 1.006 ) | 1.282 ( 1.179 to 1.385 ) |  | 756 ( 669 to 853 ) | 0.712 ( 0.636 to 0.798 ) | 1.114 ( 0.986 to 1.257 ) | -0.36 (-0.74 to 0.02) |
| United Republic of Tanzania | 425 ( 288 to 647 ) | 2.488 ( 1.622 to 3.952 ) | 1.644 ( 1.114 to 2.505 ) |  | 905 ( 550 to 1571 ) | 2.327 ( 1.443 to 4.039 ) | 1.548 ( 0.941 to 2.688 ) | -0.34 (-0.38 to -0.29) |
| United States of America | 1707 ( 1574 to 1856 ) | 0.64 ( 0.587 to 0.696 ) | 0.672 ( 0.62 to 0.73 ) |  | 2405 ( 2141 to 2667 ) | 0.537 ( 0.479 to 0.602 ) | 0.723 ( 0.644 to 0.802 ) | -0.39 (-0.6 to -0.17) |
| United States Virgin Islands | 0 ( 0 to 0 ) | 0.127 ( 0.07 to 0.234 ) | 0.123 ( 0.066 to 0.232 ) |  | 0 ( 0 to 0 ) | 0.097 ( 0.059 to 0.172 ) | 0.083 ( 0.056 to 0.125 ) | -0.92 (-1.29 to -0.54) |
| Uruguay | 15 ( 12 to 20 ) | 0.464 ( 0.345 to 0.61 ) | 0.491 ( 0.371 to 0.626 ) |  | 20 ( 16 to 25 ) | 0.49 ( 0.391 to 0.619 ) | 0.589 ( 0.476 to 0.727 ) | -0.02 (-0.21 to 0.18) |
| Uzbekistan | 31 ( 18 to 49 ) | 0.176 ( 0.107 to 0.279 ) | 0.147 ( 0.086 to 0.235 ) |  | 78 ( 52 to 112 ) | 0.25 ( 0.166 to 0.36 ) | 0.227 ( 0.151 to 0.328 ) | 1.26 (0.92 to 1.61) |
| Vanuatu | 0 ( 0 to 0 ) | 0.056 ( 0.033 to 0.093 ) | 0.041 ( 0.021 to 0.079 ) |  | 0 ( 0 to 0 ) | 0.069 ( 0.042 to 0.116 ) | 0.054 ( 0.031 to 0.09 ) | 0.54 (0.37 to 0.71) |
| Venezuela (Bolivarian Republic of) | 55 ( 41 to 74 ) | 0.382 ( 0.292 to 0.495 ) | 0.292 ( 0.215 to 0.393 ) |  | 120 ( 85 to 160 ) | 0.441 ( 0.31 to 0.593 ) | 0.449 ( 0.317 to 0.6 ) | 0.5 (0.35 to 0.64) |
| Viet Nam | 148 ( 88 to 255 ) | 0.21 ( 0.131 to 0.332 ) | 0.217 ( 0.129 to 0.374 ) |  | 303 ( 196 to 458 ) | 0.331 ( 0.215 to 0.516 ) | 0.302 ( 0.195 to 0.456 ) | 1.51 (1.4 to 1.63) |
| Yemen | 1 ( 0 to 3 ) | 0.013 ( 0.002 to 0.046 ) | 0.007 ( 0.001 to 0.024 ) |  | 4 ( 1 to 12 ) | 0.019 ( 0.004 to 0.063 ) | 0.011 ( 0.002 to 0.035 ) | 1.65 (1.46 to 1.84) |
| Zambia | 116 ( 76 to 182 ) | 2.249 ( 1.465 to 3.678 ) | 1.461 ( 0.959 to 2.298 ) |  | 420 ( 191 to 768 ) | 3.668 ( 1.749 to 6.732 ) | 2.154 ( 0.977 to 3.934 ) | 1.86 (1.55 to 2.17) |
| Zimbabwe | 74 ( 44 to 108 ) | 1.339 ( 0.807 to 1.961 ) | 0.713 ( 0.425 to 1.045 ) |  | 235 ( 100 to 389 ) | 2.386 ( 1.007 to 3.97 ) | 1.506 ( 0.642 to 2.493 ) | 2.7 (1.86 to 3.54) |

**Supplementary Table S3: Mortality of Eye Cancer in 204 Countries and Territories in 1990 and 2021, with EAPC**

| **Location** | **1990** | | |  | **2021** | | | **EAPC(95%CI)** |
| --- | --- | --- | --- | --- | --- | --- | --- | --- |
|  | **Number(95%UI)** | **ASR(95%UI)** | **Rate(95%UI)** |  | **Number(95%UI)** | **ASR(95%UI)** | **Rate(95%UI)** |  |
| Afghanistan | 1 ( 0 to 3 ) | 0.011 ( 0.002 to 0.043 ) | 0.008 ( 0.001 to 0.032 ) |  | 2 ( 0 to 6 ) | 0.013 ( 0.002 to 0.045 ) | 0.005 ( 0.001 to 0.018 ) | 0.47 (0.39 to 0.56) |
| Albania | 9 ( 6 to 12 ) | 0.455 ( 0.317 to 0.638 ) | 0.264 ( 0.184 to 0.369 ) |  | 13 ( 8 to 20 ) | 0.306 ( 0.204 to 0.479 ) | 0.477 ( 0.315 to 0.747 ) | -1.14 (-1.28 to -1) |
| Algeria | 13 ( 8 to 20 ) | 0.069 ( 0.043 to 0.096 ) | 0.052 ( 0.033 to 0.081 ) |  | 16 ( 10 to 22 ) | 0.045 ( 0.028 to 0.064 ) | 0.036 ( 0.023 to 0.05 ) | -1.33 (-1.37 to -1.29) |
| American Samoa | 0 ( 0 to 0 ) | 0.022 ( 0.014 to 0.032 ) | 0.014 ( 0.009 to 0.023 ) |  | 0 ( 0 to 0 ) | 0.022 ( 0.013 to 0.032 ) | 0.02 ( 0.011 to 0.029 ) | 0.39 (-0.1 to 0.89) |
| Andorra | 0 ( 0 to 0 ) | 0.116 ( 0.064 to 0.177 ) | 0.121 ( 0.067 to 0.186 ) |  | 0 ( 0 to 0 ) | 0.07 ( 0.037 to 0.105 ) | 0.126 ( 0.067 to 0.187 ) | -1.36 (-1.56 to -1.17) |
| Angola | 18 ( 12 to 31 ) | 0.247 ( 0.172 to 0.403 ) | 0.176 ( 0.113 to 0.3 ) |  | 39 ( 23 to 63 ) | 0.204 ( 0.13 to 0.357 ) | 0.119 ( 0.071 to 0.193 ) | -0.68 (-0.74 to -0.61) |
| Antigua and Barbuda | 0 ( 0 to 0 ) | 0.091 ( 0.079 to 0.102 ) | 0.084 ( 0.074 to 0.095 ) |  | 0 ( 0 to 0 ) | 0.134 ( 0.119 to 0.149 ) | 0.147 ( 0.129 to 0.164 ) | 1.99 (1.27 to 2.71) |
| Argentina | 32 ( 26 to 38 ) | 0.101 ( 0.083 to 0.121 ) | 0.096 ( 0.079 to 0.114 ) |  | 32 ( 27 to 39 ) | 0.059 ( 0.049 to 0.071 ) | 0.071 ( 0.059 to 0.087 ) | -1.33 (-1.49 to -1.17) |
| Armenia | 3 ( 2 to 4 ) | 0.117 ( 0.077 to 0.173 ) | 0.089 ( 0.059 to 0.128 ) |  | 5 ( 3 to 7 ) | 0.119 ( 0.077 to 0.179 ) | 0.165 ( 0.108 to 0.243 ) | -0.03 (-0.13 to 0.06) |
| Australia | 32 ( 29 to 35 ) | 0.164 ( 0.147 to 0.182 ) | 0.189 ( 0.169 to 0.21 ) |  | 48 ( 38 to 58 ) | 0.106 ( 0.086 to 0.129 ) | 0.184 ( 0.149 to 0.223 ) | -1.53 (-1.66 to -1.39) |
| Austria | 16 ( 14 to 18 ) | 0.145 ( 0.127 to 0.162 ) | 0.212 ( 0.185 to 0.237 ) |  | 22 ( 18 to 27 ) | 0.124 ( 0.101 to 0.149 ) | 0.248 ( 0.198 to 0.304 ) | 0.13 (-0.17 to 0.44) |
| Azerbaijan | 5 ( 3 to 9 ) | 0.079 ( 0.045 to 0.139 ) | 0.062 ( 0.034 to 0.123 ) |  | 5 ( 3 to 9 ) | 0.058 ( 0.033 to 0.103 ) | 0.05 ( 0.028 to 0.088 ) | -0.94 (-1.06 to -0.81) |
| Bahamas | 0 ( 0 to 0 ) | 0.187 ( 0.161 to 0.218 ) | 0.13 ( 0.111 to 0.155 ) |  | 1 ( 0 to 1 ) | 0.144 ( 0.114 to 0.179 ) | 0.137 ( 0.11 to 0.171 ) | -0.72 (-1.06 to -0.38) |
| Bahrain | 0 ( 0 to 0 ) | 0.019 ( 0.013 to 0.035 ) | 0.007 ( 0.005 to 0.013 ) |  | 0 ( 0 to 0 ) | 0.021 ( 0.009 to 0.033 ) | 0.01 ( 0.004 to 0.017 ) | 0.69 (0.43 to 0.95) |
| Bangladesh | 163 ( 85 to 304 ) | 0.126 ( 0.073 to 0.219 ) | 0.149 ( 0.078 to 0.279 ) |  | 106 ( 63 to 183 ) | 0.074 ( 0.044 to 0.129 ) | 0.064 ( 0.038 to 0.111 ) | -1.81 (-1.89 to -1.72) |
| Barbados | 0 ( 0 to 0 ) | 0.067 ( 0.048 to 0.105 ) | 0.067 ( 0.051 to 0.096 ) |  | 0 ( 0 to 0 ) | 0.064 ( 0.041 to 0.1 ) | 0.07 ( 0.052 to 0.092 ) | 0.64 (0.2 to 1.08) |
| Belarus | 20 ( 17 to 25 ) | 0.164 ( 0.137 to 0.2 ) | 0.196 ( 0.162 to 0.238 ) |  | 32 ( 24 to 43 ) | 0.208 ( 0.157 to 0.28 ) | 0.339 ( 0.255 to 0.456 ) | 0.41 (0.19 to 0.63) |
| Belgium | 20 ( 16 to 24 ) | 0.138 ( 0.113 to 0.163 ) | 0.203 ( 0.165 to 0.241 ) |  | 27 ( 21 to 33 ) | 0.118 ( 0.095 to 0.144 ) | 0.233 ( 0.187 to 0.286 ) | -0.15 (-0.44 to 0.15) |
| Belize | 0 ( 0 to 0 ) | 0.026 ( 0.016 to 0.047 ) | 0.035 ( 0.018 to 0.068 ) |  | 0 ( 0 to 0 ) | 0.007 ( 0.006 to 0.009 ) | 0.005 ( 0.004 to 0.007 ) | -3.98 (-5.03 to -2.91) |
| Benin | 14 ( 7 to 29 ) | 0.243 ( 0.149 to 0.413 ) | 0.298 ( 0.147 to 0.607 ) |  | 23 ( 11 to 52 ) | 0.163 ( 0.096 to 0.292 ) | 0.17 ( 0.08 to 0.387 ) | -1.27 (-1.44 to -1.1) |
| Bermuda | 0 ( 0 to 0 ) | 0.014 ( 0.012 to 0.016 ) | 0.013 ( 0.011 to 0.016 ) |  | 0 ( 0 to 0 ) | 0.01 ( 0.008 to 0.013 ) | 0.023 ( 0.018 to 0.03 ) | -1.03 (-1.25 to -0.8) |
| Bhutan | 1 ( 0 to 2 ) | 0.105 ( 0.06 to 0.191 ) | 0.11 ( 0.046 to 0.248 ) |  | 1 ( 0 to 1 ) | 0.083 ( 0.044 to 0.154 ) | 0.067 ( 0.035 to 0.126 ) | -0.79 (-0.96 to -0.61) |
| Bolivia (Plurinational State of) | 18 ( 10 to 34 ) | 0.296 ( 0.199 to 0.475 ) | 0.275 ( 0.16 to 0.532 ) |  | 20 ( 13 to 31 ) | 0.208 ( 0.144 to 0.312 ) | 0.167 ( 0.11 to 0.266 ) | -1.16 (-1.24 to -1.08) |
| Bosnia and Herzegovina | 7 ( 5 to 9 ) | 0.182 ( 0.127 to 0.25 ) | 0.149 ( 0.106 to 0.206 ) |  | 9 ( 6 to 12 ) | 0.138 ( 0.094 to 0.189 ) | 0.259 ( 0.177 to 0.356 ) | -0.97 (-1.14 to -0.81) |
| Botswana | 2 ( 1 to 4 ) | 0.298 ( 0.176 to 0.62 ) | 0.162 ( 0.097 to 0.302 ) |  | 4 ( 2 to 8 ) | 0.246 ( 0.146 to 0.476 ) | 0.163 ( 0.093 to 0.321 ) | -0.55 (-0.66 to -0.44) |
| Brazil | 156 ( 140 to 174 ) | 0.148 ( 0.135 to 0.162 ) | 0.105 ( 0.094 to 0.117 ) |  | 230 ( 205 to 254 ) | 0.097 ( 0.086 to 0.108 ) | 0.104 ( 0.093 to 0.115 ) | -1.27 (-1.44 to -1.11) |
| Brunei Darussalam | 0 ( 0 to 1 ) | 0.199 ( 0.136 to 0.307 ) | 0.156 ( 0.09 to 0.28 ) |  | 1 ( 0 to 1 ) | 0.144 ( 0.103 to 0.209 ) | 0.112 ( 0.079 to 0.159 ) | -0.54 (-0.7 to -0.37) |
| Bulgaria | 20 ( 15 to 25 ) | 0.192 ( 0.147 to 0.25 ) | 0.225 ( 0.173 to 0.289 ) |  | 19 ( 14 to 27 ) | 0.142 ( 0.102 to 0.197 ) | 0.285 ( 0.204 to 0.394 ) | -0.95 (-1.22 to -0.67) |
| Burkina Faso | 29 ( 14 to 60 ) | 0.26 ( 0.147 to 0.447 ) | 0.3 ( 0.15 to 0.632 ) |  | 41 ( 21 to 84 ) | 0.171 ( 0.108 to 0.282 ) | 0.179 ( 0.092 to 0.368 ) | -1.26 (-1.57 to -0.95) |
| Burundi | 56 ( 37 to 81 ) | 1.513 ( 1.081 to 2.194 ) | 1.004 ( 0.674 to 1.45 ) |  | 84 ( 56 to 132 ) | 1.21 ( 0.796 to 1.931 ) | 0.632 ( 0.423 to 0.999 ) | -0.77 (-0.82 to -0.72) |
| Cabo Verde | 0 ( 0 to 1 ) | 0.139 ( 0.054 to 0.226 ) | 0.141 ( 0.064 to 0.256 ) |  | 1 ( 0 to 1 ) | 0.144 ( 0.055 to 0.235 ) | 0.107 ( 0.043 to 0.173 ) | 0.15 (-0.03 to 0.33) |
| Cambodia | 5 ( 2 to 9 ) | 0.064 ( 0.035 to 0.091 ) | 0.049 ( 0.022 to 0.084 ) |  | 8 ( 4 to 12 ) | 0.06 ( 0.033 to 0.088 ) | 0.045 ( 0.024 to 0.068 ) | -0.21 (-0.25 to -0.17) |
| Cameroon | 24 ( 13 to 49 ) | 0.222 ( 0.123 to 0.364 ) | 0.233 ( 0.12 to 0.469 ) |  | 39 ( 18 to 77 ) | 0.144 ( 0.085 to 0.232 ) | 0.122 ( 0.057 to 0.243 ) | -1.18 (-1.33 to -1.04) |
| Canada | 46 ( 40 to 53 ) | 0.144 ( 0.126 to 0.165 ) | 0.169 ( 0.147 to 0.194 ) |  | 68 ( 57 to 80 ) | 0.095 ( 0.08 to 0.111 ) | 0.181 ( 0.151 to 0.213 ) | -1.28 (-1.48 to -1.09) |
| Central African Republic | 6 ( 4 to 9 ) | 0.297 ( 0.205 to 0.502 ) | 0.206 ( 0.134 to 0.339 ) |  | 9 ( 6 to 15 ) | 0.272 ( 0.174 to 0.445 ) | 0.173 ( 0.103 to 0.281 ) | -0.26 (-0.33 to -0.18) |
| Chad | 16 ( 8 to 32 ) | 0.218 ( 0.135 to 0.37 ) | 0.265 ( 0.136 to 0.536 ) |  | 36 ( 17 to 74 ) | 0.174 ( 0.107 to 0.292 ) | 0.202 ( 0.097 to 0.419 ) | -0.75 (-0.82 to -0.68) |
| Chile | 12 ( 11 to 15 ) | 0.125 ( 0.106 to 0.148 ) | 0.093 ( 0.08 to 0.109 ) |  | 15 ( 12 to 18 ) | 0.06 ( 0.049 to 0.072 ) | 0.079 ( 0.064 to 0.096 ) | -2.15 (-2.32 to -1.97) |
| China | 654 ( 405 to 853 ) | 0.075 ( 0.047 to 0.095 ) | 0.056 ( 0.034 to 0.073 ) |  | 693 ( 369 to 912 ) | 0.042 ( 0.023 to 0.054 ) | 0.049 ( 0.026 to 0.064 ) | -1.68 (-1.84 to -1.52) |
| Colombia | 36 ( 31 to 42 ) | 0.165 ( 0.144 to 0.186 ) | 0.111 ( 0.096 to 0.13 ) |  | 48 ( 38 to 60 ) | 0.09 ( 0.073 to 0.112 ) | 0.097 ( 0.077 to 0.122 ) | -1.37 (-1.58 to -1.17) |
| Comoros | 4 ( 3 to 7 ) | 1.404 ( 1.027 to 2.093 ) | 0.921 ( 0.629 to 1.419 ) |  | 7 ( 5 to 12 ) | 1.257 ( 0.841 to 2.152 ) | 0.931 ( 0.613 to 1.58 ) | -0.55 (-0.65 to -0.45) |
| Congo | 4 ( 3 to 7 ) | 0.268 ( 0.184 to 0.466 ) | 0.174 ( 0.115 to 0.292 ) |  | 7 ( 4 to 10 ) | 0.208 ( 0.14 to 0.351 ) | 0.121 ( 0.079 to 0.187 ) | -0.88 (-0.97 to -0.79) |
| Cook Islands | 0 ( 0 to 0 ) | 0.031 ( 0.018 to 0.052 ) | 0.025 ( 0.014 to 0.05 ) |  | 0 ( 0 to 0 ) | 0.046 ( 0.02 to 0.115 ) | 0.04 ( 0.019 to 0.084 ) | -0.48 (-1.14 to 0.18) |
| Costa Rica | 4 ( 3 to 5 ) | 0.176 ( 0.15 to 0.206 ) | 0.127 ( 0.107 to 0.152 ) |  | 4 ( 3 to 5 ) | 0.079 ( 0.064 to 0.096 ) | 0.088 ( 0.071 to 0.108 ) | -2.22 (-2.62 to -1.82) |
| Coted'Ivoire | 11 ( 7 to 20 ) | 0.137 ( 0.098 to 0.196 ) | 0.089 ( 0.054 to 0.164 ) |  | 21 ( 12 to 35 ) | 0.122 ( 0.082 to 0.177 ) | 0.074 ( 0.042 to 0.125 ) | -0.19 (-0.3 to -0.08) |
| Croatia | 13 ( 11 to 16 ) | 0.233 ( 0.188 to 0.284 ) | 0.27 ( 0.22 to 0.33 ) |  | 13 ( 10 to 17 ) | 0.14 ( 0.104 to 0.187 ) | 0.307 ( 0.227 to 0.411 ) | -1.46 (-1.79 to -1.13) |
| Cuba | 19 ( 16 to 23 ) | 0.188 ( 0.157 to 0.225 ) | 0.174 ( 0.146 to 0.208 ) |  | 29 ( 23 to 35 ) | 0.144 ( 0.115 to 0.178 ) | 0.253 ( 0.202 to 0.314 ) | -0.56 (-0.73 to -0.39) |
| Cyprus | 1 ( 1 to 2 ) | 0.155 ( 0.116 to 0.234 ) | 0.142 ( 0.109 to 0.212 ) |  | 2 ( 1 to 3 ) | 0.105 ( 0.066 to 0.136 ) | 0.151 ( 0.097 to 0.2 ) | -0.93 (-1.11 to -0.75) |
| Czechia | 29 ( 22 to 37 ) | 0.216 ( 0.164 to 0.276 ) | 0.282 ( 0.214 to 0.361 ) |  | 27 ( 18 to 39 ) | 0.126 ( 0.086 to 0.177 ) | 0.256 ( 0.171 to 0.363 ) | -1.57 (-1.84 to -1.29) |
| Democratic People's Republic of Korea | 11 ( 7 to 16 ) | 0.06 ( 0.041 to 0.084 ) | 0.051 ( 0.034 to 0.077 ) |  | 17 ( 10 to 23 ) | 0.063 ( 0.039 to 0.093 ) | 0.063 ( 0.039 to 0.088 ) | 0.29 (0.17 to 0.4) |
| Democratic Republic of the Congo | 62 ( 40 to 95 ) | 0.241 ( 0.166 to 0.385 ) | 0.162 ( 0.106 to 0.248 ) |  | 108 ( 70 to 177 ) | 0.227 ( 0.142 to 0.424 ) | 0.12 ( 0.077 to 0.197 ) | -0.07 (-0.15 to 0.01) |
| Denmark | 15 ( 12 to 18 ) | 0.193 ( 0.161 to 0.23 ) | 0.288 ( 0.236 to 0.345 ) |  | 19 ( 15 to 23 ) | 0.163 ( 0.132 to 0.2 ) | 0.324 ( 0.258 to 0.4 ) | -0.65 (-0.76 to -0.53) |
| Djibouti | 3 ( 2 to 5 ) | 1.337 ( 0.899 to 2.04 ) | 0.775 ( 0.495 to 1.202 ) |  | 9 ( 6 to 15 ) | 1.2 ( 0.759 to 1.988 ) | 0.743 ( 0.454 to 1.221 ) | -0.36 (-0.53 to -0.19) |
| Dominica | 0 ( 0 to 0 ) | 0.169 ( 0.097 to 0.292 ) | 0.138 ( 0.078 to 0.238 ) |  | 0 ( 0 to 0 ) | 0.166 ( 0.086 to 0.271 ) | 0.197 ( 0.102 to 0.321 ) | -0.03 (-0.08 to 0.03) |
| Dominican Republic | 3 ( 2 to 5 ) | 0.064 ( 0.039 to 0.085 ) | 0.045 ( 0.025 to 0.065 ) |  | 5 ( 3 to 6 ) | 0.046 ( 0.03 to 0.061 ) | 0.042 ( 0.027 to 0.055 ) | -0.16 (-0.45 to 0.12) |
| Ecuador | 16 ( 12 to 20 ) | 0.226 ( 0.185 to 0.277 ) | 0.157 ( 0.121 to 0.202 ) |  | 27 ( 22 to 34 ) | 0.177 ( 0.138 to 0.224 ) | 0.151 ( 0.119 to 0.191 ) | -0.41 (-0.84 to 0.02) |
| Egypt | 14 ( 9 to 27 ) | 0.049 ( 0.034 to 0.088 ) | 0.025 ( 0.016 to 0.049 ) |  | 22 ( 14 to 41 ) | 0.038 ( 0.025 to 0.071 ) | 0.021 ( 0.013 to 0.039 ) | -0.53 (-0.81 to -0.25) |
| El Salvador | 4 ( 3 to 6 ) | 0.117 ( 0.077 to 0.165 ) | 0.081 ( 0.054 to 0.117 ) |  | 6 ( 4 to 9 ) | 0.095 ( 0.064 to 0.133 ) | 0.098 ( 0.065 to 0.137 ) | -0.74 (-0.86 to -0.62) |
| Equatorial Guinea | 1 ( 0 to 1 ) | 0.27 ( 0.179 to 0.448 ) | 0.197 ( 0.117 to 0.335 ) |  | 1 ( 1 to 2 ) | 0.163 ( 0.1 to 0.304 ) | 0.073 ( 0.042 to 0.127 ) | -1.92 (-2.18 to -1.66) |
| Eritrea | 32 ( 21 to 52 ) | 1.62 ( 1.185 to 2.463 ) | 0.948 ( 0.611 to 1.523 ) |  | 58 ( 36 to 95 ) | 1.485 ( 1.003 to 2.333 ) | 0.883 ( 0.551 to 1.443 ) | -0.34 (-0.4 to -0.28) |
| Estonia | 4 ( 3 to 5 ) | 0.193 ( 0.147 to 0.254 ) | 0.245 ( 0.187 to 0.326 ) |  | 5 ( 4 to 7 ) | 0.203 ( 0.146 to 0.274 ) | 0.415 ( 0.298 to 0.562 ) | -0.87 (-1.24 to -0.49) |
| Eswatini | 1 ( 1 to 2 ) | 0.319 ( 0.189 to 0.666 ) | 0.164 ( 0.095 to 0.3 ) |  | 2 ( 1 to 4 ) | 0.319 ( 0.171 to 0.671 ) | 0.187 ( 0.097 to 0.381 ) | 0.28 (0.03 to 0.54) |
| Ethiopia | 282 ( 181 to 415 ) | 0.865 ( 0.538 to 1.165 ) | 0.557 ( 0.359 to 0.82 ) |  | 347 ( 231 to 460 ) | 0.581 ( 0.366 to 0.776 ) | 0.319 ( 0.212 to 0.422 ) | -1.55 (-1.65 to -1.45) |
| Fiji | 0 ( 0 to 1 ) | 0.058 ( 0.025 to 0.137 ) | 0.038 ( 0.015 to 0.089 ) |  | 0 ( 0 to 1 ) | 0.053 ( 0.021 to 0.127 ) | 0.046 ( 0.018 to 0.107 ) | -0.24 (-0.53 to 0.06) |
| Finland | 11 ( 9 to 12 ) | 0.156 ( 0.132 to 0.178 ) | 0.216 ( 0.183 to 0.248 ) |  | 29 ( 23 to 35 ) | 0.239 ( 0.194 to 0.293 ) | 0.522 ( 0.42 to 0.641 ) | 1.28 (1.02 to 1.54) |
| France | 142 ( 126 to 157 ) | 0.18 ( 0.161 to 0.197 ) | 0.245 ( 0.218 to 0.271 ) |  | 190 ( 148 to 246 ) | 0.14 ( 0.112 to 0.176 ) | 0.287 ( 0.223 to 0.371 ) | -0.44 (-0.56 to -0.33) |
| Gabon | 2 ( 1 to 3 ) | 0.229 ( 0.157 to 0.41 ) | 0.16 ( 0.109 to 0.256 ) |  | 2 ( 1 to 4 ) | 0.19 ( 0.126 to 0.342 ) | 0.118 ( 0.075 to 0.193 ) | -0.56 (-0.61 to -0.52) |
| Gambia | 3 ( 2 to 5 ) | 0.374 ( 0.252 to 0.535 ) | 0.289 ( 0.168 to 0.51 ) |  | 6 ( 4 to 11 ) | 0.387 ( 0.256 to 0.616 ) | 0.263 ( 0.157 to 0.46 ) | -0.03 (-0.22 to 0.16) |
| Georgia | 5 ( 4 to 6 ) | 0.089 ( 0.071 to 0.104 ) | 0.095 ( 0.076 to 0.111 ) |  | 12 ( 10 to 15 ) | 0.218 ( 0.179 to 0.268 ) | 0.339 ( 0.282 to 0.402 ) | 3.61 (3.18 to 4.05) |
| Germany | 161 ( 134 to 190 ) | 0.134 ( 0.113 to 0.157 ) | 0.201 ( 0.168 to 0.238 ) |  | 290 ( 242 to 340 ) | 0.155 ( 0.131 to 0.18 ) | 0.339 ( 0.284 to 0.398 ) | 0.84 (0.67 to 1.01) |
| Ghana | 22 ( 9 to 52 ) | 0.095 ( 0.043 to 0.214 ) | 0.144 ( 0.058 to 0.344 ) |  | 26 ( 9 to 60 ) | 0.071 ( 0.025 to 0.152 ) | 0.076 ( 0.028 to 0.175 ) | -0.4 (-0.66 to -0.13) |
| Greece | 16 ( 14 to 18 ) | 0.111 ( 0.098 to 0.123 ) | 0.156 ( 0.137 to 0.173 ) |  | 26 ( 22 to 30 ) | 0.108 ( 0.095 to 0.123 ) | 0.256 ( 0.221 to 0.293 ) | 0.22 (0.04 to 0.4) |
| Greenland | 0 ( 0 to 0 ) | 0.064 ( 0.028 to 0.09 ) | 0.043 ( 0.017 to 0.062 ) |  | 0 ( 0 to 0 ) | 0.026 ( 0.016 to 0.037 ) | 0.03 ( 0.018 to 0.042 ) | -2.46 (-2.79 to -2.13) |
| Grenada | 0 ( 0 to 0 ) | 0.074 ( 0.058 to 0.091 ) | 0.066 ( 0.051 to 0.083 ) |  | 0 ( 0 to 0 ) | 0.058 ( 0.047 to 0.07 ) | 0.062 ( 0.049 to 0.076 ) | -0.45 (-0.82 to -0.08) |
| Guam | 0 ( 0 to 0 ) | 0.017 ( 0.009 to 0.029 ) | 0.012 ( 0.006 to 0.026 ) |  | 0 ( 0 to 0 ) | 0.013 ( 0.006 to 0.026 ) | 0.014 ( 0.007 to 0.025 ) | 0.49 (0.12 to 0.87) |
| Guatemala | 12 ( 8 to 19 ) | 0.229 ( 0.193 to 0.278 ) | 0.139 ( 0.092 to 0.221 ) |  | 15 ( 12 to 18 ) | 0.135 ( 0.113 to 0.159 ) | 0.095 ( 0.077 to 0.116 ) | -1.54 (-1.65 to -1.43) |
| Guinea | 28 ( 15 to 55 ) | 0.32 ( 0.187 to 0.565 ) | 0.468 ( 0.242 to 0.924 ) |  | 36 ( 17 to 76 ) | 0.233 ( 0.128 to 0.419 ) | 0.267 ( 0.125 to 0.564 ) | -0.72 (-0.87 to -0.57) |
| Guinea-Bissau | 3 ( 2 to 7 ) | 0.296 ( 0.173 to 0.521 ) | 0.326 ( 0.16 to 0.709 ) |  | 3 ( 2 to 7 ) | 0.181 ( 0.115 to 0.296 ) | 0.154 ( 0.076 to 0.325 ) | -1.25 (-1.43 to -1.06) |
| Guyana | 0 ( 0 to 0 ) | 0.02 ( 0.016 to 0.025 ) | 0.013 ( 0.01 to 0.017 ) |  | 1 ( 0 to 1 ) | 0.106 ( 0.078 to 0.145 ) | 0.088 ( 0.064 to 0.12 ) | 3.86 (1.95 to 5.81) |
| Haiti | 3 ( 2 to 4 ) | 0.072 ( 0.046 to 0.105 ) | 0.044 ( 0.025 to 0.064 ) |  | 5 ( 3 to 7 ) | 0.063 ( 0.034 to 0.089 ) | 0.037 ( 0.02 to 0.054 ) | -0.32 (-0.36 to -0.28) |
| Honduras | 6 ( 4 to 10 ) | 0.173 ( 0.113 to 0.239 ) | 0.121 ( 0.077 to 0.205 ) |  | 10 ( 7 to 15 ) | 0.167 ( 0.115 to 0.228 ) | 0.104 ( 0.072 to 0.147 ) | -0.09 (-0.17 to -0.01) |
| Hungary | 36 ( 30 to 43 ) | 0.261 ( 0.218 to 0.315 ) | 0.342 ( 0.286 to 0.409 ) |  | 30 ( 23 to 40 ) | 0.158 ( 0.117 to 0.21 ) | 0.317 ( 0.236 to 0.422 ) | -1.43 (-1.62 to -1.23) |
| Iceland | 0 ( 0 to 0 ) | 0.134 ( 0.115 to 0.154 ) | 0.149 ( 0.128 to 0.172 ) |  | 1 ( 1 to 1 ) | 0.132 ( 0.108 to 0.16 ) | 0.213 ( 0.174 to 0.261 ) | 0.15 (-0.14 to 0.45) |
| India | 836 ( 441 to 1194 ) | 0.091 ( 0.052 to 0.122 ) | 0.098 ( 0.052 to 0.14 ) |  | 763 ( 500 to 987 ) | 0.066 ( 0.043 to 0.085 ) | 0.054 ( 0.035 to 0.07 ) | -1.15 (-1.29 to -1) |
| Indonesia | 59 ( 30 to 79 ) | 0.045 ( 0.026 to 0.057 ) | 0.032 ( 0.016 to 0.043 ) |  | 101 ( 55 to 134 ) | 0.046 ( 0.025 to 0.059 ) | 0.036 ( 0.02 to 0.048 ) | 0.12 (-0.01 to 0.25) |
| Iran (Islamic Republic of) | 1 ( 0 to 2 ) | 0.002 ( 0.001 to 0.004 ) | 0.001 ( 0 to 0.003 ) |  | 2 ( 1 to 3 ) | 0.003 ( 0.001 to 0.004 ) | 0.003 ( 0.001 to 0.004 ) | 1.85 (1.38 to 2.31) |
| Iraq | 9 ( 6 to 14 ) | 0.063 ( 0.046 to 0.088 ) | 0.05 ( 0.034 to 0.078 ) |  | 17 ( 11 to 25 ) | 0.059 ( 0.041 to 0.081 ) | 0.042 ( 0.028 to 0.06 ) | -0.41 (-0.49 to -0.33) |
| Ireland | 9 ( 8 to 10 ) | 0.221 ( 0.192 to 0.25 ) | 0.247 ( 0.214 to 0.28 ) |  | 9 ( 7 to 12 ) | 0.121 ( 0.096 to 0.15 ) | 0.192 ( 0.151 to 0.239 ) | -1.38 (-1.6 to -1.16) |
| Israel | 4 ( 3 to 4 ) | 0.076 ( 0.061 to 0.093 ) | 0.073 ( 0.059 to 0.09 ) |  | 7 ( 6 to 9 ) | 0.06 ( 0.049 to 0.073 ) | 0.077 ( 0.063 to 0.095 ) | -0.61 (-0.7 to -0.52) |
| Italy | 116 ( 105 to 126 ) | 0.138 ( 0.125 to 0.15 ) | 0.204 ( 0.184 to 0.223 ) |  | 193 ( 162 to 225 ) | 0.134 ( 0.116 to 0.154 ) | 0.322 ( 0.271 to 0.376 ) | -0.04 (-0.18 to 0.09) |
| Jamaica | 2 ( 1 to 2 ) | 0.077 ( 0.055 to 0.108 ) | 0.069 ( 0.048 to 0.103 ) |  | 2 ( 1 to 2 ) | 0.059 ( 0.042 to 0.079 ) | 0.065 ( 0.047 to 0.087 ) | -0.72 (-1.15 to -0.3) |
| Japan | 36 ( 34 to 38 ) | 0.025 ( 0.024 to 0.026 ) | 0.029 ( 0.027 to 0.03 ) |  | 81 ( 69 to 91 ) | 0.025 ( 0.022 to 0.027 ) | 0.063 ( 0.054 to 0.071 ) | -0.05 (-0.34 to 0.24) |
| Jordan | 1 ( 1 to 2 ) | 0.067 ( 0.049 to 0.092 ) | 0.04 ( 0.028 to 0.057 ) |  | 4 ( 2 to 5 ) | 0.049 ( 0.029 to 0.069 ) | 0.03 ( 0.019 to 0.042 ) | -1.17 (-1.34 to -1) |
| Kazakhstan | 20 ( 13 to 31 ) | 0.144 ( 0.096 to 0.212 ) | 0.121 ( 0.079 to 0.187 ) |  | 15 ( 10 to 22 ) | 0.089 ( 0.055 to 0.13 ) | 0.08 ( 0.05 to 0.118 ) | -2.04 (-2.51 to -1.58) |
| Kenya | 312 ( 203 to 421 ) | 1.539 ( 1.094 to 1.982 ) | 1.347 ( 0.876 to 1.818 ) |  | 507 ( 379 to 655 ) | 1.564 ( 1.111 to 2.013 ) | 1.014 ( 0.758 to 1.309 ) | 0.48 (0.31 to 0.65) |
| Kiribati | 0 ( 0 to 0 ) | 0.081 ( 0.048 to 0.157 ) | 0.059 ( 0.03 to 0.111 ) |  | 0 ( 0 to 0 ) | 0.078 ( 0.043 to 0.158 ) | 0.055 ( 0.028 to 0.103 ) | -0.34 (-0.47 to -0.22) |
| Kuwait | 1 ( 0 to 1 ) | 0.053 ( 0.044 to 0.061 ) | 0.03 ( 0.024 to 0.038 ) |  | 1 ( 1 to 1 ) | 0.03 ( 0.023 to 0.037 ) | 0.018 ( 0.014 to 0.022 ) | -0.44 (-2.23 to 1.38) |
| Kyrgyzstan | 6 ( 5 to 9 ) | 0.182 ( 0.134 to 0.249 ) | 0.145 ( 0.102 to 0.211 ) |  | 7 ( 5 to 11 ) | 0.138 ( 0.095 to 0.195 ) | 0.108 ( 0.075 to 0.154 ) | -0.72 (-1.97 to 0.55) |
| Lao People's Democratic Republic | 2 ( 1 to 4 ) | 0.063 ( 0.032 to 0.093 ) | 0.051 ( 0.022 to 0.093 ) |  | 3 ( 1 to 5 ) | 0.05 ( 0.026 to 0.075 ) | 0.037 ( 0.018 to 0.061 ) | -0.72 (-0.78 to -0.65) |
| Latvia | 5 ( 4 to 6 ) | 0.145 ( 0.108 to 0.186 ) | 0.188 ( 0.141 to 0.242 ) |  | 7 ( 5 to 9 ) | 0.183 ( 0.135 to 0.243 ) | 0.375 ( 0.274 to 0.504 ) | 0.31 (0.04 to 0.57) |
| Lebanon | 2 ( 2 to 3 ) | 0.101 ( 0.075 to 0.166 ) | 0.074 ( 0.055 to 0.115 ) |  | 5 ( 3 to 8 ) | 0.081 ( 0.055 to 0.124 ) | 0.089 ( 0.061 to 0.141 ) | -0.49 (-0.58 to -0.39) |
| Lesotho | 3 ( 2 to 6 ) | 0.295 ( 0.171 to 0.627 ) | 0.197 ( 0.114 to 0.382 ) |  | 4 ( 2 to 9 ) | 0.354 ( 0.197 to 0.761 ) | 0.227 ( 0.123 to 0.478 ) | 0.98 (0.79 to 1.18) |
| Liberia | 6 ( 3 to 12 ) | 0.204 ( 0.11 to 0.349 ) | 0.226 ( 0.109 to 0.479 ) |  | 5 ( 3 to 12 ) | 0.131 ( 0.077 to 0.217 ) | 0.099 ( 0.049 to 0.214 ) | -1.51 (-1.9 to -1.11) |
| Libya | 2 ( 1 to 4 ) | 0.056 ( 0.036 to 0.085 ) | 0.051 ( 0.03 to 0.094 ) |  | 2 ( 1 to 3 ) | 0.046 ( 0.029 to 0.066 ) | 0.034 ( 0.021 to 0.05 ) | -0.59 (-0.65 to -0.53) |
| Lithuania | 8 ( 6 to 10 ) | 0.186 ( 0.144 to 0.236 ) | 0.223 ( 0.173 to 0.284 ) |  | 13 ( 10 to 17 ) | 0.239 ( 0.184 to 0.311 ) | 0.489 ( 0.373 to 0.64 ) | 0.4 (0.17 to 0.64) |
| Luxembourg | 1 ( 1 to 1 ) | 0.149 ( 0.136 to 0.164 ) | 0.207 ( 0.189 to 0.229 ) |  | 1 ( 1 to 1 ) | 0.099 ( 0.082 to 0.116 ) | 0.165 ( 0.138 to 0.193 ) | -0.8 (-1.01 to -0.59) |
| Madagascar | 101 ( 70 to 151 ) | 1.269 ( 0.954 to 1.865 ) | 0.85 ( 0.59 to 1.271 ) |  | 169 ( 108 to 270 ) | 1.005 ( 0.677 to 1.59 ) | 0.591 ( 0.378 to 0.944 ) | -0.77 (-0.84 to -0.71) |
| Malawi | 225 ( 151 to 324 ) | 3.582 ( 2.448 to 4.79 ) | 2.292 ( 1.541 to 3.301 ) |  | 371 ( 221 to 555 ) | 3.6 ( 2.212 to 4.98 ) | 1.906 ( 1.137 to 2.855 ) | -0.02 (-0.16 to 0.11) |
| Malaysia | 9 ( 4 to 17 ) | 0.063 ( 0.032 to 0.101 ) | 0.053 ( 0.025 to 0.097 ) |  | 12 ( 7 to 18 ) | 0.046 ( 0.024 to 0.066 ) | 0.039 ( 0.021 to 0.056 ) | -0.96 (-1.16 to -0.77) |
| Maldives | 0 ( 0 to 0 ) | 0.024 ( 0.016 to 0.035 ) | 0.014 ( 0.009 to 0.021 ) |  | 0 ( 0 to 0 ) | 0.014 ( 0.009 to 0.019 ) | 0.009 ( 0.006 to 0.013 ) | -1.96 (-2.03 to -1.88) |
| Mali | 68 ( 42 to 111 ) | 0.963 ( 0.678 to 1.354 ) | 0.782 ( 0.481 to 1.282 ) |  | 109 ( 62 to 214 ) | 0.764 ( 0.487 to 1.21 ) | 0.451 ( 0.256 to 0.889 ) | -0.65 (-0.75 to -0.56) |
| Malta | 1 ( 0 to 1 ) | 0.123 ( 0.107 to 0.139 ) | 0.139 ( 0.122 to 0.158 ) |  | 1 ( 1 to 1 ) | 0.101 ( 0.079 to 0.125 ) | 0.216 ( 0.169 to 0.267 ) | -0.13 (-0.32 to 0.06) |
| Marshall Islands | 0 ( 0 to 0 ) | 0.037 ( 0.025 to 0.06 ) | 0.027 ( 0.014 to 0.061 ) |  | 0 ( 0 to 0 ) | 0.038 ( 0.021 to 0.067 ) | 0.03 ( 0.015 to 0.059 ) | 0.02 (-0.14 to 0.18) |
| Mauritania | 4 ( 2 to 9 ) | 0.205 ( 0.121 to 0.348 ) | 0.216 ( 0.115 to 0.458 ) |  | 5 ( 3 to 9 ) | 0.124 ( 0.081 to 0.192 ) | 0.105 ( 0.058 to 0.198 ) | -1.83 (-1.97 to -1.69) |
| Mauritius | 0 ( 0 to 0 ) | 0.021 ( 0.019 to 0.023 ) | 0.014 ( 0.013 to 0.016 ) |  | 1 ( 1 to 1 ) | 0.042 ( 0.037 to 0.046 ) | 0.054 ( 0.048 to 0.06 ) | 3.21 (2.21 to 4.21) |
| Mexico | 98 ( 89 to 109 ) | 0.167 ( 0.155 to 0.181 ) | 0.115 ( 0.104 to 0.128 ) |  | 110 ( 97 to 125 ) | 0.097 ( 0.085 to 0.11 ) | 0.085 ( 0.075 to 0.097 ) | -1.68 (-1.77 to -1.58) |
| Micronesia (Federated States of) | 0 ( 0 to 0 ) | 0.043 ( 0.027 to 0.074 ) | 0.036 ( 0.018 to 0.079 ) |  | 0 ( 0 to 0 ) | 0.037 ( 0.023 to 0.063 ) | 0.028 ( 0.017 to 0.053 ) | -0.24 (-0.36 to -0.12) |
| Monaco | 0 ( 0 to 0 ) | 0.008 ( 0.005 to 0.012 ) | 0.018 ( 0.01 to 0.026 ) |  | 0 ( 0 to 0 ) | 0.008 ( 0.005 to 0.011 ) | 0.019 ( 0.012 to 0.026 ) | -0.29 (-0.33 to -0.24) |
| Mongolia | 2 ( 1 to 3 ) | 0.126 ( 0.086 to 0.187 ) | 0.087 ( 0.051 to 0.152 ) |  | 3 ( 2 to 4 ) | 0.106 ( 0.068 to 0.15 ) | 0.084 ( 0.053 to 0.134 ) | -0.65 (-0.76 to -0.54) |
| Montenegro | 0 ( 0 to 0 ) | 0.06 ( 0.041 to 0.082 ) | 0.058 ( 0.039 to 0.078 ) |  | 1 ( 0 to 1 ) | 0.064 ( 0.043 to 0.087 ) | 0.092 ( 0.061 to 0.125 ) | 0.1 (-0.12 to 0.32) |
| Morocco | 45 ( 25 to 80 ) | 0.166 ( 0.104 to 0.267 ) | 0.176 ( 0.098 to 0.315 ) |  | 40 ( 27 to 60 ) | 0.121 ( 0.082 to 0.182 ) | 0.108 ( 0.074 to 0.162 ) | -0.77 (-0.88 to -0.67) |
| Mozambique | 160 ( 108 to 243 ) | 1.629 ( 1.206 to 2.275 ) | 1.196 ( 0.812 to 1.821 ) |  | 270 ( 176 to 424 ) | 1.539 ( 1.051 to 2.529 ) | 0.87 ( 0.565 to 1.366 ) | 0.06 (-0.04 to 0.17) |
| Myanmar | 20 ( 9 to 37 ) | 0.064 ( 0.034 to 0.105 ) | 0.05 ( 0.023 to 0.092 ) |  | 25 ( 13 to 40 ) | 0.052 ( 0.027 to 0.08 ) | 0.044 ( 0.023 to 0.071 ) | -0.87 (-0.98 to -0.76) |
| Namibia | 11 ( 8 to 14 ) | 1.373 ( 0.961 to 1.813 ) | 0.757 ( 0.535 to 1.023 ) |  | 23 ( 14 to 33 ) | 1.49 ( 0.907 to 2.076 ) | 0.949 ( 0.563 to 1.347 ) | 0.27 (0.11 to 0.42) |
| Nauru | 0 ( 0 to 0 ) | 0.043 ( 0.025 to 0.077 ) | 0.038 ( 0.019 to 0.087 ) |  | 0 ( 0 to 0 ) | 0.044 ( 0.026 to 0.079 ) | 0.036 ( 0.017 to 0.079 ) | 0.02 (-0.22 to 0.26) |
| Nepal | 26 ( 14 to 53 ) | 0.111 ( 0.065 to 0.208 ) | 0.133 ( 0.073 to 0.271 ) |  | 21 ( 12 to 36 ) | 0.077 ( 0.046 to 0.138 ) | 0.067 ( 0.04 to 0.117 ) | -0.95 (-1.12 to -0.77) |
| Netherlands | 15 ( 13 to 17 ) | 0.077 ( 0.068 to 0.087 ) | 0.099 ( 0.087 to 0.113 ) |  | 17 ( 13 to 22 ) | 0.05 ( 0.039 to 0.062 ) | 0.099 ( 0.077 to 0.125 ) | -0.92 (-1.04 to -0.8) |
| New Zealand | 10 ( 8 to 11 ) | 0.243 ( 0.205 to 0.285 ) | 0.281 ( 0.238 to 0.329 ) |  | 16 ( 13 to 20 ) | 0.194 ( 0.16 to 0.235 ) | 0.314 ( 0.258 to 0.383 ) | -0.52 (-0.67 to -0.37) |
| Nicaragua | 6 ( 3 to 14 ) | 0.174 ( 0.112 to 0.299 ) | 0.158 ( 0.085 to 0.358 ) |  | 5 ( 4 to 7 ) | 0.107 ( 0.077 to 0.14 ) | 0.079 ( 0.058 to 0.108 ) | -1.29 (-1.48 to -1.1) |
| Niger | 27 ( 13 to 65 ) | 0.268 ( 0.155 to 0.521 ) | 0.337 ( 0.159 to 0.805 ) |  | 45 ( 21 to 104 ) | 0.163 ( 0.099 to 0.291 ) | 0.179 ( 0.084 to 0.415 ) | -1.62 (-1.83 to -1.41) |
| Nigeria | 300 ( 168 to 401 ) | 0.451 ( 0.269 to 0.568 ) | 0.333 ( 0.187 to 0.445 ) |  | 563 ( 273 to 822 ) | 0.392 ( 0.219 to 0.529 ) | 0.244 ( 0.118 to 0.356 ) | -0.49 (-0.53 to -0.44) |
| Niue | 0 ( 0 to 0 ) | 0.044 ( 0.026 to 0.087 ) | 0.046 ( 0.026 to 0.094 ) |  | 0 ( 0 to 0 ) | 0.084 ( 0.035 to 0.232 ) | 0.071 ( 0.033 to 0.178 ) | 0.22 (-0.46 to 0.9) |
| North Macedonia | 3 ( 2 to 4 ) | 0.175 ( 0.114 to 0.232 ) | 0.154 ( 0.102 to 0.205 ) |  | 4 ( 3 to 6 ) | 0.156 ( 0.099 to 0.205 ) | 0.203 ( 0.133 to 0.279 ) | -0.58 (-0.82 to -0.34) |
| Northern Mariana Islands | 0 ( 0 to 0 ) | 0.026 ( 0.014 to 0.037 ) | 0.015 ( 0.008 to 0.024 ) |  | 0 ( 0 to 0 ) | 0.027 ( 0.014 to 0.036 ) | 0.026 ( 0.013 to 0.035 ) | 0.56 (-0.07 to 1.19) |
| Norway | 14 ( 13 to 15 ) | 0.206 ( 0.189 to 0.225 ) | 0.323 ( 0.295 to 0.354 ) |  | 5 ( 4 to 5 ) | 0.045 ( 0.038 to 0.054 ) | 0.084 ( 0.069 to 0.101 ) | -5.33 (-5.74 to -4.93) |
| Oman | 1 ( 0 to 1 ) | 0.068 ( 0.028 to 0.105 ) | 0.036 ( 0.016 to 0.06 ) |  | 1 ( 0 to 1 ) | 0.044 ( 0.017 to 0.066 ) | 0.021 ( 0.008 to 0.032 ) | -1.01 (-1.24 to -0.79) |
| Pakistan | 183 ( 118 to 271 ) | 0.208 ( 0.135 to 0.312 ) | 0.165 ( 0.106 to 0.244 ) |  | 414 ( 258 to 594 ) | 0.23 ( 0.15 to 0.316 ) | 0.176 ( 0.109 to 0.252 ) | 0.36 (0.23 to 0.48) |
| Palau | 0 ( 0 to 0 ) | 0.019 ( 0.009 to 0.04 ) | 0.016 ( 0.008 to 0.036 ) |  | 0 ( 0 to 0 ) | 0.016 ( 0.008 to 0.041 ) | 0.013 ( 0.007 to 0.027 ) | -0.27 (-0.38 to -0.16) |
| Palestine | 0 ( 0 to 1 ) | 0.039 ( 0.022 to 0.052 ) | 0.02 ( 0.011 to 0.03 ) |  | 1 ( 0 to 1 ) | 0.032 ( 0.017 to 0.042 ) | 0.015 ( 0.008 to 0.02 ) | -0.53 (-0.68 to -0.39) |
| Panama | 2 ( 2 to 3 ) | 0.111 ( 0.09 to 0.148 ) | 0.087 ( 0.065 to 0.132 ) |  | 3 ( 3 to 4 ) | 0.078 ( 0.061 to 0.099 ) | 0.08 ( 0.063 to 0.1 ) | -0.93 (-1.04 to -0.82) |
| Papua New Guinea | 1 ( 1 to 3 ) | 0.036 ( 0.021 to 0.07 ) | 0.032 ( 0.014 to 0.083 ) |  | 4 ( 2 to 10 ) | 0.038 ( 0.021 to 0.081 ) | 0.035 ( 0.016 to 0.096 ) | 0.17 (0.02 to 0.33) |
| Paraguay | 5 ( 3 to 7 ) | 0.147 ( 0.094 to 0.209 ) | 0.121 ( 0.071 to 0.182 ) |  | 8 ( 5 to 12 ) | 0.141 ( 0.092 to 0.209 ) | 0.115 ( 0.075 to 0.172 ) | 0.2 (0.08 to 0.33) |
| Peru | 40 ( 25 to 65 ) | 0.201 ( 0.134 to 0.292 ) | 0.186 ( 0.118 to 0.302 ) |  | 36 ( 25 to 50 ) | 0.106 ( 0.074 to 0.15 ) | 0.098 ( 0.068 to 0.137 ) | -1.87 (-1.99 to -1.75) |
| Philippines | 46 ( 22 to 69 ) | 0.097 ( 0.052 to 0.127 ) | 0.073 ( 0.035 to 0.109 ) |  | 87 ( 45 to 113 ) | 0.095 ( 0.051 to 0.122 ) | 0.077 ( 0.04 to 0.1 ) | 0.25 (0.13 to 0.36) |
| Poland | 132 ( 121 to 143 ) | 0.324 ( 0.294 to 0.352 ) | 0.347 ( 0.317 to 0.376 ) |  | 130 ( 114 to 148 ) | 0.18 ( 0.158 to 0.203 ) | 0.341 ( 0.299 to 0.386 ) | -2.17 (-2.53 to -1.8) |
| Portugal | 21 ( 18 to 24 ) | 0.175 ( 0.151 to 0.199 ) | 0.21 ( 0.182 to 0.241 ) |  | 26 ( 21 to 33 ) | 0.115 ( 0.096 to 0.141 ) | 0.246 ( 0.2 to 0.309 ) | -0.98 (-1.32 to -0.64) |
| Puerto Rico | 2 ( 1 to 2 ) | 0.047 ( 0.039 to 0.057 ) | 0.045 ( 0.037 to 0.053 ) |  | 3 ( 2 to 3 ) | 0.037 ( 0.029 to 0.046 ) | 0.083 ( 0.064 to 0.103 ) | -0.57 (-1.11 to -0.03) |
| Qatar | 0 ( 0 to 0 ) | 0.068 ( 0.042 to 0.105 ) | 0.016 ( 0.01 to 0.026 ) |  | 0 ( 0 to 1 ) | 0.054 ( 0.025 to 0.084 ) | 0.013 ( 0.007 to 0.025 ) | -0.33 (-0.77 to 0.11) |
| Republic of Korea | 17 ( 10 to 21 ) | 0.052 ( 0.032 to 0.066 ) | 0.037 ( 0.023 to 0.048 ) |  | 19 ( 10 to 25 ) | 0.024 ( 0.015 to 0.032 ) | 0.036 ( 0.019 to 0.048 ) | -2.5 (-2.74 to -2.27) |
| Republic of Moldova | 7 ( 5 to 9 ) | 0.16 ( 0.124 to 0.216 ) | 0.156 ( 0.121 to 0.211 ) |  | 6 ( 5 to 8 ) | 0.112 ( 0.093 to 0.134 ) | 0.177 ( 0.148 to 0.21 ) | -1.2 (-1.38 to -1.02) |
| Romania | 53 ( 40 to 69 ) | 0.22 ( 0.167 to 0.292 ) | 0.227 ( 0.172 to 0.294 ) |  | 58 ( 45 to 72 ) | 0.161 ( 0.125 to 0.201 ) | 0.304 ( 0.237 to 0.378 ) | -1.29 (-1.43 to -1.15) |
| Russian Federation | 243 ( 196 to 292 ) | 0.143 ( 0.116 to 0.172 ) | 0.161 ( 0.13 to 0.193 ) |  | 303 ( 267 to 340 ) | 0.131 ( 0.116 to 0.147 ) | 0.209 ( 0.184 to 0.235 ) | -0.63 (-0.92 to -0.35) |
| Rwanda | 80 ( 55 to 124 ) | 1.696 ( 1.275 to 2.477 ) | 1.108 ( 0.763 to 1.73 ) |  | 95 ( 62 to 162 ) | 1.194 ( 0.779 to 2.085 ) | 0.717 ( 0.466 to 1.218 ) | -1.57 (-1.73 to -1.42) |
| Saint Kitts and Nevis | 0 ( 0 to 0 ) | 0.018 ( 0.015 to 0.02 ) | 0.015 ( 0.013 to 0.017 ) |  | 0 ( 0 to 0 ) | 0.01 ( 0.008 to 0.012 ) | 0.009 ( 0.008 to 0.011 ) | -1.61 (-1.81 to -1.41) |
| Saint Lucia | 0 ( 0 to 0 ) | 0.02 ( 0.017 to 0.023 ) | 0.012 ( 0.01 to 0.015 ) |  | 0 ( 0 to 0 ) | 0.014 ( 0.011 to 0.017 ) | 0.017 ( 0.014 to 0.021 ) | -1.34 (-1.48 to -1.19) |
| Saint Vincent and the Grenadines | 0 ( 0 to 1 ) | 0.362 ( 0.292 to 0.508 ) | 0.294 ( 0.217 to 0.466 ) |  | 0 ( 0 to 0 ) | 0.114 ( 0.095 to 0.134 ) | 0.124 ( 0.104 to 0.146 ) | -3.29 (-3.72 to -2.85) |
| Samoa | 0 ( 0 to 1 ) | 0.232 ( 0.135 to 0.558 ) | 0.14 ( 0.083 to 0.296 ) |  | 0 ( 0 to 1 ) | 0.213 ( 0.123 to 0.481 ) | 0.156 ( 0.091 to 0.331 ) | -0.19 (-0.24 to -0.14) |
| San Marino | 0 ( 0 to 0 ) | 0.103 ( 0.071 to 0.145 ) | 0.151 ( 0.104 to 0.214 ) |  | 0 ( 0 to 0 ) | 0.056 ( 0.033 to 0.089 ) | 0.129 ( 0.075 to 0.198 ) | -1.03 (-1.36 to -0.7) |
| Sao Tome and Principe | 0 ( 0 to 1 ) | 0.204 ( 0.12 to 0.372 ) | 0.228 ( 0.117 to 0.493 ) |  | 0 ( 0 to 0 ) | 0.132 ( 0.085 to 0.21 ) | 0.099 ( 0.054 to 0.185 ) | -1.27 (-1.75 to -0.8) |
| Saudi Arabia | 7 ( 2 to 12 ) | 0.062 ( 0.024 to 0.096 ) | 0.043 ( 0.011 to 0.078 ) |  | 10 ( 4 to 15 ) | 0.044 ( 0.017 to 0.067 ) | 0.026 ( 0.01 to 0.041 ) | -0.8 (-1.23 to -0.36) |
| Senegal | 22 ( 11 to 47 ) | 0.243 ( 0.136 to 0.424 ) | 0.286 ( 0.138 to 0.618 ) |  | 24 ( 12 to 47 ) | 0.165 ( 0.104 to 0.264 ) | 0.149 ( 0.079 to 0.293 ) | -0.97 (-1.25 to -0.68) |
| Serbia | 20 ( 13 to 27 ) | 0.213 ( 0.133 to 0.283 ) | 0.211 ( 0.131 to 0.281 ) |  | 25 ( 15 to 33 ) | 0.151 ( 0.09 to 0.2 ) | 0.28 ( 0.165 to 0.369 ) | -1.39 (-1.53 to -1.24) |
| Seychelles | 0 ( 0 to 0 ) | 0.075 ( 0.042 to 0.098 ) | 0.059 ( 0.033 to 0.077 ) |  | 0 ( 0 to 0 ) | 0.053 ( 0.031 to 0.068 ) | 0.056 ( 0.031 to 0.072 ) | -0.8 (-0.93 to -0.67) |
| Sierra Leone | 10 ( 5 to 19 ) | 0.211 ( 0.12 to 0.35 ) | 0.241 ( 0.118 to 0.468 ) |  | 12 ( 6 to 24 ) | 0.146 ( 0.086 to 0.248 ) | 0.133 ( 0.064 to 0.276 ) | -1.05 (-1.22 to -0.89) |
| Singapore | 1 ( 1 to 1 ) | 0.053 ( 0.045 to 0.064 ) | 0.038 ( 0.033 to 0.045 ) |  | 2 ( 1 to 2 ) | 0.025 ( 0.02 to 0.03 ) | 0.03 ( 0.024 to 0.037 ) | -2.52 (-3.04 to -2) |
| Slovakia | 13 ( 9 to 17 ) | 0.218 ( 0.149 to 0.29 ) | 0.242 ( 0.162 to 0.322 ) |  | 15 ( 9 to 21 ) | 0.159 ( 0.103 to 0.22 ) | 0.27 ( 0.175 to 0.378 ) | -1.02 (-1.05 to -0.99) |
| Slovenia | 3 ( 3 to 4 ) | 0.132 ( 0.103 to 0.164 ) | 0.163 ( 0.127 to 0.202 ) |  | 3 ( 2 to 5 ) | 0.073 ( 0.052 to 0.096 ) | 0.165 ( 0.118 to 0.222 ) | -1.8 (-1.89 to -1.71) |
| Solomon Islands | 0 ( 0 to 0 ) | 0.037 ( 0.02 to 0.067 ) | 0.032 ( 0.012 to 0.083 ) |  | 0 ( 0 to 1 ) | 0.041 ( 0.024 to 0.08 ) | 0.036 ( 0.017 to 0.087 ) | 0.44 (0.18 to 0.7) |
| Somalia | 74 ( 47 to 125 ) | 1.71 ( 1.219 to 2.644 ) | 0.937 ( 0.591 to 1.572 ) |  | 155 ( 85 to 269 ) | 1.537 ( 0.943 to 2.48 ) | 0.717 ( 0.392 to 1.243 ) | -0.3 (-0.39 to -0.21) |
| South Africa | 28 ( 19 to 38 ) | 0.106 ( 0.071 to 0.143 ) | 0.076 ( 0.051 to 0.102 ) |  | 58 ( 35 to 77 ) | 0.126 ( 0.075 to 0.163 ) | 0.103 ( 0.062 to 0.136 ) | 0.73 (0.61 to 0.85) |
| South Sudan | 50 ( 32 to 79 ) | 1.31 ( 0.904 to 2.101 ) | 0.847 ( 0.546 to 1.337 ) |  | 66 ( 41 to 102 ) | 1.241 ( 0.792 to 2.057 ) | 0.678 ( 0.426 to 1.055 ) | -0.24 (-0.38 to -0.1) |
| Spain | 62 ( 55 to 70 ) | 0.121 ( 0.108 to 0.135 ) | 0.16 ( 0.143 to 0.18 ) |  | 91 ( 74 to 110 ) | 0.094 ( 0.077 to 0.113 ) | 0.2 ( 0.162 to 0.242 ) | -0.37 (-0.57 to -0.16) |
| Sri Lanka | 9 ( 7 to 12 ) | 0.074 ( 0.053 to 0.098 ) | 0.052 ( 0.038 to 0.07 ) |  | 12 ( 7 to 18 ) | 0.049 ( 0.029 to 0.071 ) | 0.055 ( 0.032 to 0.08 ) | -1.06 (-1.26 to -0.86) |
| Sudan | 1 ( 0 to 3 ) | 0.008 ( 0.001 to 0.026 ) | 0.005 ( 0.001 to 0.016 ) |  | 2 ( 0 to 6 ) | 0.008 ( 0.002 to 0.025 ) | 0.004 ( 0.001 to 0.013 ) | 0.15 (-0.02 to 0.32) |
| Suriname | 0 ( 0 to 0 ) | 0.078 ( 0.058 to 0.12 ) | 0.052 ( 0.038 to 0.077 ) |  | 0 ( 0 to 1 ) | 0.072 ( 0.046 to 0.101 ) | 0.075 ( 0.048 to 0.104 ) | 0.18 (0 to 0.36) |
| Sweden | 11 ( 10 to 13 ) | 0.079 ( 0.068 to 0.093 ) | 0.133 ( 0.114 to 0.156 ) |  | 24 ( 19 to 30 ) | 0.113 ( 0.089 to 0.141 ) | 0.236 ( 0.187 to 0.294 ) | 1.8 (1.51 to 2.09) |
| Switzerland | 11 ( 10 to 13 ) | 0.112 ( 0.096 to 0.131 ) | 0.164 ( 0.141 to 0.193 ) |  | 26 ( 20 to 32 ) | 0.142 ( 0.111 to 0.173 ) | 0.288 ( 0.224 to 0.354 ) | 0.78 (0.57 to 1) |
| Syrian Arab Republic | 1 ( 1 to 1 ) | 0.013 ( 0.009 to 0.019 ) | 0.006 ( 0.004 to 0.008 ) |  | 1 ( 1 to 2 ) | 0.012 ( 0.008 to 0.018 ) | 0.01 ( 0.006 to 0.015 ) | -0.38 (-0.5 to -0.25) |
| Taiwan (Province of China) | 9 ( 8 to 10 ) | 0.057 ( 0.051 to 0.064 ) | 0.043 ( 0.038 to 0.048 ) |  | 10 ( 9 to 13 ) | 0.03 ( 0.024 to 0.036 ) | 0.044 ( 0.036 to 0.054 ) | -1.22 (-1.61 to -0.83) |
| Tajikistan | 1 ( 0 to 2 ) | 0.01 ( 0.003 to 0.021 ) | 0.014 ( 0.003 to 0.032 ) |  | 2 ( 0 to 5 ) | 0.013 ( 0.003 to 0.037 ) | 0.016 ( 0.003 to 0.046 ) | 1.38 (1.06 to 1.71) |
| Thailand | 32 ( 21 to 43 ) | 0.077 ( 0.05 to 0.102 ) | 0.057 ( 0.036 to 0.075 ) |  | 47 ( 28 to 65 ) | 0.05 ( 0.032 to 0.07 ) | 0.071 ( 0.042 to 0.098 ) | -1.67 (-1.82 to -1.52) |
| Timor-Leste | 0 ( 0 to 1 ) | 0.049 ( 0.027 to 0.073 ) | 0.037 ( 0.016 to 0.073 ) |  | 1 ( 0 to 1 ) | 0.05 ( 0.026 to 0.076 ) | 0.039 ( 0.018 to 0.064 ) | 0.2 (0.06 to 0.34) |
| Togo | 8 ( 4 to 17 ) | 0.211 ( 0.126 to 0.368 ) | 0.213 ( 0.11 to 0.475 ) |  | 10 ( 5 to 21 ) | 0.154 ( 0.094 to 0.247 ) | 0.121 ( 0.063 to 0.248 ) | -1.03 (-1.17 to -0.9) |
| Tokelau | 0 ( 0 to 0 ) | 0.038 ( 0.025 to 0.06 ) | 0.036 ( 0.022 to 0.061 ) |  | 0 ( 0 to 0 ) | 0.396 ( 0.1 to 1.253 ) | 0.31 ( 0.084 to 0.967 ) | 1.91 (-0.28 to 4.14) |
| Tonga | 0 ( 0 to 0 ) | 0.031 ( 0.018 to 0.053 ) | 0.026 ( 0.013 to 0.058 ) |  | 0 ( 0 to 0 ) | 0.031 ( 0.017 to 0.057 ) | 0.03 ( 0.016 to 0.063 ) | 0.11 (-0.06 to 0.27) |
| Trinidad and Tobago | 1 ( 1 to 1 ) | 0.083 ( 0.065 to 0.117 ) | 0.069 ( 0.049 to 0.108 ) |  | 1 ( 1 to 1 ) | 0.063 ( 0.043 to 0.091 ) | 0.065 ( 0.048 to 0.088 ) | -0.49 (-0.86 to -0.11) |
| Tunisia | 6 ( 4 to 10 ) | 0.088 ( 0.06 to 0.132 ) | 0.073 ( 0.051 to 0.114 ) |  | 7 ( 4 to 11 ) | 0.059 ( 0.037 to 0.086 ) | 0.06 ( 0.037 to 0.09 ) | -1.45 (-1.53 to -1.37) |
| Turkey | 33 ( 24 to 48 ) | 0.081 ( 0.061 to 0.117 ) | 0.057 ( 0.042 to 0.084 ) |  | 48 ( 34 to 66 ) | 0.058 ( 0.041 to 0.077 ) | 0.057 ( 0.041 to 0.079 ) | -1.02 (-1.17 to -0.88) |
| Turkmenistan | 3 ( 2 to 4 ) | 0.126 ( 0.086 to 0.179 ) | 0.078 ( 0.053 to 0.112 ) |  | 4 ( 3 to 7 ) | 0.104 ( 0.065 to 0.165 ) | 0.083 ( 0.051 to 0.13 ) | -0.9 (-1.03 to -0.78) |
| Tuvalu | 0 ( 0 to 0 ) | 0.049 ( 0.03 to 0.084 ) | 0.048 ( 0.025 to 0.102 ) |  | 0 ( 0 to 0 ) | 0.037 ( 0.023 to 0.064 ) | 0.033 ( 0.02 to 0.061 ) | -0.75 (-0.91 to -0.59) |
| Uganda | 183 ( 117 to 255 ) | 1.884 ( 1.249 to 2.589 ) | 1.057 ( 0.677 to 1.473 ) |  | 374 ( 231 to 549 ) | 1.833 ( 1.152 to 2.634 ) | 0.863 ( 0.533 to 1.266 ) | -0.38 (-0.53 to -0.23) |
| Ukraine | 93 ( 76 to 113 ) | 0.133 ( 0.109 to 0.161 ) | 0.176 ( 0.145 to 0.214 ) |  | 90 ( 63 to 124 ) | 0.125 ( 0.088 to 0.169 ) | 0.209 ( 0.146 to 0.287 ) | -0.31 (-0.47 to -0.15) |
| United Arab Emirates | 1 ( 0 to 1 ) | 0.135 ( 0.086 to 0.232 ) | 0.035 ( 0.023 to 0.058 ) |  | 3 ( 2 to 5 ) | 0.122 ( 0.086 to 0.204 ) | 0.027 ( 0.019 to 0.053 ) | 2.01 (1.29 to 2.74) |
| United Kingdom | 182 ( 174 to 189 ) | 0.208 ( 0.198 to 0.216 ) | 0.318 ( 0.303 to 0.331 ) |  | 151 ( 138 to 162 ) | 0.12 ( 0.111 to 0.128 ) | 0.223 ( 0.203 to 0.239 ) | -1.31 (-1.73 to -0.88) |
| United Republic of Tanzania | 252 ( 169 to 381 ) | 1.457 ( 1.07 to 2.069 ) | 0.976 ( 0.652 to 1.476 ) |  | 413 ( 265 to 669 ) | 1.124 ( 0.757 to 1.855 ) | 0.706 ( 0.453 to 1.145 ) | -0.88 (-0.93 to -0.84) |
| United States of America | 315 ( 291 to 334 ) | 0.101 ( 0.094 to 0.107 ) | 0.124 ( 0.115 to 0.131 ) |  | 466 ( 422 to 501 ) | 0.083 ( 0.076 to 0.089 ) | 0.14 ( 0.127 to 0.151 ) | -0.67 (-0.92 to -0.42) |
| United States Virgin Islands | 0 ( 0 to 0 ) | 0.044 ( 0.024 to 0.083 ) | 0.04 ( 0.02 to 0.081 ) |  | 0 ( 0 to 0 ) | 0.02 ( 0.013 to 0.032 ) | 0.022 ( 0.015 to 0.031 ) | -2.47 (-2.71 to -2.22) |
| Uruguay | 4 ( 4 to 5 ) | 0.121 ( 0.1 to 0.148 ) | 0.14 ( 0.114 to 0.169 ) |  | 5 ( 4 to 7 ) | 0.094 ( 0.077 to 0.116 ) | 0.152 ( 0.122 to 0.194 ) | -0.96 (-1.17 to -0.75) |
| Uzbekistan | 10 ( 6 to 18 ) | 0.065 ( 0.038 to 0.104 ) | 0.048 ( 0.027 to 0.085 ) |  | 21 ( 14 to 30 ) | 0.074 ( 0.05 to 0.104 ) | 0.061 ( 0.04 to 0.087 ) | 0.33 (0.05 to 0.61) |
| Vanuatu | 0 ( 0 to 0 ) | 0.033 ( 0.02 to 0.054 ) | 0.026 ( 0.012 to 0.064 ) |  | 0 ( 0 to 0 ) | 0.034 ( 0.021 to 0.055 ) | 0.027 ( 0.015 to 0.056 ) | 0.24 (-0.02 to 0.49) |
| Venezuela (Bolivarian Republic of) | 20 ( 16 to 27 ) | 0.167 ( 0.145 to 0.198 ) | 0.108 ( 0.086 to 0.145 ) |  | 35 ( 27 to 46 ) | 0.128 ( 0.097 to 0.169 ) | 0.132 ( 0.1 to 0.172 ) | -0.92 (-1.03 to -0.81) |
| Viet Nam | 88 ( 52 to 155 ) | 0.119 ( 0.072 to 0.189 ) | 0.129 ( 0.076 to 0.227 ) |  | 70 ( 45 to 99 ) | 0.077 ( 0.051 to 0.11 ) | 0.07 ( 0.045 to 0.099 ) | -1.46 (-1.53 to -1.39) |
| Yemen | 1 ( 0 to 2 ) | 0.008 ( 0.001 to 0.027 ) | 0.004 ( 0.001 to 0.013 ) |  | 1 ( 0 to 4 ) | 0.009 ( 0.002 to 0.027 ) | 0.004 ( 0.001 to 0.013 ) | 0.59 (0.41 to 0.77) |
| Zambia | 74 ( 49 to 112 ) | 1.421 ( 1.045 to 2.078 ) | 0.927 ( 0.619 to 1.411 ) |  | 167 ( 91 to 284 ) | 1.679 ( 0.932 to 2.84 ) | 0.853 ( 0.466 to 1.457 ) | 0.7 (0.55 to 0.85) |
| Zimbabwe | 32 ( 20 to 43 ) | 0.678 ( 0.439 to 0.899 ) | 0.311 ( 0.197 to 0.411 ) |  | 96 ( 39 to 143 ) | 1.162 ( 0.5 to 1.68 ) | 0.614 ( 0.253 to 0.918 ) | 2.84 (2.03 to 3.66) |

**Supplementary Table S4: DALYs of Eye Cancer in 204 Countries and Territories in 1990 and 2021, with EAPC**

| **Location** | **1990** | | |  | **2021** | | | **EAPC(95%CI)** |
| --- | --- | --- | --- | --- | --- | --- | --- | --- |
|  | **Number(95%UI)** | **ASR(95%UI)** | **Rate(95%UI)** |  | **Number(95%UI)** | **ASR(95%UI)** | **Rate(95%UI)** |  |
| Afghanistan | 34 ( 5 to 125 ) | 0.362 ( 0.051 to 1.372 ) | 0.343 ( 0.055 to 1.252 ) |  | 88 ( 17 to 304 ) | 0.396 ( 0.077 to 1.372 ) | 0.282 ( 0.054 to 0.973 ) | 0.36 (0.27 to 0.46) |
| Albania | 254 ( 174 to 360 ) | 11.097 ( 7.699 to 15.7 ) | 7.68 ( 5.263 to 10.883 ) |  | 289 ( 190 to 470 ) | 7.195 ( 4.745 to 11.632 ) | 10.825 ( 7.13 to 17.608 ) | -1.23 (-1.35 to -1.12) |
| Algeria | 864 ( 503 to 1490 ) | 3.055 ( 1.933 to 4.763 ) | 3.416 ( 1.988 to 5.893 ) |  | 688 ( 461 to 1003 ) | 1.638 ( 1.102 to 2.381 ) | 1.556 ( 1.043 to 2.27 ) | -2.12 (-2.24 to -1.99) |
| American Samoa | 0 ( 0 to 1 ) | 0.844 ( 0.522 to 1.37 ) | 0.738 ( 0.403 to 1.429 ) |  | 0 ( 0 to 1 ) | 0.904 ( 0.5 to 1.591 ) | 0.833 ( 0.463 to 1.405 ) | 0.47 (0.04 to 0.91) |
| Andorra | 2 ( 1 to 3 ) | 3.388 ( 1.912 to 5.047 ) | 3.643 ( 2.024 to 5.48 ) |  | 3 ( 2 to 4 ) | 2.037 ( 1.111 to 3.136 ) | 3.379 ( 1.789 to 5.21 ) | -1.37 (-1.55 to -1.18) |
| Angola | 1211 ( 720 to 2278 ) | 10.29 ( 6.769 to 16.985 ) | 11.781 ( 7.002 to 22.172 ) |  | 2388 ( 1298 to 4567 ) | 7.5 ( 4.629 to 11.759 ) | 7.301 ( 3.969 to 13.965 ) | -1 (-1.07 to -0.92) |
| Antigua and Barbuda | 1 ( 1 to 2 ) | 2.641 ( 2.282 to 3.082 ) | 2.407 ( 2.07 to 2.838 ) |  | 4 ( 3 to 4 ) | 3.462 ( 3.079 to 3.837 ) | 3.993 ( 3.525 to 4.447 ) | 1.57 (0.88 to 2.26) |
| Argentina | 1235 ( 970 to 1615 ) | 3.732 ( 2.939 to 4.847 ) | 3.729 ( 2.928 to 4.878 ) |  | 868 ( 731 to 1052 ) | 1.739 ( 1.457 to 2.112 ) | 1.909 ( 1.608 to 2.313 ) | -1.99 (-2.23 to -1.75) |
| Armenia | 94 ( 63 to 135 ) | 3.237 ( 2.137 to 4.678 ) | 2.754 ( 1.827 to 3.957 ) |  | 129 ( 82 to 197 ) | 3.465 ( 2.111 to 5.425 ) | 4.318 ( 2.74 to 6.585 ) | 0.37 (0.17 to 0.57) |
| Australia | 886 ( 793 to 987 ) | 4.663 ( 4.167 to 5.178 ) | 5.256 ( 4.704 to 5.855 ) |  | 1187 ( 955 to 1442 ) | 3.013 ( 2.437 to 3.648 ) | 4.602 ( 3.702 to 5.591 ) | -1.48 (-1.6 to -1.36) |
| Austria | 453 ( 397 to 511 ) | 4.571 ( 4.004 to 5.133 ) | 5.829 ( 5.117 to 6.579 ) |  | 541 ( 435 to 660 ) | 3.582 ( 2.911 to 4.358 ) | 6.019 ( 4.843 to 7.346 ) | -0.09 (-0.41 to 0.23) |
| Azerbaijan | 248 ( 109 to 613 ) | 3.342 ( 1.648 to 7.509 ) | 3.38 ( 1.482 to 8.372 ) |  | 242 ( 106 to 536 ) | 2.691 ( 1.14 to 6.148 ) | 2.307 ( 1.007 to 5.103 ) | -0.7 (-0.95 to -0.45) |
| Bahamas | 16 ( 13 to 21 ) | 7.113 ( 5.881 to 9.261 ) | 6.162 ( 4.999 to 8.31 ) |  | 18 ( 14 to 22 ) | 4.524 ( 3.429 to 6.032 ) | 4.512 ( 3.497 to 5.723 ) | -1.23 (-1.77 to -0.7) |
| Bahrain | 2 ( 1 to 3 ) | 0.559 ( 0.383 to 1.005 ) | 0.356 ( 0.233 to 0.598 ) |  | 6 ( 2 to 11 ) | 0.607 ( 0.234 to 0.998 ) | 0.42 ( 0.16 to 0.728 ) | 1.14 (0.74 to 1.53) |
| Bangladesh | 12854 ( 6357 to 24675 ) | 7.971 ( 4.268 to 14.691 ) | 11.779 ( 5.826 to 22.612 ) |  | 6317 ( 3679 to 11087 ) | 4.284 ( 2.494 to 7.528 ) | 3.837 ( 2.235 to 6.734 ) | -2.08 (-2.21 to -1.95) |
| Barbados | 8 ( 5 to 15 ) | 3.59 ( 2.027 to 6.937 ) | 3.136 ( 1.897 to 5.734 ) |  | 8 ( 5 to 12 ) | 3.588 ( 1.791 to 6.693 ) | 2.575 ( 1.575 to 4.112 ) | 1.22 (0.41 to 2.03) |
| Belarus | 653 ( 539 to 815 ) | 5.58 ( 4.559 to 7.079 ) | 6.253 ( 5.159 to 7.806 ) |  | 925 ( 704 to 1253 ) | 7.025 ( 5.231 to 9.742 ) | 9.916 ( 7.551 to 13.439 ) | 0.35 (0.09 to 0.61) |
| Belgium | 555 ( 458 to 651 ) | 4.284 ( 3.59 to 5.018 ) | 5.566 ( 4.588 to 6.525 ) |  | 665 ( 530 to 814 ) | 3.581 ( 2.865 to 4.34 ) | 5.796 ( 4.622 to 7.101 ) | -0.26 (-0.57 to 0.05) |
| Belize | 5 ( 3 to 11 ) | 1.967 ( 1.018 to 3.792 ) | 2.926 ( 1.446 to 5.758 ) |  | 1 ( 1 to 2 ) | 0.303 ( 0.218 to 0.487 ) | 0.262 ( 0.188 to 0.424 ) | -5.63 (-7.05 to -4.19) |
| Benin | 1133 ( 486 to 2422 ) | 14.109 ( 7.245 to 28.049 ) | 23.358 ( 10.024 to 49.922 ) |  | 1790 ( 756 to 4375 ) | 9.147 ( 4.476 to 20.337 ) | 13.258 ( 5.602 to 32.408 ) | -1.35 (-1.53 to -1.16) |
| Bermuda | 0 ( 0 to 0 ) | 0.332 ( 0.277 to 0.397 ) | 0.341 ( 0.284 to 0.407 ) |  | 0 ( 0 to 0 ) | 0.259 ( 0.2 to 0.342 ) | 0.48 ( 0.372 to 0.628 ) | -0.84 (-1.1 to -0.59) |
| Bhutan | 55 ( 19 to 132 ) | 6.672 ( 2.904 to 14.904 ) | 8.708 ( 3.051 to 21.004 ) |  | 32 ( 16 to 57 ) | 5.081 ( 2.505 to 9.175 ) | 4.177 ( 2.062 to 7.517 ) | -0.87 (-1.11 to -0.64) |
| Bolivia (Plurinational State of) | 1255 ( 638 to 2590 ) | 15.28 ( 8.751 to 29.765 ) | 19.669 ( 10.003 to 40.592 ) |  | 1076 ( 586 to 1982 ) | 9.647 ( 5.584 to 17.382 ) | 9.119 ( 4.965 to 16.804 ) | -1.56 (-1.66 to -1.45) |
| Bosnia and Herzegovina | 194 ( 139 to 270 ) | 4.64 ( 3.339 to 6.439 ) | 4.322 ( 3.086 to 6.007 ) |  | 196 ( 135 to 272 ) | 3.415 ( 2.356 to 4.731 ) | 5.931 ( 4.078 to 8.221 ) | -0.98 (-1.08 to -0.88) |
| Botswana | 109 ( 62 to 196 ) | 10.189 ( 6.015 to 19.415 ) | 8.275 ( 4.737 to 14.872 ) |  | 175 ( 95 to 321 ) | 8.755 ( 4.96 to 16.106 ) | 7.304 ( 3.975 to 13.397 ) | -0.38 (-0.5 to -0.26) |
| Brazil | 8327 ( 7121 to 9716 ) | 6.047 ( 5.264 to 6.9 ) | 5.607 ( 4.795 to 6.542 ) |  | 7257 ( 6476 to 8060 ) | 3.239 ( 2.855 to 3.652 ) | 3.293 ( 2.939 to 3.658 ) | -1.84 (-2.07 to -1.6) |
| Brunei Darussalam | 28 ( 14 to 56 ) | 10.404 ( 5.959 to 18.608 ) | 10.915 ( 5.344 to 21.511 ) |  | 28 ( 19 to 42 ) | 7.576 ( 4.884 to 12.32 ) | 6.098 ( 4.105 to 9.418 ) | -0.43 (-0.64 to -0.23) |
| Bulgaria | 545 ( 422 to 699 ) | 5.108 ( 3.97 to 6.58 ) | 6.28 ( 4.864 to 8.057 ) |  | 452 ( 326 to 620 ) | 3.8 ( 2.749 to 5.247 ) | 6.658 ( 4.803 to 9.141 ) | -1.04 (-1.23 to -0.84) |
| Burkina Faso | 2188 ( 993 to 4937 ) | 14.566 ( 7.419 to 30.253 ) | 22.968 ( 10.419 to 51.816 ) |  | 3131 ( 1428 to 6911 ) | 9.305 ( 4.89 to 18.889 ) | 13.755 ( 6.273 to 30.361 ) | -1.27 (-1.65 to -0.89) |
| Burundi | 3202 ( 1919 to 5118 ) | 57.939 ( 40.13 to 83.983 ) | 57.651 ( 34.563 to 92.161 ) |  | 4333 ( 2728 to 7111 ) | 41.774 ( 27.38 to 64.277 ) | 32.776 ( 20.633 to 53.786 ) | -0.87 (-1 to -0.74) |
| Cabo Verde | 29 ( 13 to 61 ) | 5.903 ( 3.106 to 11.682 ) | 8.077 ( 3.659 to 17.273 ) |  | 26 ( 14 to 50 ) | 5.903 ( 3.122 to 11.125 ) | 4.628 ( 2.45 to 8.88 ) | 0.07 (-0.28 to 0.42) |
| Cambodia | 325 ( 123 to 640 ) | 2.788 ( 1.283 to 4.612 ) | 3.164 ( 1.195 to 6.228 ) |  | 362 ( 165 to 619 ) | 2.33 ( 1.106 to 3.819 ) | 2.121 ( 0.966 to 3.632 ) | -0.67 (-0.73 to -0.61) |
| Cameroon | 1842 ( 837 to 3983 ) | 11.846 ( 6.209 to 23.089 ) | 17.651 ( 8.02 to 38.164 ) |  | 2854 ( 1155 to 6158 ) | 7.255 ( 3.492 to 14.402 ) | 8.982 ( 3.635 to 19.378 ) | -1.15 (-1.35 to -0.94) |
| Canada | 1314 ( 1142 to 1496 ) | 4.326 ( 3.774 to 4.944 ) | 4.82 ( 4.191 to 5.49 ) |  | 1673 ( 1397 to 1965 ) | 2.756 ( 2.328 to 3.237 ) | 4.466 ( 3.729 to 5.245 ) | -1.33 (-1.52 to -1.14) |
| Central African Republic | 359 ( 213 to 622 ) | 11.956 ( 7.956 to 18.921 ) | 13.157 ( 7.786 to 22.794 ) |  | 597 ( 321 to 1131 ) | 10.944 ( 6.601 to 17.908 ) | 10.895 ( 5.851 to 20.616 ) | -0.22 (-0.32 to -0.11) |
| Chad | 1222 ( 551 to 2653 ) | 12.451 ( 6.485 to 24.955 ) | 20.27 ( 9.147 to 44.021 ) |  | 2870 ( 1288 to 6199 ) | 9.788 ( 4.949 to 19.487 ) | 16.171 ( 7.259 to 34.927 ) | -0.74 (-0.81 to -0.66) |
| Chile | 430 ( 361 to 503 ) | 3.738 ( 3.148 to 4.372 ) | 3.237 ( 2.718 to 3.785 ) |  | 386 ( 321 to 464 ) | 1.757 ( 1.453 to 2.154 ) | 2.053 ( 1.705 to 2.469 ) | -2.44 (-2.72 to -2.16) |
| China | 38265 ( 22354 to 53418 ) | 3.665 ( 2.185 to 5.038 ) | 3.253 ( 1.9 to 4.541 ) |  | 22668 ( 12665 to 29517 ) | 1.686 ( 0.929 to 2.233 ) | 1.593 ( 0.89 to 2.075 ) | -1.84 (-2.14 to -1.55) |
| Colombia | 1920 ( 1556 to 2430 ) | 6.242 ( 5.26 to 7.527 ) | 5.91 ( 4.79 to 7.479 ) |  | 1480 ( 1155 to 1894 ) | 3.207 ( 2.445 to 4.205 ) | 3.017 ( 2.353 to 3.861 ) | -0.82 (-1.31 to -0.32) |
| Comoros | 242 ( 146 to 423 ) | 54.256 ( 38.136 to 81.823 ) | 52.387 ( 31.579 to 91.445 ) |  | 312 ( 188 to 517 ) | 47.484 ( 29.929 to 79.44 ) | 41.881 ( 25.236 to 69.422 ) | -0.64 (-0.78 to -0.5) |
| Congo | 249 ( 145 to 461 ) | 10.365 ( 6.905 to 16.915 ) | 10.365 ( 6.05 to 19.174 ) |  | 329 ( 204 to 538 ) | 7.331 ( 4.776 to 11.192 ) | 6.102 ( 3.777 to 9.97 ) | -1.09 (-1.21 to -0.98) |
| Cook Islands | 0 ( 0 to 1 ) | 1.441 ( 0.74 to 3.276 ) | 1.434 ( 0.654 to 3.634 ) |  | 0 ( 0 to 1 ) | 3.357 ( 1.154 to 9.899 ) | 2.421 ( 0.937 to 6.644 ) | 0 (-1 to 1) |
| Costa Rica | 194 ( 150 to 263 ) | 6.875 ( 5.682 to 8.492 ) | 6.388 ( 4.938 to 8.636 ) |  | 119 ( 96 to 149 ) | 2.525 ( 1.989 to 3.305 ) | 2.501 ( 2.031 to 3.132 ) | -2.59 (-3.13 to -2.05) |
| Coted'Ivoire | 726 ( 378 to 1540 ) | 5.448 ( 3.413 to 9.058 ) | 5.956 ( 3.095 to 12.624 ) |  | 1195 ( 581 to 2397 ) | 4.588 ( 2.656 to 7.542 ) | 4.289 ( 2.085 to 8.604 ) | -0.3 (-0.48 to -0.12) |
| Croatia | 345 ( 282 to 416 ) | 5.861 ( 4.783 to 7.082 ) | 7.094 ( 5.802 to 8.557 ) |  | 276 ( 205 to 362 ) | 3.397 ( 2.513 to 4.426 ) | 6.551 ( 4.877 to 8.603 ) | -1.56 (-1.79 to -1.33) |
| Cuba | 631 ( 530 to 760 ) | 6.301 ( 5.243 to 7.609 ) | 5.817 ( 4.882 to 7.004 ) |  | 652 ( 521 to 805 ) | 3.745 ( 3.004 to 4.578 ) | 5.782 ( 4.621 to 7.146 ) | -1.08 (-1.33 to -0.83) |
| Cyprus | 29 ( 23 to 43 ) | 3.696 ( 2.88 to 5.615 ) | 3.721 ( 2.907 to 5.569 ) |  | 52 ( 33 to 71 ) | 2.676 ( 1.712 to 3.617 ) | 3.811 ( 2.464 to 5.213 ) | -0.7 (-0.83 to -0.58) |
| Czechia | 764 ( 586 to 971 ) | 5.964 ( 4.641 to 7.482 ) | 7.424 ( 5.69 to 9.433 ) |  | 619 ( 430 to 868 ) | 3.316 ( 2.386 to 4.551 ) | 5.819 ( 4.042 to 8.161 ) | -1.72 (-1.9 to -1.54) |
| Democratic People's Republic of Korea | 601 ( 364 to 1040 ) | 2.867 ( 1.755 to 4.772 ) | 2.921 ( 1.769 to 5.048 ) |  | 647 ( 384 to 1013 ) | 2.843 ( 1.587 to 4.935 ) | 2.451 ( 1.453 to 3.837 ) | 0.07 (-0.04 to 0.19) |
| Democratic Republic of the Congo | 3922 ( 2320 to 6470 ) | 9.34 ( 6.259 to 13.76 ) | 10.28 ( 6.081 to 16.96 ) |  | 5716 ( 3481 to 9797 ) | 7.639 ( 4.942 to 12.86 ) | 6.349 ( 3.867 to 10.883 ) | -0.4 (-0.52 to -0.27) |
| Denmark | 407 ( 339 to 496 ) | 6.189 ( 5.258 to 7.404 ) | 7.919 ( 6.597 to 9.638 ) |  | 456 ( 364 to 564 ) | 4.724 ( 3.812 to 5.809 ) | 7.788 ( 6.219 to 9.637 ) | -0.93 (-1.06 to -0.79) |
| Djibouti | 199 ( 114 to 322 ) | 53.11 ( 34.734 to 81.94 ) | 48.043 ( 27.602 to 77.729 ) |  | 449 ( 245 to 759 ) | 43.243 ( 25.436 to 72.028 ) | 35.638 ( 19.493 to 60.291 ) | -0.55 (-0.8 to -0.31) |
| Dominica | 3 ( 2 to 5 ) | 4.639 ( 2.584 to 8.28 ) | 3.904 ( 2.163 to 6.923 ) |  | 4 ( 2 to 7 ) | 4.944 ( 2.547 to 8.385 ) | 5.824 ( 3.006 to 9.785 ) | 0.36 (0.27 to 0.44) |
| Dominican Republic | 186 ( 96 to 308 ) | 2.585 ( 1.457 to 3.849 ) | 2.603 ( 1.345 to 4.312 ) |  | 174 ( 106 to 253 ) | 1.667 ( 1.015 to 2.428 ) | 1.579 ( 0.966 to 2.294 ) | -0.33 (-0.7 to 0.05) |
| Ecuador | 855 ( 593 to 1217 ) | 8.46 ( 6.304 to 11.258 ) | 8.564 ( 5.945 to 12.197 ) |  | 1091 ( 769 to 1564 ) | 6.538 ( 4.629 to 9.366 ) | 6.04 ( 4.258 to 8.657 ) | -0.21 (-0.58 to 0.16) |
| Egypt | 679 ( 382 to 1397 ) | 1.477 ( 0.952 to 2.884 ) | 1.226 ( 0.69 to 2.525 ) |  | 894 ( 529 to 1815 ) | 1.1 ( 0.689 to 2.037 ) | 0.846 ( 0.5 to 1.718 ) | -0.67 (-1.27 to -0.08) |
| El Salvador | 194 ( 124 to 327 ) | 3.926 ( 2.654 to 5.989 ) | 3.655 ( 2.332 to 6.172 ) |  | 182 ( 124 to 257 ) | 2.866 ( 1.962 to 4.055 ) | 2.817 ( 1.92 to 3.983 ) | -1.04 (-1.16 to -0.93) |
| Equatorial Guinea | 52 ( 28 to 100 ) | 10.711 ( 6.517 to 17.469 ) | 12.188 ( 6.691 to 23.681 ) |  | 58 ( 30 to 112 ) | 5.405 ( 3.193 to 9.382 ) | 3.837 ( 1.979 to 7.413 ) | -2.65 (-2.93 to -2.36) |
| Eritrea | 1967 ( 1047 to 3406 ) | 62.622 ( 42.411 to 95.506 ) | 57.756 ( 30.731 to 99.997 ) |  | 3148 ( 1738 to 5318 ) | 56.016 ( 35.148 to 91.51 ) | 47.709 ( 26.342 to 80.599 ) | -0.45 (-0.55 to -0.35) |
| Estonia | 115 ( 87 to 152 ) | 6.08 ( 4.629 to 8.067 ) | 7.328 ( 5.552 to 9.664 ) |  | 129 ( 93 to 175 ) | 5.655 ( 4.112 to 7.717 ) | 9.831 ( 7.129 to 13.386 ) | -1.26 (-1.63 to -0.88) |
| Eswatini | 72 ( 39 to 123 ) | 10.932 ( 6.397 to 20.846 ) | 8.914 ( 4.889 to 15.304 ) |  | 104 ( 53 to 196 ) | 11.311 ( 5.917 to 22.717 ) | 9 ( 4.569 to 16.97 ) | 0.39 (0.13 to 0.66) |
| Ethiopia | 16599 ( 9783 to 26791 ) | 33.008 ( 20.637 to 47.592 ) | 32.824 ( 19.346 to 52.98 ) |  | 17803 ( 11770 to 25652 ) | 20.531 ( 13.651 to 27.199 ) | 16.342 ( 10.805 to 23.547 ) | -1.81 (-1.97 to -1.64) |
| Fiji | 16 ( 5 to 40 ) | 2.377 ( 0.894 to 5.626 ) | 2.138 ( 0.693 to 5.253 ) |  | 21 ( 7 to 50 ) | 2.349 ( 0.773 to 5.535 ) | 2.246 ( 0.718 to 5.378 ) | 0.11 (-0.23 to 0.45) |
| Finland | 306 ( 262 to 354 ) | 4.873 ( 4.187 to 5.609 ) | 6.115 ( 5.236 to 7.074 ) |  | 699 ( 570 to 861 ) | 7.367 ( 6.1 to 8.98 ) | 12.63 ( 10.295 to 15.558 ) | 1.25 (1.01 to 1.49) |
| France | 3954 ( 3544 to 4372 ) | 5.709 ( 5.154 to 6.302 ) | 6.844 ( 6.134 to 7.568 ) |  | 4692 ( 3753 to 5857 ) | 4.43 ( 3.632 to 5.446 ) | 7.067 ( 5.652 to 8.822 ) | -0.41 (-0.55 to -0.27) |
| Gabon | 76 ( 47 to 128 ) | 8.075 ( 5.478 to 12.818 ) | 7.759 ( 4.784 to 13.054 ) |  | 96 ( 57 to 160 ) | 6.358 ( 3.915 to 10.34 ) | 5.297 ( 3.163 to 8.837 ) | -0.62 (-0.72 to -0.53) |
| Gambia | 195 ( 103 to 380 ) | 16.454 ( 9.717 to 27.818 ) | 19.895 ( 10.451 to 38.758 ) |  | 390 ( 203 to 786 ) | 16.218 ( 9.912 to 28.199 ) | 16.287 ( 8.461 to 32.827 ) | -0.17 (-0.44 to 0.1) |
| Georgia | 166 ( 132 to 204 ) | 2.806 ( 2.227 to 3.497 ) | 3.001 ( 2.39 to 3.696 ) |  | 341 ( 273 to 439 ) | 7.365 ( 5.618 to 10.266 ) | 9.464 ( 7.568 to 12.16 ) | 4.06 (3.61 to 4.51) |
| Germany | 4475 ( 3757 to 5287 ) | 4.22 ( 3.567 to 4.945 ) | 5.598 ( 4.699 to 6.614 ) |  | 7050 ( 5902 to 8293 ) | 4.514 ( 3.802 to 5.237 ) | 8.258 ( 6.913 to 9.714 ) | 0.57 (0.4 to 0.74) |
| Ghana | 1854 ( 722 to 4452 ) | 7.469 ( 2.982 to 17.769 ) | 12.384 ( 4.82 to 29.734 ) |  | 2113 ( 754 to 4996 ) | 4.856 ( 1.769 to 11.222 ) | 6.169 ( 2.203 to 14.589 ) | -0.92 (-1.2 to -0.63) |
| Greece | 419 ( 374 to 470 ) | 3.012 ( 2.69 to 3.355 ) | 4.034 ( 3.596 to 4.522 ) |  | 589 ( 512 to 672 ) | 3.089 ( 2.703 to 3.528 ) | 5.785 ( 5.029 to 6.606 ) | 0.44 (0.23 to 0.66) |
| Greenland | 1 ( 0 to 2 ) | 2.33 ( 0.929 to 3.582 ) | 1.908 ( 0.734 to 3.026 ) |  | 1 ( 0 to 1 ) | 0.822 ( 0.555 to 1.211 ) | 0.994 ( 0.618 to 1.389 ) | -2.65 (-3.08 to -2.22) |
| Grenada | 2 ( 1 to 2 ) | 2.226 ( 1.827 to 2.722 ) | 1.939 ( 1.57 to 2.412 ) |  | 2 ( 2 to 2 ) | 1.759 ( 1.402 to 2.132 ) | 1.929 ( 1.536 to 2.337 ) | -0.34 (-0.62 to -0.04) |
| Guam | 1 ( 0 to 3 ) | 0.778 ( 0.352 to 1.872 ) | 0.729 ( 0.3 to 1.873 ) |  | 1 ( 0 to 3 ) | 0.692 ( 0.275 to 1.882 ) | 0.661 ( 0.281 to 1.611 ) | 1.32 (0.88 to 1.76) |
| Guatemala | 748 ( 414 to 1350 ) | 7.907 ( 5.582 to 11.94 ) | 8.923 ( 4.932 to 16.097 ) |  | 633 ( 473 to 864 ) | 4.606 ( 3.53 to 6.082 ) | 4.017 ( 2.998 to 5.477 ) | -1.46 (-1.58 to -1.34) |
| Guinea | 2283 ( 1124 to 4673 ) | 22.287 ( 11.449 to 44.277 ) | 38.094 ( 18.755 to 77.956 ) |  | 2830 ( 1202 to 6294 ) | 14.49 ( 6.842 to 30.381 ) | 21.072 ( 8.949 to 46.867 ) | -1.03 (-1.23 to -0.84) |
| Guinea-Bissau | 256 ( 116 to 589 ) | 17.001 ( 8.532 to 36.023 ) | 25.401 ( 11.477 to 58.456 ) |  | 242 ( 106 to 548 ) | 9.188 ( 4.739 to 18.581 ) | 11.732 ( 5.133 to 26.537 ) | -1.45 (-1.77 to -1.12) |
| Guyana | 5 ( 4 to 8 ) | 0.738 ( 0.561 to 1.018 ) | 0.649 ( 0.455 to 0.992 ) |  | 27 ( 19 to 38 ) | 3.814 ( 2.74 to 5.329 ) | 3.546 ( 2.55 to 4.983 ) | 3.97 (1.76 to 6.23) |
| Haiti | 147 ( 74 to 233 ) | 2.485 ( 1.418 to 3.678 ) | 2.302 ( 1.156 to 3.644 ) |  | 212 ( 108 to 336 ) | 2.005 ( 1.055 to 2.969 ) | 1.651 ( 0.837 to 2.608 ) | -0.56 (-0.61 to -0.51) |
| Honduras | 357 ( 209 to 680 ) | 6.58 ( 4.257 to 10.946 ) | 7.583 ( 4.446 to 14.437 ) |  | 455 ( 292 to 700 ) | 5.35 ( 3.68 to 7.715 ) | 4.5 ( 2.89 to 6.925 ) | -0.7 (-0.77 to -0.63) |
| Hungary | 979 ( 814 to 1178 ) | 7.455 ( 6.216 to 9 ) | 9.417 ( 7.833 to 11.334 ) |  | 725 ( 529 to 983 ) | 4.376 ( 3.222 to 5.954 ) | 7.558 ( 5.517 to 10.239 ) | -1.53 (-1.68 to -1.38) |
| Iceland | 11 ( 10 to 13 ) | 4.187 ( 3.664 to 4.756 ) | 4.381 ( 3.827 to 4.984 ) |  | 20 ( 16 to 24 ) | 4.263 ( 3.431 to 5.196 ) | 5.698 ( 4.667 to 6.912 ) | 0.24 (-0.15 to 0.63) |
| India | 64722 ( 31776 to 95839 ) | 6.056 ( 3.111 to 8.761 ) | 7.587 ( 3.725 to 11.235 ) |  | 46025 ( 29325 to 64199 ) | 3.951 ( 2.505 to 5.556 ) | 3.254 ( 2.073 to 4.539 ) | -1.48 (-1.67 to -1.29) |
| Indonesia | 3425 ( 1499 to 5131 ) | 1.951 ( 0.957 to 2.704 ) | 1.851 ( 0.81 to 2.774 ) |  | 4395 ( 2107 to 6095 ) | 1.777 ( 0.835 to 2.497 ) | 1.576 ( 0.756 to 2.185 ) | -0.23 (-0.37 to -0.08) |
| Iran (Islamic Republic of) | 51 ( 10 to 109 ) | 0.088 ( 0.02 to 0.167 ) | 0.09 ( 0.018 to 0.19 ) |  | 73 ( 21 to 105 ) | 0.096 ( 0.028 to 0.14 ) | 0.085 ( 0.025 to 0.123 ) | 1.73 (1.22 to 2.25) |
| Iraq | 609 ( 364 to 1069 ) | 2.884 ( 2.021 to 4.409 ) | 3.305 ( 1.979 to 5.804 ) |  | 1001 ( 622 to 1583 ) | 2.684 ( 1.725 to 4.023 ) | 2.427 ( 1.508 to 3.841 ) | -0.45 (-0.59 to -0.31) |
| Ireland | 243 ( 214 to 276 ) | 6.247 ( 5.523 to 7.084 ) | 6.747 ( 5.948 to 7.674 ) |  | 247 ( 194 to 306 ) | 3.486 ( 2.76 to 4.277 ) | 5.005 ( 3.933 to 6.202 ) | -1.3 (-1.51 to -1.1) |
| Israel | 109 ( 87 to 133 ) | 2.256 ( 1.806 to 2.756 ) | 2.191 ( 1.745 to 2.671 ) |  | 198 ( 166 to 236 ) | 1.769 ( 1.484 to 2.145 ) | 2.065 ( 1.725 to 2.46 ) | -0.52 (-0.61 to -0.42) |
| Italy | 3178 ( 2909 to 3466 ) | 4.262 ( 3.916 to 4.644 ) | 5.596 ( 5.122 to 6.102 ) |  | 4475 ( 3840 to 5202 ) | 4.095 ( 3.596 to 4.706 ) | 7.482 ( 6.42 to 8.697 ) | -0.2 (-0.38 to -0.02) |
| Jamaica | 79 ( 45 to 146 ) | 3.278 ( 2.027 to 5.686 ) | 3.345 ( 1.922 to 6.19 ) |  | 52 ( 36 to 74 ) | 1.857 ( 1.275 to 2.805 ) | 1.843 ( 1.295 to 2.628 ) | -1.64 (-2.31 to -0.96) |
| Japan | 1344 ( 1270 to 1435 ) | 1.097 ( 1.025 to 1.179 ) | 1.068 ( 1.01 to 1.14 ) |  | 1888 ( 1694 to 2094 ) | 0.95 ( 0.856 to 1.054 ) | 1.478 ( 1.327 to 1.64 ) | -0.24 (-0.69 to 0.21) |
| Jordan | 92 ( 59 to 147 ) | 2.608 ( 1.836 to 3.632 ) | 2.476 ( 1.589 to 3.939 ) |  | 163 ( 105 to 232 ) | 1.688 ( 1.077 to 2.389 ) | 1.326 ( 0.856 to 1.879 ) | -1.7 (-1.93 to -1.47) |
| Kazakhstan | 1001 ( 557 to 1793 ) | 6.221 ( 3.722 to 10.758 ) | 6.104 ( 3.395 to 10.935 ) |  | 612 ( 369 to 977 ) | 3.239 ( 1.953 to 5.162 ) | 3.227 ( 1.946 to 5.156 ) | -2.44 (-3.03 to -1.85) |
| Kenya | 22624 ( 13245 to 32537 ) | 75.389 ( 50.042 to 98.512 ) | 97.742 ( 57.22 to 140.565 ) |  | 28871 ( 20525 to 40391 ) | 65.71 ( 49.606 to 85.922 ) | 57.669 ( 40.998 to 80.68 ) | 0.16 (-0.07 to 0.39) |
| Kiribati | 3 ( 1 to 6 ) | 3.39 ( 1.758 to 6.451 ) | 3.423 ( 1.462 to 8.36 ) |  | 3 ( 2 to 7 ) | 3.162 ( 1.566 to 6.073 ) | 2.88 ( 1.336 to 5.873 ) | -0.57 (-0.75 to -0.38) |
| Kuwait | 33 ( 25 to 45 ) | 2.272 ( 1.824 to 2.851 ) | 1.916 ( 1.458 to 2.594 ) |  | 28 ( 22 to 36 ) | 0.772 ( 0.612 to 0.994 ) | 0.61 ( 0.482 to 0.777 ) | -2.47 (-4.65 to -0.24) |
| Kyrgyzstan | 320 ( 188 to 601 ) | 7.263 ( 4.964 to 11.304 ) | 7.168 ( 4.212 to 13.454 ) |  | 353 ( 234 to 563 ) | 5.424 ( 3.747 to 8.266 ) | 5.149 ( 3.41 to 8.203 ) | -0.34 (-1.66 to 1.01) |
| Lao People's Democratic Republic | 136 ( 50 to 285 ) | 2.827 ( 1.222 to 5.098 ) | 3.262 ( 1.201 to 6.833 ) |  | 144 ( 58 to 283 ) | 2.102 ( 0.908 to 3.841 ) | 1.957 ( 0.787 to 3.84 ) | -0.93 (-1.03 to -0.84) |
| Latvia | 154 ( 118 to 196 ) | 4.853 ( 3.693 to 6.291 ) | 5.794 ( 4.424 to 7.391 ) |  | 175 ( 130 to 230 ) | 5.249 ( 3.999 to 6.858 ) | 9.342 ( 6.933 to 12.28 ) | -0.27 (-0.54 to 0) |
| Lebanon | 82 ( 60 to 122 ) | 3.144 ( 2.305 to 4.779 ) | 2.749 ( 1.999 to 4.066 ) |  | 135 ( 95 to 201 ) | 2.362 ( 1.675 to 3.435 ) | 2.43 ( 1.721 to 3.635 ) | -0.75 (-0.81 to -0.68) |
| Lesotho | 141 ( 77 to 256 ) | 10.117 ( 5.851 to 19.332 ) | 9.218 ( 5.031 to 16.728 ) |  | 191 ( 100 to 359 ) | 12.578 ( 6.698 to 24.836 ) | 10.21 ( 5.324 to 19.154 ) | 1.09 (0.9 to 1.28) |
| Liberia | 417 ( 181 to 941 ) | 11.229 ( 5.444 to 23.74 ) | 16.935 ( 7.346 to 38.232 ) |  | 388 ( 172 to 926 ) | 6.34 ( 3.171 to 13.659 ) | 7.111 ( 3.143 to 16.956 ) | -1.82 (-2.38 to -1.26) |
| Libya | 155 ( 80 to 315 ) | 3.022 ( 1.757 to 5.573 ) | 3.676 ( 1.906 to 7.469 ) |  | 114 ( 65 to 182 ) | 2.179 ( 1.27 to 3.658 ) | 1.657 ( 0.95 to 2.656 ) | -1.13 (-1.24 to -1.02) |
| Lithuania | 252 ( 196 to 320 ) | 6.011 ( 4.629 to 7.705 ) | 6.871 ( 5.332 to 8.699 ) |  | 332 ( 258 to 429 ) | 6.823 ( 5.313 to 8.933 ) | 12.152 ( 9.464 to 15.729 ) | 0.04 (-0.17 to 0.26) |
| Luxembourg | 22 ( 20 to 24 ) | 4.264 ( 3.878 to 4.711 ) | 5.642 ( 5.136 to 6.235 ) |  | 26 ( 22 to 31 ) | 2.649 ( 2.223 to 3.124 ) | 4.08 ( 3.412 to 4.811 ) | -1.05 (-1.24 to -0.86) |
| Madagascar | 5841 ( 3499 to 9669 ) | 49.453 ( 34.956 to 73.526 ) | 49.082 ( 29.399 to 81.246 ) |  | 9431 ( 5242 to 16366 ) | 38.491 ( 25.094 to 61.261 ) | 33.023 ( 18.354 to 57.307 ) | -0.81 (-0.89 to -0.74) |
| Malawi | 13113 ( 8557 to 21283 ) | 136.223 ( 93.859 to 188.154 ) | 133.732 ( 87.264 to 217.045 ) |  | 18960 ( 10498 to 31649 ) | 129.182 ( 77.066 to 190.59 ) | 97.494 ( 53.98 to 162.737 ) | -0.13 (-0.28 to 0.03) |
| Malaysia | 621 ( 262 to 1255 ) | 3.213 ( 1.479 to 5.998 ) | 3.517 ( 1.481 to 7.106 ) |  | 560 ( 292 to 911 ) | 2.041 ( 1.045 to 3.403 ) | 1.76 ( 0.918 to 2.864 ) | -1.32 (-1.65 to -0.98) |
| Maldives | 2 ( 1 to 3 ) | 0.845 ( 0.539 to 1.257 ) | 0.774 ( 0.454 to 1.312 ) |  | 2 ( 1 to 3 ) | 0.472 ( 0.299 to 0.651 ) | 0.374 ( 0.235 to 0.54 ) | -1.99 (-2.08 to -1.91) |
| Mali | 4377 ( 2346 to 8202 ) | 40.809 ( 25.463 to 64.623 ) | 50.527 ( 27.079 to 94.677 ) |  | 6351 ( 2962 to 14813 ) | 27.352 ( 16.092 to 48.996 ) | 26.346 ( 12.287 to 61.456 ) | -1.17 (-1.29 to -1.06) |
| Malta | 14 ( 12 to 16 ) | 3.401 ( 2.963 to 3.832 ) | 3.87 ( 3.372 to 4.355 ) |  | 23 ( 18 to 29 ) | 2.911 ( 2.302 to 3.599 ) | 5.241 ( 4.162 to 6.45 ) | 0.07 (-0.13 to 0.27) |
| Marshall Islands | 1 ( 0 to 2 ) | 1.669 ( 0.915 to 3.513 ) | 1.797 ( 0.709 to 4.711 ) |  | 1 ( 0 to 2 ) | 1.903 ( 0.846 to 4.42 ) | 1.784 ( 0.729 to 4.328 ) | 0.37 (0.15 to 0.6) |
| Mauritania | 324 ( 150 to 754 ) | 10.994 ( 5.843 to 23.45 ) | 15.768 ( 7.314 to 36.675 ) |  | 312 ( 146 to 666 ) | 5.904 ( 3.151 to 11.391 ) | 7.106 ( 3.315 to 15.143 ) | -2.26 (-2.4 to -2.11) |
| Mauritius | 7 ( 6 to 8 ) | 0.721 ( 0.62 to 0.873 ) | 0.607 ( 0.516 to 0.753 ) |  | 21 ( 19 to 24 ) | 1.415 ( 1.234 to 1.635 ) | 1.663 ( 1.473 to 1.86 ) | 3.63 (2.18 to 5.09) |
| Mexico | 6059 ( 5279 to 6950 ) | 6.663 ( 5.973 to 7.471 ) | 7.095 ( 6.182 to 8.139 ) |  | 4084 ( 3396 to 4938 ) | 3.559 ( 2.912 to 4.374 ) | 3.159 ( 2.627 to 3.82 ) | -2.13 (-2.34 to -1.91) |
| Micronesia (Federated States of) | 2 ( 1 to 7 ) | 2.084 ( 1.052 to 4.55 ) | 2.354 ( 0.904 to 6.346 ) |  | 2 ( 1 to 4 ) | 1.743 ( 0.896 to 3.986 ) | 1.525 ( 0.754 to 3.66 ) | -0.25 (-0.43 to -0.07) |
| Monaco | 0 ( 0 to 0 ) | 0.271 ( 0.156 to 0.378 ) | 0.453 ( 0.261 to 0.632 ) |  | 0 ( 0 to 0 ) | 0.286 ( 0.19 to 0.395 ) | 0.463 ( 0.303 to 0.638 ) | -0.18 (-0.31 to -0.05) |
| Mongolia | 106 ( 47 to 221 ) | 4.945 ( 2.881 to 8.637 ) | 4.914 ( 2.195 to 10.264 ) |  | 152 ( 85 to 291 ) | 4.55 ( 2.7 to 8.092 ) | 4.556 ( 2.556 to 8.723 ) | -0.17 (-0.3 to -0.03) |
| Montenegro | 10 ( 7 to 14 ) | 1.643 ( 1.099 to 2.173 ) | 1.641 ( 1.098 to 2.176 ) |  | 14 ( 9 to 18 ) | 1.537 ( 1.002 to 2.07 ) | 2.205 ( 1.446 to 2.985 ) | -0.26 (-0.4 to -0.11) |
| Morocco | 3377 ( 1676 to 6414 ) | 10.58 ( 5.625 to 19.354 ) | 13.318 ( 6.61 to 25.296 ) |  | 2287 ( 1390 to 3873 ) | 6.822 ( 4.066 to 11.601 ) | 6.152 ( 3.738 to 10.419 ) | -1.1 (-1.27 to -0.92) |
| Mozambique | 9715 ( 5889 to 16842 ) | 66.845 ( 46.47 to 98.682 ) | 72.72 ( 44.08 to 126.06 ) |  | 15386 ( 9261 to 24631 ) | 57.215 ( 38.336 to 89.428 ) | 49.517 ( 29.803 to 79.269 ) | -0.19 (-0.33 to -0.06) |
| Myanmar | 1237 ( 472 to 2671 ) | 3.028 ( 1.312 to 5.919 ) | 3.058 ( 1.168 to 6.606 ) |  | 1199 ( 527 to 2440 ) | 2.284 ( 1.002 to 4.646 ) | 2.125 ( 0.934 to 4.324 ) | -1.12 (-1.25 to -0.99) |
| Namibia | 472 ( 325 to 715 ) | 45.16 ( 31.828 to 60.823 ) | 33.583 ( 23.18 to 50.957 ) |  | 966 ( 573 to 1463 ) | 50.508 ( 30.191 to 73.533 ) | 39.733 ( 23.571 to 60.181 ) | 0.4 (0.28 to 0.52) |
| Nauru | 0 ( 0 to 1 ) | 2.194 ( 1.109 to 4.95 ) | 2.654 ( 1.065 to 6.864 ) |  | 0 ( 0 to 1 ) | 2.235 ( 1.003 to 5.299 ) | 2.329 ( 0.91 to 6.236 ) | 0.03 (-0.23 to 0.3) |
| Nepal | 2053 ( 1056 to 4441 ) | 7.222 ( 3.916 to 14.585 ) | 10.543 ( 5.426 to 22.808 ) |  | 1326 ( 715 to 2406 ) | 4.465 ( 2.451 to 8.107 ) | 4.258 ( 2.296 to 7.73 ) | -1.25 (-1.46 to -1.04) |
| Netherlands | 433 ( 383 to 486 ) | 2.599 ( 2.296 to 2.901 ) | 2.903 ( 2.565 to 3.258 ) |  | 437 ( 346 to 533 ) | 1.677 ( 1.357 to 1.996 ) | 2.54 ( 2.012 to 3.095 ) | -1.02 (-1.17 to -0.86) |
| New Zealand | 262 ( 222 to 307 ) | 6.863 ( 5.789 to 8.052 ) | 7.669 ( 6.492 to 8.982 ) |  | 421 ( 351 to 516 ) | 5.44 ( 4.539 to 6.666 ) | 8.135 ( 6.798 to 9.975 ) | -0.53 (-0.66 to -0.4) |
| Nicaragua | 447 ( 205 to 1124 ) | 8.555 ( 4.762 to 19.059 ) | 11.507 ( 5.276 to 28.917 ) |  | 235 ( 165 to 351 ) | 3.969 ( 2.897 to 5.789 ) | 3.521 ( 2.48 to 5.261 ) | -2.03 (-2.3 to -1.75) |
| Niger | 2203 ( 984 to 5458 ) | 16.266 ( 8.005 to 38.163 ) | 27.432 ( 12.256 to 67.947 ) |  | 3562 ( 1531 to 8585 ) | 8.779 ( 4.457 to 19.559 ) | 14.226 ( 6.114 to 34.287 ) | -2.01 (-2.26 to -1.76) |
| Nigeria | 17380 ( 8840 to 25004 ) | 17.773 ( 9.744 to 23.954 ) | 19.303 ( 9.818 to 27.769 ) |  | 33678 ( 14033 to 53601 ) | 14.845 ( 7.158 to 21.673 ) | 14.569 ( 6.071 to 23.187 ) | -0.61 (-0.69 to -0.53) |
| Niue | 0 ( 0 to 0 ) | 2.308 ( 1.06 to 5.746 ) | 2.476 ( 1.057 to 6.4 ) |  | 0 ( 0 to 0 ) | 6.083 ( 2.004 to 19.209 ) | 4.655 ( 1.695 to 14.098 ) | 0.55 (-0.38 to 1.48) |
| North Macedonia | 86 ( 58 to 117 ) | 4.514 ( 3.033 to 6.097 ) | 4.319 ( 2.922 to 5.863 ) |  | 110 ( 72 to 161 ) | 3.606 ( 2.364 to 5.131 ) | 5.066 ( 3.312 to 7.389 ) | -0.87 (-1.04 to -0.69) |
| Northern Mariana Islands | 0 ( 0 to 1 ) | 0.894 ( 0.47 to 1.441 ) | 0.747 ( 0.372 to 1.272 ) |  | 0 ( 0 to 1 ) | 0.912 ( 0.453 to 1.281 ) | 0.963 ( 0.48 to 1.34 ) | 0.29 (-0.29 to 0.87) |
| Norway | 347 ( 319 to 380 ) | 5.865 ( 5.405 to 6.417 ) | 8.177 ( 7.519 to 8.96 ) |  | 109 ( 91 to 130 ) | 1.234 ( 1.041 to 1.463 ) | 2.013 ( 1.689 to 2.408 ) | -5.44 (-5.86 to -5.01) |
| Oman | 40 ( 17 to 73 ) | 2.43 ( 1.05 to 4.005 ) | 2.038 ( 0.868 to 3.664 ) |  | 43 ( 17 to 68 ) | 1.387 ( 0.544 to 2.184 ) | 0.906 ( 0.354 to 1.456 ) | -1.37 (-1.62 to -1.12) |
| Pakistan | 11030 ( 6410 to 18611 ) | 8.87 ( 5.719 to 13.205 ) | 9.926 ( 5.768 to 16.747 ) |  | 25447 ( 14523 to 39666 ) | 10.783 ( 6.618 to 15.751 ) | 10.803 ( 6.165 to 16.84 ) | 0.86 (0.71 to 1.01) |
| Palau | 0 ( 0 to 0 ) | 1.095 ( 0.44 to 2.85 ) | 1.018 ( 0.385 to 2.706 ) |  | 0 ( 0 to 0 ) | 0.999 ( 0.385 to 3.125 ) | 0.695 ( 0.302 to 1.863 ) | -0.19 (-0.33 to -0.04) |
| Palestine | 20 ( 10 to 33 ) | 1.157 ( 0.65 to 1.636 ) | 0.954 ( 0.488 to 1.621 ) |  | 32 ( 17 to 45 ) | 0.879 ( 0.473 to 1.165 ) | 0.623 ( 0.329 to 0.874 ) | -0.62 (-0.83 to -0.42) |
| Panama | 116 ( 69 to 211 ) | 4.783 ( 3.137 to 8.096 ) | 4.867 ( 2.903 to 8.834 ) |  | 125 ( 91 to 178 ) | 3.031 ( 2.163 to 4.384 ) | 2.913 ( 2.112 to 4.156 ) | -1.13 (-1.27 to -0.98) |
| Papua New Guinea | 92 ( 31 to 280 ) | 1.894 ( 0.845 to 4.83 ) | 2.251 ( 0.759 to 6.815 ) |  | 252 ( 95 to 811 ) | 2.076 ( 0.919 to 5.899 ) | 2.408 ( 0.912 to 7.755 ) | 0.3 (0.04 to 0.56) |
| Paraguay | 292 ( 150 to 508 ) | 6.355 ( 3.679 to 9.839 ) | 7.235 ( 3.722 to 12.576 ) |  | 344 ( 208 to 585 ) | 5.383 ( 3.275 to 9.08 ) | 4.797 ( 2.901 to 8.154 ) | -0.09 (-0.25 to 0.08) |
| Peru | 2811 ( 1605 to 4962 ) | 11.02 ( 6.567 to 18.593 ) | 12.993 ( 7.42 to 22.934 ) |  | 1486 ( 898 to 2375 ) | 4.429 ( 2.654 to 7.131 ) | 4.096 ( 2.476 to 6.547 ) | -2.5 (-2.65 to -2.35) |
| Philippines | 3038 ( 1202 to 4897 ) | 4.458 ( 2.107 to 6.606 ) | 4.822 ( 1.908 to 7.773 ) |  | 4830 ( 2167 to 6888 ) | 4.557 ( 2.11 to 6.386 ) | 4.265 ( 1.914 to 6.082 ) | 0.43 (0.26 to 0.6) |
| Poland | 3444 ( 3170 to 3719 ) | 8.209 ( 7.537 to 8.868 ) | 9.022 ( 8.303 to 9.742 ) |  | 2968 ( 2614 to 3336 ) | 4.575 ( 4.033 to 5.123 ) | 7.762 ( 6.836 to 8.724 ) | -2.11 (-2.43 to -1.78) |
| Portugal | 679 ( 594 to 777 ) | 6.517 ( 5.641 to 7.569 ) | 6.702 ( 5.864 to 7.669 ) |  | 650 ( 538 to 801 ) | 4.038 ( 3.319 to 4.963 ) | 6.123 ( 5.068 to 7.548 ) | -1.16 (-1.67 to -0.66) |
| Puerto Rico | 59 ( 46 to 75 ) | 1.705 ( 1.32 to 2.169 ) | 1.633 ( 1.269 to 2.067 ) |  | 60 ( 47 to 74 ) | 1.095 ( 0.86 to 1.367 ) | 1.808 ( 1.431 to 2.231 ) | -1.37 (-2.05 to -0.68) |
| Qatar | 3 ( 2 to 5 ) | 1.596 ( 0.989 to 2.503 ) | 0.625 ( 0.396 to 1.027 ) |  | 16 ( 8 to 30 ) | 1.194 ( 0.577 to 1.965 ) | 0.533 ( 0.28 to 0.994 ) | -0.42 (-0.88 to 0.05) |
| Republic of Korea | 863 ( 544 to 1186 ) | 2.376 ( 1.501 to 3.247 ) | 1.951 ( 1.23 to 2.682 ) |  | 588 ( 347 to 791 ) | 1.13 ( 0.812 to 1.458 ) | 1.14 ( 0.673 to 1.534 ) | -2.34 (-2.62 to -2.05) |
| Republic of Moldova | 314 ( 212 to 509 ) | 7.064 ( 4.722 to 11.604 ) | 7.051 ( 4.76 to 11.453 ) |  | 185 ( 154 to 222 ) | 3.682 ( 2.949 to 4.584 ) | 5.138 ( 4.277 to 6.182 ) | -2.24 (-2.43 to -2.04) |
| Romania | 1884 ( 1327 to 2693 ) | 8.149 ( 5.491 to 12.393 ) | 8.059 ( 5.676 to 11.519 ) |  | 1403 ( 1108 to 1753 ) | 4.754 ( 3.719 to 5.969 ) | 7.409 ( 5.851 to 9.258 ) | -2.17 (-2.39 to -1.95) |
| Russian Federation | 8586 ( 6829 to 10413 ) | 5.306 ( 4.223 to 6.46 ) | 5.687 ( 4.523 to 6.897 ) |  | 8651 ( 7561 to 9735 ) | 4.121 ( 3.613 to 4.628 ) | 5.972 ( 5.22 to 6.721 ) | -1.29 (-1.64 to -0.94) |
| Rwanda | 4734 ( 2912 to 8390 ) | 66.548 ( 47.609 to 97.763 ) | 65.843 ( 40.511 to 116.694 ) |  | 4573 ( 2793 to 7564 ) | 42.51 ( 27.48 to 71.608 ) | 34.463 ( 21.049 to 56.997 ) | -1.77 (-1.92 to -1.62) |
| Saint Kitts and Nevis | 0 ( 0 to 0 ) | 0.464 ( 0.389 to 0.542 ) | 0.413 ( 0.341 to 0.488 ) |  | 0 ( 0 to 0 ) | 0.24 ( 0.195 to 0.288 ) | 0.255 ( 0.207 to 0.306 ) | -1.98 (-2.19 to -1.77) |
| Saint Lucia | 1 ( 0 to 1 ) | 0.574 ( 0.474 to 0.739 ) | 0.48 ( 0.366 to 0.687 ) |  | 1 ( 1 to 1 ) | 0.392 ( 0.319 to 0.485 ) | 0.466 ( 0.38 to 0.574 ) | -1.11 (-1.33 to -0.88) |
| Saint Vincent and the Grenadines | 19 ( 12 to 35 ) | 16.57 ( 11.107 to 29.48 ) | 17.012 ( 10.558 to 31.915 ) |  | 4 ( 4 to 5 ) | 3.606 ( 2.896 to 4.589 ) | 3.809 ( 3.132 to 4.62 ) | -4.55 (-5.21 to -3.9) |
| Samoa | 11 ( 6 to 21 ) | 8.355 ( 4.808 to 18.356 ) | 6.712 ( 3.765 to 12.385 ) |  | 14 ( 8 to 27 ) | 7.568 ( 4.324 to 15.703 ) | 6.538 ( 3.632 to 12.724 ) | -0.25 (-0.33 to -0.18) |
| San Marino | 1 ( 1 to 1 ) | 2.85 ( 2.01 to 3.966 ) | 3.815 ( 2.684 to 5.368 ) |  | 1 ( 1 to 2 ) | 1.631 ( 0.934 to 2.643 ) | 2.988 ( 1.725 to 4.778 ) | -0.89 (-1.2 to -0.58) |
| Sao Tome and Principe | 21 ( 9 to 48 ) | 11.878 ( 5.894 to 26.001 ) | 16.988 ( 7.059 to 39.976 ) |  | 14 ( 6 to 30 ) | 6.607 ( 3.266 to 12.891 ) | 6.63 ( 2.848 to 14.046 ) | -1.64 (-2.38 to -0.9) |
| Saudi Arabia | 448 ( 88 to 956 ) | 2.695 ( 0.753 to 4.8 ) | 2.825 ( 0.558 to 6.026 ) |  | 440 ( 157 to 694 ) | 1.523 ( 0.565 to 2.395 ) | 1.166 ( 0.417 to 1.84 ) | -1.38 (-2.09 to -0.66) |
| Senegal | 1706 ( 724 to 3959 ) | 14.267 ( 7.074 to 30.301 ) | 22.346 ( 9.48 to 51.871 ) |  | 1700 ( 761 to 3764 ) | 8.815 ( 4.521 to 17.785 ) | 10.718 ( 4.799 to 23.733 ) | -1.08 (-1.44 to -0.72) |
| Serbia | 571 ( 358 to 762 ) | 5.549 ( 3.445 to 7.421 ) | 5.932 ( 3.719 to 7.911 ) |  | 586 ( 344 to 781 ) | 3.913 ( 2.395 to 5.237 ) | 6.568 ( 3.857 to 8.761 ) | -1.34 (-1.48 to -1.2) |
| Seychelles | 1 ( 1 to 2 ) | 2.063 ( 1.173 to 2.697 ) | 1.662 ( 0.979 to 2.192 ) |  | 2 ( 1 to 2 ) | 1.447 ( 0.822 to 1.871 ) | 1.632 ( 0.893 to 2.138 ) | -0.83 (-0.92 to -0.74) |
| Sierra Leone | 752 ( 329 to 1563 ) | 12.082 ( 5.92 to 23.762 ) | 18.123 ( 7.926 to 37.652 ) |  | 870 ( 362 to 1988 ) | 7.8 ( 3.782 to 16.281 ) | 9.811 ( 4.084 to 22.417 ) | -1.19 (-1.41 to -0.97) |
| Singapore | 75 ( 60 to 92 ) | 3.423 ( 2.675 to 4.303 ) | 2.46 ( 1.974 to 3.006 ) |  | 65 ( 53 to 81 ) | 1.28 ( 0.968 to 1.676 ) | 1.137 ( 0.923 to 1.408 ) | -3 (-3.54 to -2.46) |
| Slovakia | 356 ( 240 to 470 ) | 6.144 ( 4.198 to 8.058 ) | 6.735 ( 4.536 to 8.888 ) |  | 365 ( 236 to 507 ) | 4.342 ( 2.805 to 5.939 ) | 6.723 ( 4.347 to 9.334 ) | -1.04 (-1.11 to -0.97) |
| Slovenia | 91 ( 71 to 111 ) | 3.892 ( 3.094 to 4.754 ) | 4.59 ( 3.604 to 5.614 ) |  | 74 ( 53 to 98 ) | 1.976 ( 1.457 to 2.595 ) | 3.57 ( 2.562 to 4.717 ) | -2.01 (-2.15 to -1.87) |
| Solomon Islands | 8 ( 2 to 23 ) | 1.845 ( 0.77 to 4.508 ) | 2.285 ( 0.661 to 6.8 ) |  | 16 ( 6 to 46 ) | 2.13 ( 0.997 to 5.427 ) | 2.355 ( 0.911 to 6.694 ) | 0.56 (0.14 to 0.98) |
| Somalia | 4565 ( 2633 to 8548 ) | 63.591 ( 43.425 to 99.758 ) | 57.506 ( 33.168 to 107.675 ) |  | 8687 ( 4116 to 16500 ) | 52.961 ( 30.664 to 88.772 ) | 40.205 ( 19.051 to 76.366 ) | -0.47 (-0.63 to -0.32) |
| South Africa | 1503 ( 962 to 2192 ) | 4.288 ( 2.885 to 5.946 ) | 4.06 ( 2.6 to 5.923 ) |  | 2545 ( 1406 to 3663 ) | 4.976 ( 2.738 to 7.105 ) | 4.477 ( 2.474 to 6.444 ) | 0.69 (0.45 to 0.92) |
| South Sudan | 2740 ( 1575 to 4675 ) | 49.54 ( 32.696 to 78.072 ) | 46.622 ( 26.799 to 79.544 ) |  | 3350 ( 1988 to 5438 ) | 43.264 ( 27.331 to 68.36 ) | 34.633 ( 20.552 to 56.225 ) | -0.34 (-0.49 to -0.19) |
| Spain | 1774 ( 1597 to 1984 ) | 3.912 ( 3.518 to 4.357 ) | 4.575 ( 4.117 to 5.115 ) |  | 2243 ( 1843 to 2707 ) | 2.996 ( 2.473 to 3.627 ) | 4.924 ( 4.045 to 5.942 ) | -0.49 (-0.75 to -0.23) |
| Sri Lanka | 432 ( 297 to 656 ) | 2.868 ( 2.078 to 4.145 ) | 2.525 ( 1.734 to 3.828 ) |  | 407 ( 243 to 591 ) | 1.757 ( 1.073 to 2.618 ) | 1.827 ( 1.09 to 2.654 ) | -1.22 (-1.5 to -0.93) |
| Sudan | 60 ( 13 to 191 ) | 0.293 ( 0.058 to 0.922 ) | 0.298 ( 0.065 to 0.952 ) |  | 84 ( 19 to 258 ) | 0.254 ( 0.053 to 0.788 ) | 0.194 ( 0.043 to 0.593 ) | -0.25 (-0.43 to -0.07) |
| Suriname | 7 ( 4 to 9 ) | 2.144 ( 1.528 to 2.978 ) | 1.696 ( 1.138 to 2.329 ) |  | 13 ( 8 to 17 ) | 2.099 ( 1.302 to 2.818 ) | 2.219 ( 1.375 to 2.988 ) | 0.24 (0.06 to 0.42) |
| Sweden | 293 ( 251 to 344 ) | 2.274 ( 1.963 to 2.67 ) | 3.41 ( 2.921 to 4.001 ) |  | 562 ( 438 to 701 ) | 3.058 ( 2.395 to 3.782 ) | 5.418 ( 4.222 to 6.758 ) | 1.66 (1.33 to 1.99) |
| Switzerland | 312 ( 270 to 368 ) | 3.628 ( 3.135 to 4.239 ) | 4.55 ( 3.931 to 5.357 ) |  | 624 ( 482 to 759 ) | 4.326 ( 3.386 to 5.265 ) | 6.994 ( 5.403 to 8.501 ) | 0.52 (0.28 to 0.75) |
| Syrian Arab Republic | 28 ( 19 to 38 ) | 0.355 ( 0.255 to 0.524 ) | 0.217 ( 0.149 to 0.299 ) |  | 43 ( 27 to 64 ) | 0.317 ( 0.205 to 0.473 ) | 0.303 ( 0.194 to 0.459 ) | -0.47 (-0.59 to -0.34) |
| Taiwan (Province of China) | 325 ( 292 to 363 ) | 1.887 ( 1.691 to 2.113 ) | 1.593 ( 1.43 to 1.78 ) |  | 315 ( 252 to 380 ) | 1.283 ( 0.983 to 1.652 ) | 1.331 ( 1.066 to 1.607 ) | -0.13 (-0.62 to 0.36) |
| Tajikistan | 65 ( 13 to 146 ) | 0.752 ( 0.171 to 1.695 ) | 1.208 ( 0.239 to 2.725 ) |  | 136 ( 27 to 406 ) | 1.053 ( 0.218 to 3.13 ) | 1.337 ( 0.267 to 3.996 ) | 1.61 (1.27 to 1.95) |
| Thailand | 1777 ( 1123 to 2569 ) | 3.594 ( 2.292 to 5.087 ) | 3.13 ( 1.978 to 4.526 ) |  | 1469 ( 932 to 2046 ) | 1.938 ( 1.415 to 2.686 ) | 2.202 ( 1.398 to 3.069 ) | -2.21 (-2.45 to -1.97) |
| Timor-Leste | 20 ( 7 to 43 ) | 2.217 ( 1.017 to 4.171 ) | 2.594 ( 0.921 to 5.519 ) |  | 29 ( 12 to 58 ) | 2.107 ( 0.935 to 3.762 ) | 2.103 ( 0.854 to 4.146 ) | -0.13 (-0.33 to 0.08) |
| Togo | 604 ( 282 to 1415 ) | 11.354 ( 6.2 to 24.283 ) | 16.574 ( 7.746 to 38.8 ) |  | 717 ( 319 to 1647 ) | 7.476 ( 3.865 to 15.374 ) | 8.561 ( 3.808 to 19.673 ) | -1.32 (-1.46 to -1.18) |
| Tokelau | 0 ( 0 to 0 ) | 1.69 ( 0.941 to 3.32 ) | 1.723 ( 0.875 to 3.771 ) |  | 0 ( 0 to 1 ) | 33.684 ( 7.607 to 109.579 ) | 26.005 ( 6.041 to 84.25 ) | 2.56 (-0.2 to 5.4) |
| Tonga | 2 ( 1 to 4 ) | 1.472 ( 0.75 to 3.363 ) | 1.63 ( 0.621 to 4.447 ) |  | 2 ( 1 to 5 ) | 1.564 ( 0.744 to 3.745 ) | 1.758 ( 0.758 to 4.666 ) | 0.26 (0.01 to 0.51) |
| Trinidad and Tobago | 50 ( 29 to 92 ) | 4.063 ( 2.551 to 7.178 ) | 4.117 ( 2.407 to 7.648 ) |  | 37 ( 24 to 57 ) | 3.234 ( 1.874 to 5.436 ) | 2.683 ( 1.75 to 4.091 ) | -0.07 (-0.78 to 0.63) |
| Tunisia | 383 ( 233 to 664 ) | 4.295 ( 2.813 to 7.037 ) | 4.586 ( 2.787 to 7.948 ) |  | 271 ( 174 to 403 ) | 2.388 ( 1.556 to 3.599 ) | 2.291 ( 1.472 to 3.399 ) | -2 (-2.12 to -1.87) |
| Turkey | 1711 ( 1111 to 2602 ) | 3.151 ( 2.153 to 4.664 ) | 2.978 ( 1.934 to 4.527 ) |  | 1550 ( 1123 to 2091 ) | 1.973 ( 1.423 to 2.668 ) | 1.854 ( 1.343 to 2.5 ) | -1.52 (-1.64 to -1.41) |
| Turkmenistan | 138 ( 86 to 220 ) | 4.396 ( 2.978 to 6.278 ) | 3.738 ( 2.317 to 5.934 ) |  | 170 ( 105 to 277 ) | 3.587 ( 2.221 to 5.774 ) | 3.289 ( 2.029 to 5.369 ) | -0.9 (-1.02 to -0.79) |
| Tuvalu | 0 ( 0 to 1 ) | 2.471 ( 1.179 to 5.709 ) | 3.004 ( 1.217 to 7.924 ) |  | 0 ( 0 to 1 ) | 1.758 ( 0.873 to 4.207 ) | 1.695 ( 0.808 to 4.219 ) | -0.87 (-1.13 to -0.62) |
| Uganda | 9897 ( 5916 to 15399 ) | 65.162 ( 42.582 to 86.856 ) | 57.241 ( 34.214 to 89.057 ) |  | 18670 ( 10736 to 30263 ) | 60.975 ( 37.527 to 87.477 ) | 43.102 ( 24.786 to 69.866 ) | -0.46 (-0.58 to -0.34) |
| Ukraine | 2984 ( 2450 to 3653 ) | 4.549 ( 3.733 to 5.612 ) | 5.66 ( 4.647 to 6.93 ) |  | 2728 ( 1900 to 3715 ) | 4.349 ( 3.066 to 5.758 ) | 6.333 ( 4.411 to 8.624 ) | -0.25 (-0.42 to -0.07) |
| United Arab Emirates | 32 ( 19 to 54 ) | 3.532 ( 2.279 to 6.089 ) | 1.704 ( 1.036 to 2.904 ) |  | 107 ( 74 to 203 ) | 2.92 ( 2.106 to 4.848 ) | 1.11 ( 0.77 to 2.109 ) | 1.17 (0.61 to 1.74) |
| United Kingdom | 4726 ( 4506 to 4955 ) | 5.938 ( 5.67 to 6.209 ) | 8.248 ( 7.865 to 8.647 ) |  | 3765 ( 3486 to 4029 ) | 3.534 ( 3.299 to 3.783 ) | 5.549 ( 5.137 to 5.938 ) | -1.22 (-1.64 to -0.79) |
| United Republic of Tanzania | 14686 ( 8304 to 26371 ) | 56.681 ( 38.995 to 83.779 ) | 56.839 ( 32.141 to 102.067 ) |  | 22397 ( 12883 to 38903 ) | 42.789 ( 27.277 to 69.545 ) | 38.319 ( 22.041 to 66.56 ) | -0.79 (-0.87 to -0.71) |
| United States of America | 9240 ( 8740 to 9786 ) | 3.318 ( 3.138 to 3.515 ) | 3.637 ( 3.44 to 3.851 ) |  | 12265 ( 11310 to 13212 ) | 2.538 ( 2.345 to 2.747 ) | 3.687 ( 3.4 to 3.972 ) | -0.77 (-1.03 to -0.51) |
| United States Virgin Islands | 3 ( 1 to 7 ) | 2.984 ( 1.291 to 6.449 ) | 3.003 ( 1.27 to 6.585 ) |  | 1 ( 0 to 1 ) | 1.156 ( 0.567 to 2.276 ) | 0.823 ( 0.538 to 1.337 ) | -3.02 (-3.35 to -2.69) |
| Uruguay | 150 ( 120 to 189 ) | 4.593 ( 3.571 to 5.984 ) | 4.774 ( 3.808 to 6.016 ) |  | 128 ( 105 to 158 ) | 3.053 ( 2.479 to 3.85 ) | 3.75 ( 3.071 to 4.643 ) | -1.47 (-1.7 to -1.23) |
| Uzbekistan | 542 ( 262 to 1166 ) | 2.555 ( 1.459 to 4.557 ) | 2.588 ( 1.248 to 5.562 ) |  | 1024 ( 627 to 1627 ) | 3.087 ( 1.96 to 4.791 ) | 2.99 ( 1.832 to 4.753 ) | 0.6 (0.32 to 0.88) |
| Vanuatu | 3 ( 1 to 8 ) | 1.516 ( 0.757 to 3.437 ) | 1.783 ( 0.658 to 5.15 ) |  | 5 ( 2 to 13 ) | 1.563 ( 0.818 to 3.475 ) | 1.607 ( 0.722 to 4.201 ) | 0.48 (0.06 to 0.9) |
| Venezuela (Bolivarian Republic of) | 1049 ( 714 to 1672 ) | 5.973 ( 4.593 to 8.515 ) | 5.578 ( 3.798 to 8.892 ) |  | 1126 ( 805 to 1629 ) | 4.232 ( 2.986 to 6.303 ) | 4.228 ( 3.021 to 6.117 ) | -0.96 (-1.06 to -0.85) |
| Viet Nam | 6707 ( 3713 to 12622 ) | 7.801 ( 4.51 to 13.973 ) | 9.83 ( 5.442 to 18.5 ) |  | 3684 ( 2311 to 5834 ) | 4.103 ( 2.504 to 6.703 ) | 3.674 ( 2.304 to 5.819 ) | -2.22 (-2.33 to -2.11) |
| Yemen | 33 ( 7 to 104 ) | 0.258 ( 0.048 to 0.846 ) | 0.241 ( 0.05 to 0.764 ) |  | 66 ( 14 to 202 ) | 0.269 ( 0.058 to 0.826 ) | 0.195 ( 0.043 to 0.602 ) | 0.26 (0.08 to 0.45) |
| Zambia | 4600 ( 2725 to 7815 ) | 56.9 ( 39.203 to 82.849 ) | 57.966 ( 34.334 to 98.473 ) |  | 8368 ( 4513 to 14190 ) | 59.562 ( 32.166 to 104.307 ) | 42.88 ( 23.126 to 72.712 ) | 0.33 (0.2 to 0.46) |
| Zimbabwe | 1398 ( 884 to 1968 ) | 20.449 ( 12.843 to 26.695 ) | 13.512 ( 8.543 to 19.031 ) |  | 4165 ( 1766 to 6797 ) | 37.714 ( 15.707 to 56.525 ) | 26.708 ( 11.323 to 43.584 ) | 3.27 (2.36 to 4.19) |
